# A genome-wide *Drosophila* epithelial tumorigenesis screen identifies Tetraspanin 29Fb as an evolutionarily conserved suppressor of Ras-driven cancer

**DOI:** 10.1371/journal.pgen.1007688

**Published:** 2018-10-16

**Authors:** Tamara Zoranovic, Jan Manent, Lee Willoughby, Ricardo Matos de Simoes, John E. La Marca, Sofya Golenkina, Xia Cuiping, Susanne Gruber, Belinda Angjeli, Elisabeth Eva Kanitz, Shane J. F. Cronin, G. Gregory Neely, Andreas Wernitznig, Patrick O. Humbert, Kaylene J. Simpson, Constantine S. Mitsiades, Helena E. Richardson, Josef M. Penninger

**Affiliations:** 1 IMBA, Institute of Molecular Biotechnology of the Austrian Academy of Science, Campus Vienna BioCentre, Vienna, Austria; 2 Research Division, Peter MacCallum Cancer Centre, Melbourne, Victoria, Australia; 3 Department of Biochemistry & Genetics, La Trobe Institute for Molecular Science, La Trobe University, Melbourne, Victoria, Australia; 4 Department of Medical Oncology, Dana-Farber Cancer Institute, Harvard Medical School, Boston, Massachusetts, United States of America; 5 The Charles Perkins Centre, School of Life & Environmental Sciences, The University of Sydney, Sydney, New South Wales, Australia; 6 Boehringer Ingelheim RCV GmbH & Co KG, Vienna, Austria; 7 Sir Peter MacCallum Department of Oncology, Department of Anatomy & Neuroscience, Department of Biochemistry & Molecular Biology, and Department of Clinical Pathology, University of Melbourne, Melbourne, Victoria, Australia; 8 Victorian Center for Functional Genomics, Peter MacCallum Cancer Centre, Melbourne, Victoria, Australia; Harvard Medical School, Howard Hughes Medical Institute, UNITED STATES

## Abstract

Oncogenic mutations in the small GTPase Ras contribute to ~30% of human cancers. However, Ras mutations alone are insufficient for tumorigenesis, therefore it is paramount to identify cooperating cancer-relevant signaling pathways. We devised an *in vivo* near genome-wide, functional screen in *Drosophila* and discovered multiple novel, evolutionarily-conserved pathways controlling Ras-driven epithelial tumorigenesis. Human gene orthologs of the fly hits were significantly downregulated in thousands of primary tumors, revealing novel prognostic markers for human epithelial tumors. Of the top 100 candidate tumor suppressor genes, 80 were validated in secondary *Drosophila* assays, identifying many known cancer genes and multiple novel candidate genes that cooperate with Ras-driven tumorigenesis. Low expression of the confirmed hits significantly correlated with the *KRAS*^*G12*^ mutation status and poor prognosis in pancreatic cancer. Among the novel top 80 candidate cancer genes, we mechanistically characterized the function of the top hit, the Tetraspanin family member Tsp29Fb, revealing that Tsp29Fb regulates EGFR signaling, epithelial architecture and restrains tumor growth and invasion. Our functional *Drosophila* screen uncovers multiple novel and evolutionarily conserved epithelial cancer genes, and experimentally confirmed Tsp29Fb as a key regulator of EGFR/Ras induced epithelial tumor growth and invasion.

## Author summary

Cancer involves the cooperative interaction of many gene mutations. The Ras signaling pathway is upregulated in many human cancers, but upregulated Ras signaling alone is not sufficient to induce malignant tumors. We have undertaken a genome-wide genetic screen using a transgenic *RNAi* library in the vinegar fly, *Drosophila melanogaster*, to identify tumor suppressor genes that cooperate with the Ras oncogene (*Ras*^*V12*^) in conferring overgrown invasive tumors. We stratified the hits by analyzing the expression of human orthologs of these genes in human epithelial cancers, revealing genes that were strongly downregulated in human cancer. By conducting secondary genetic interaction tests, we validated 80 of the top 100 genes. Pathway analysis of these genes revealed that 55 fell into known pathways involved in human cancer, whereas 25 were unique genes. We then confirmed the tumor suppressor properties of one of these genes, *Tsp29Fb*, encoding a Tetraspanin membrane protein, and showed that Tsp29Fb functions as a tumor suppressor by inhibiting Ras signaling and by maintaining epithelial cell polarity. Altogether, our study has revealed novel Ras-cooperating tumor suppressors in *Drosophila* and suggests that these genes may also be involved in human cancer.

## Introduction

Mutations in *Ha-RAS*, *N-RAS*, and *K-RAS* genes are frequent in human tumors and represent oncogenic drivers of cell proliferation and survival. However, tissue growth induced by oncogenic Ras is restrained by the induction of cellular senescence [1, 2] and additional mutations are needed to promote tumorigenesis and metastasis [3]. Metastatic dissemination represents a complex process [4, 5] that appears to be regulated via multiple biological pathways [6, 7]. Considering millions of cancer patients, in particular in an aging population, it is, therefore, paramount to identify novel pathways and genes involved in cancer growth and metastasis.

Many developmental pathways and signaling cascades were originally identified in *Drosophila melanogaster* [8, 9]. Among the most studied conserved signal transduction pathways involved in tumorigenesis are Ras and Notch [8, 10–12]. In landmark studies, invasion-metastasis has also been successfully modeled in fly larvae [13]. Using the *Drosophila* system, cell polarity genes were identified as suppressors of Ras-driven tumor growth and invasion [14, 15] implicating high inter-species conservation of fundamental genetic networks controlling transformation and metastasis [16]. Given the accessibility of *Drosophila* to *in vivo RNAi* screening approaches and conservation of major developmental pathways including Ras, the fly offers a valuable opportunity to genetically dissect cancer-relevant signaling networks in the context of a whole animal, which could lead to the identification of novel biomarkers and new drug targets for a diverse range of epithelial tumors. Moreover, whereas mammals have three *RAS* genes, *K-*, *N-* and *Ha-Ras*, the *Drosophila* has only two: *D-Ras1* or *Ras85D* and *D-Ras2* or *Ras64B* [17]. *Ras85D* has been shown to be the authentic human ortholog [18, 19], making the fly a suitable model system to genetically dissect Ras-regulated networks.

One of the most frequent *Ras* mutations is *Ras*^*V12*^, an activating point mutation in codon 12 [20, 21]. To identify novel conserved genes involved in *Ras*^*V12*^-driven tumor progression and metastases, we performed a *dRas* (*Ras85D*^*V12*^)-driven tumorigenesis screen using *Drosophila* genetics, stratified the mammalian orthologs of the fly hits in thousands of human cancers, and further validated the top 100 candidate tumor suppressor genes in secondary *Drosophila* assays. Our screen identified many known cancer genes and multiple novel, evolutionary conserved candidate genes that cooperate with Ras in tumorigenesis. We also validated and analyzed the mechanism of action of one of these novel tumor suppressors, the Tetraspanin *Tsp29Fb*, showing that it inhibits Ras signaling and acts to maintain epithelial tissue architecture.

## Results

### An *in vivo* high throughput assay for progression and metastases of *dRas*^*V12*^ induced epithelial tumors in *Drosophila*

**Fig 1:**
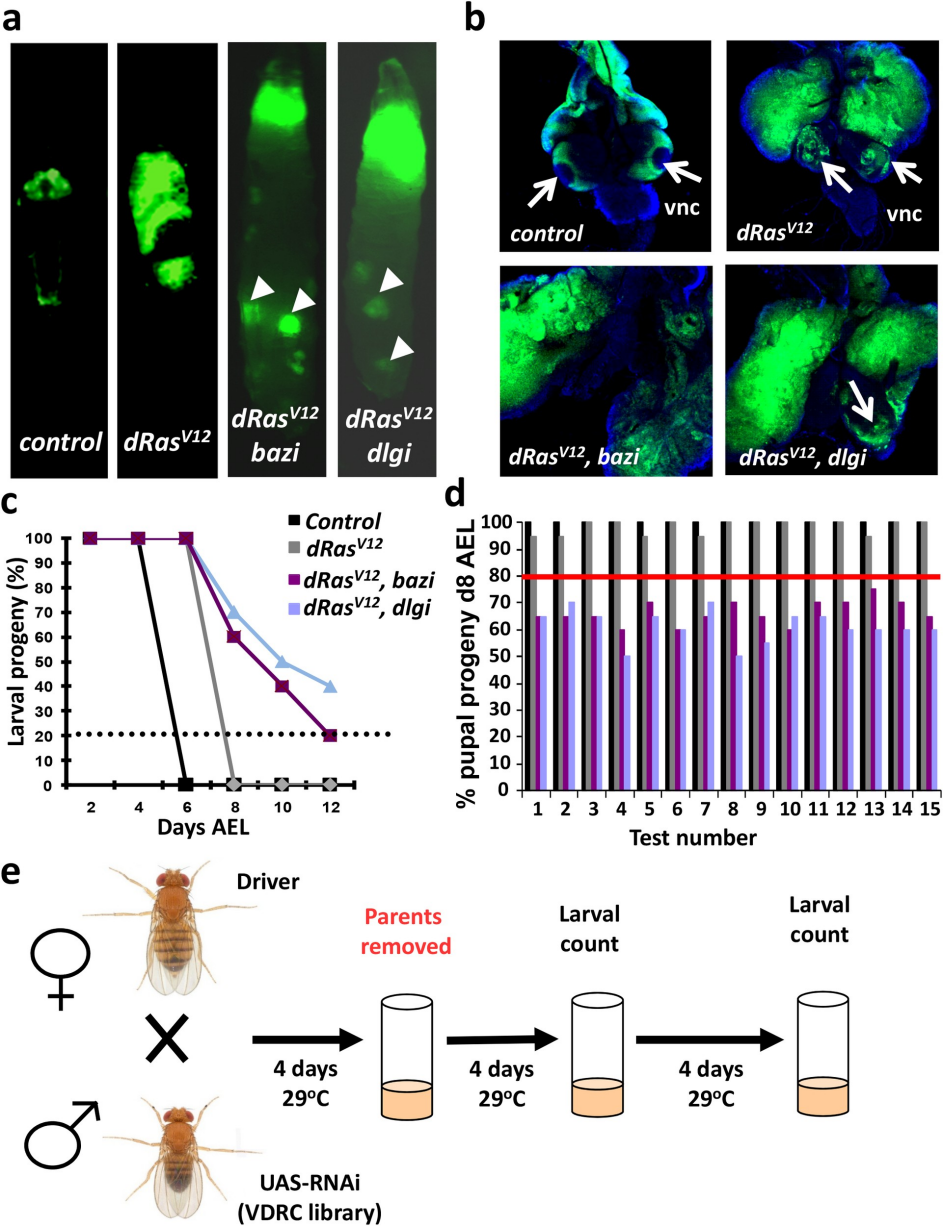
High through-put screen for *dRas*^*V12*^-driven epithelial tumorigenesis in *Drosophila*. **(a)** GFP^+^ eye disc cells in control, *dRas*^*V12*^, *dRas*^*V12*^*;baz-RNAi (bazi)*, and *dRas*^*V12*^*;dlg-RNAi* (*dlgi*) flies. Ectopic GFP+ foci (arrowheads) in *dRas*^*V12*^*;baz-RNAi (bazi)*, and *dRas*^*V12*^*;dlg-RNAi* (*dlgi*) observed on day 8 after egg laying (AEL) at 29°C. Magnifications X 8. **(b)** Normal and *dRas*^*V12*^-transformed eye discs at d5 AEL (top panels). Invasive disc tumors appearing upon co-expression of *baz-RNAi* or *dlg-RNAi* fuse with the brain lobes (arrows) in d8 AEL larvae (bottom panels). Magnifications X 25 (top panels) and X 40 (bottom panels). vnc, ventral nerve cord. **(c)** Quantification of larval arrest (% larval progeny at the indicated days AEL) and **(d)** independent test results for assay optimisation. The blue dotted line in (C) and the red line in (D) indicate the cut-off (Z-score 1.65) for phenotype detection. **(e)** Schematic of the screen design. Virgin driver line females (*ey-Flp; Act5C>>GAL4*, *UAS-GFP/CyO*, *Tub-GAL80; UAS-dRas^V12^*) were crossed to *UAS-RNAi* transgenic males. All crosses were performed at 29°C and parents removed after 4 days. Counts of the larval progeny were performed at two time points, d8 and d12 AEL, and compared to larval offspring from isogenic *w^1118^* control males crossed to driver females.

To identify genes involved specifically in Ras-driven epithelial cancer development, we designed a high-throughput *in vivo RNAi* screening system in *Drosophila* that enabled us to assay a large number of previously unexplored genes in tumorigenesis *in vivo*, and to monitor tumor formation, invasion, and formation of distant site metastases. *dRas*^*V12*^ (*Ras85D*^*V12*^), GFP and *RNAi* expression were targeted to the larval eye disc epithelium using the *eyeless (ey)-FLP; actin flip-out* system [22, 23]. This enables monitoring of stable GFP-labelled tumors in transparent third instar larvae. Overexpression of *Drosophila dRas*^*V12*^ in developing eye discs resulted in GFP-labelled epithelial hyperplasia (Fig 1A and 1B) and ultimately death at the pupal stage. To exclude the possibility that defective larval-pupal transition was due to tumor formation in the ecdysone-producing larval ring gland, we showed that the *ey* driver was not expressed in the ring gland (S1A Fig). To rule out the possibility that the observed phenotypes might be due to genetic load of the experimental line, we showed that expression of a dominant negative *dRaf* transgene was able to rescue pupal lethality in both male and female *dRas*^*V12*^ bearing flies (S1B Fig), indicating that larval delay is a consequence of dRas^V12^-induced overgrowth of the larval eye disc.

To test whether *RNAi*-mediated knockdown can identify suppressors of epithelial tumor progression, we assayed *Drosophila* genes previously reported to have this function [14, 15, 24]; knockdown of Discs-large (Dlg) and Bazooka (Baz) resulted in large tumor formation with ectopic GFP^+^ foci (Fig 1A) and ventral nerve cord invasion (Fig 1B). To establish the cut-off criteria for selecting valid hits from the screen we analysed multiple tests of control and *dRas*^*V12*^ larvae (n = 600 each), which revealed complete pupariation on day 8 after egg laying (AEL), a 2–3 day delay relative to the control, with a mean pupal progeny percentage of 100% ± 0.0 SD for control and 98.8% ± 5 SD for *dRas*^*V12*^ flies (Fig 1C). In contrast, co-expression of *dlg-RNAi* or *baz-RNAi* with *dRas*^*V12*^ (n = 600 each) led to defective pupariation (with larval progeny present at 66.64% ± 7.58 SD and 62.5% ± 5.98 SD, respectively) on day 8 AEL (Fig 1C). Based on these data, we set our primary screen cut-off at the 80-percentile probability of pupariation corresponding to a Z-score of > 1.65. At this threshold, we consistently identified *baz* and *dlg RNAi* lines in multiple blinded tests (Fig 1D). Thus, our experimental set-up can be used to identify genes that modulate progression of *dRas*^*V12*^-induced tumors.

### Genome-wide screen for genes involved in tumor growth and metastasis

Using this *Drosophila* set-up, we screened for novel modulators of *dRas*^*V12*^ -driven epithelial tumor progression. Female *Drosophila* carrying the *dRas*^*V12*^ oncogene were crossed to male flies containing *UAS-RNAi* transgenes (from the GD *RNAi* library from the Vienna *Drosophila* Resource Center) resulting in progeny that express *dRas*^*V12*^ and the respective *RNAi* transgenes specifically in the developing eye-antenna disc epithelium (Fig 1E). We choose to study the eye-antenna disc because it constitutes an epithelial monolayer where proliferation, differentiation and tissue structure can be easily analyzed [20, 25]. This allowed us to screen for enhanced growth of the *dRas*^*V12*^-driven epithelial tumors using delayed pupariation on day 8 AEL as a high throughput read-out. In addition to larval arrest, we recorded metastatic events, namely invasion of the tumors anteriorly to the larval mouth hooks and ectopic GFP signals for all *UAS-RNAi* lines assayed. We excluded *RNAi* hairpins with insufficient targeting specificity, i.e., with more than six Can repeats and/or S19 score <0.8 leaving 10,720 *UAS-RNAi* lines corresponding to 6,675 evolutionarily conserved genes for the screen (S1 Table). Using our selection criteria, we identified 951 *Drosophila* candidate genes that when down-regulated resulted in larval arrest and/or local invasion of the tumor anteriorly to the larval mouth hook and distant foci of GFP-labelled eye disc epithelial cells (S2 Table). Thus, our screen identified multiple *Drosophila* candidate genes that when downregulated via *RNAi* potentially promote *dRas*^*V12*^-induced *in vivo* epithelial tumorigenesis.

### Evolutionary conservation of Ras-modifying genes

**Fig 2:**
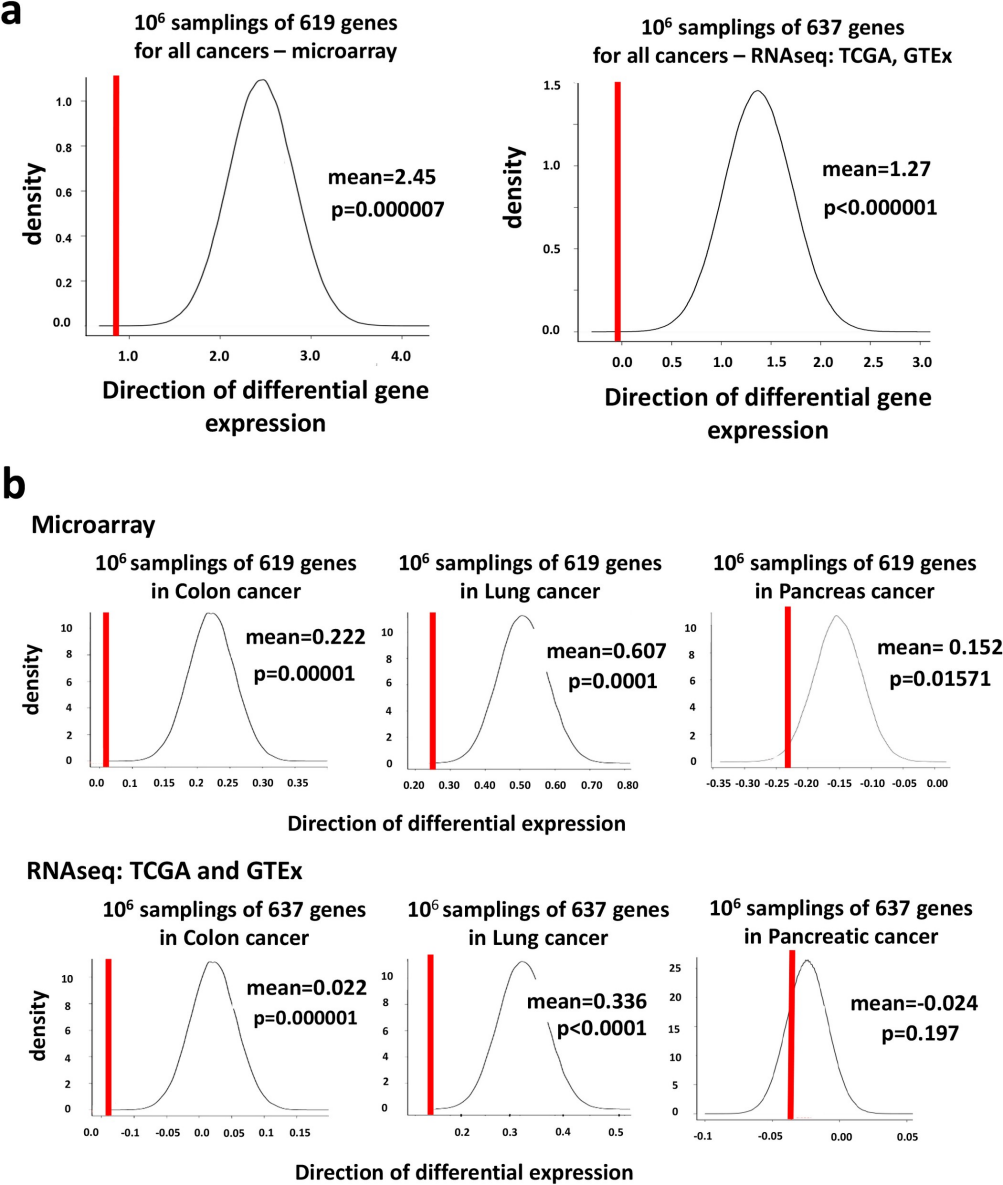
Tumor-suppressor-oncogene-scores (TSOS). **(a)** Enrichment analysis using expression derived tumor-suppressor-oncogene-score (TSOS) in human tumors microarray and TCGA/GTEx RNAseq datasets for the whole candidate ortholog gene set (n = 619 and n = 637 respectively) in 1 million random samplings. The probability to find a list with TSOS score in the respective datasets equivalent to our experimentally obtained one is p = 7x10^-6^ and p = 0. **(b)** Tumor suppressor oncogene scores (TSOS) to assess gene expression of our candidate hits in malignancies of the indicated tissues compared to the corresponding normal tissues for all human orthologs of our fly hits (red line) versus the median distribution of gene expression of randomly chosen gene sets (numbers of samplings are indicated for each tumor, n = 619 genes for microarray gene sets and n = 637 for the TCGA and GTEx RNAseq data sets). Only human orthologs of all tested fly genes (S1 Table) were included to avoid bias of comparison. Positive values represent a higher expression in tumors, a negative TSOS shows a higher expression in normal tissues on average. For data on gene expression in tumors and normal tissue see S3 Table. P values are indicated for each tumor type.

To determine whether our *Drosophila* tumor screen results are translatable to human cancers, we performed differential mRNA expression analysis of the corresponding human orthologs in multiple tumors and matched normal tissues of 3351 patients (S3 Table). Among 637 predicted human orthologs, we could derive data for 619 genes, which were present on the respective gene expression arrays. A large number of genes identified exhibited reduced expression in primary human cancers compared to the respective matching normal tissue (S3 Table). As a control, we also analyzed the mRNA expression profiles of one million randomly selected sets of 619 genes. Compared to these expression profiles, our experimentally-obtained candidate list was significantly enriched for conserved genes with lower expression in all human malignancies tested (p<0.000007; Fig 2A).

The strongest enrichment for low tumor expression was observed in tumors with frequent Ras activating mutations, such as in colon, lung and pancreatic cancer (Fig 2B). In line with our screen set-up, significant enrichment of hits with low tumor expression was observed almost exclusively in solid malignancies, indicating their conserved function in control of epithelial transformation (Fig 2B and S2 Fig). Using public mRNA sequencing data from human tumors and normal tissues (TCGA and GTEx), we were able to retrieve expression data for all 637 candidate genes. Analysis of TCGA and GTEx again showed that the identified fly genes were *in toto* significantly downregulated in human cancer (Fig 2A and 2B), confirming the microarray data. Of note, although the 637 gene set was significantly downregulated in colon and lung TCGA and GTEx datasets (Fig 2B), we did not observe significant enrichment for genes downregulated in the TCGA pancreatic ductal carcinoma data set, which might be explained by the low tumor sample number (n = 179). Thus, the mammalian orthologs of our fly candidate genes are strongly under-expressed in multiple human cancers, in particular in tumors with the highest frequency of Ras activating mutations.

### Validation of top hits in secondary *Drosophila* screens

**Fig 3:**
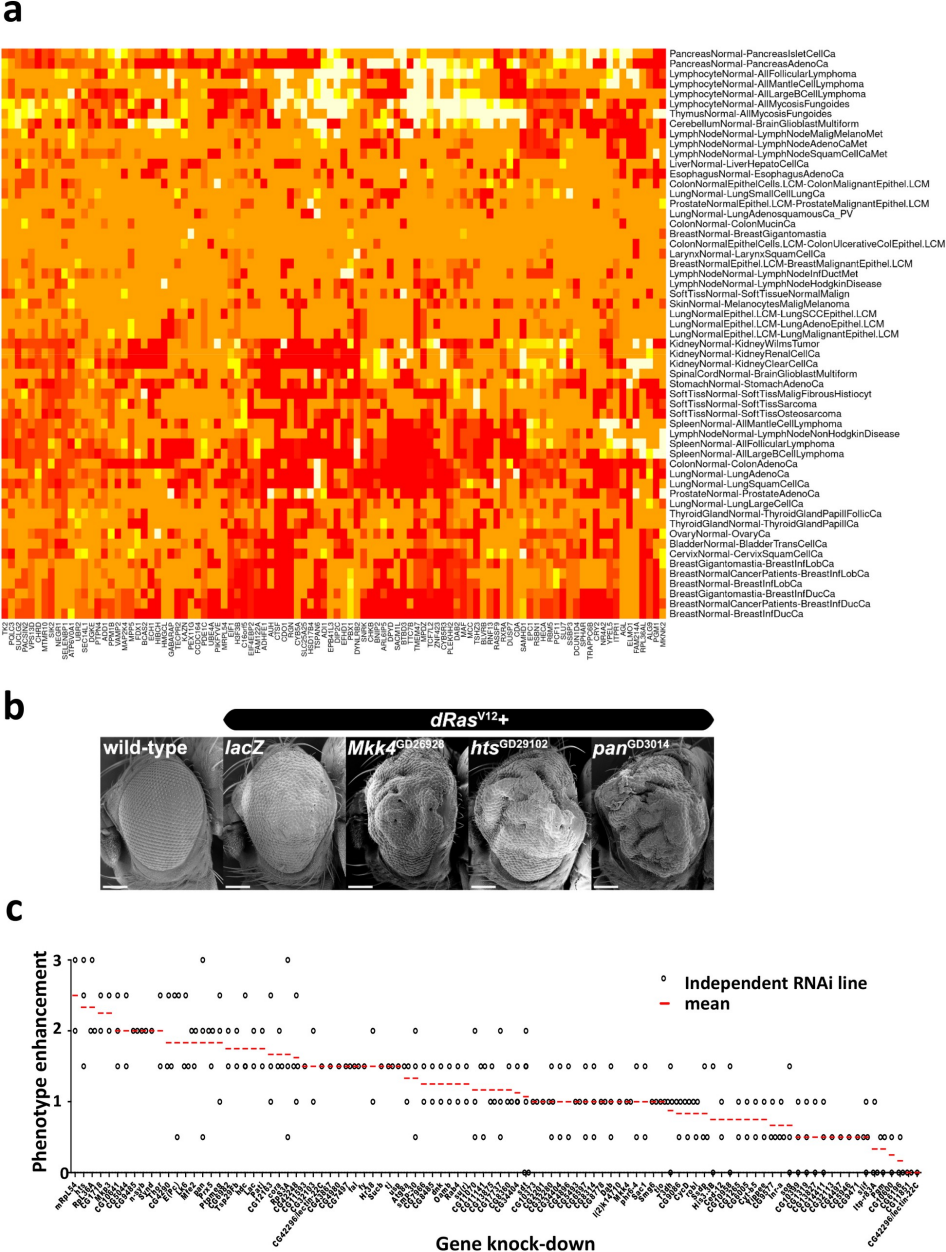
Evolutionary conservation of Ras modifying genes. **(a)** Heat map for the top 100 human ortholog candidate tumor modifier genes selected based on the highest TSOS score values, tested for differential tumor and matching normal tissue expression (log2 values equivalent to fold change) color legend: red = lower expression in tumors, orange = no significant difference, white and yellow = increased expression in tumors. **(b)** Representative scanning electron micrographs of selected eye phenotypes scored in a secondary screen for *dRas*^*V12*^-cooperative eye overgrowth. Genotypes from top left: wild-type, *ey>dRas*^*V12*^*/lacZ*, *ey>dRas*^*V12*^*/mkk4-RNAi*, *ey>dRas*^*V12*^*/hts-RNAi*, *ey>dRas*^*V12*^*/pan-RNAi* (scale bars are 100μm). **(c)** *dRas*^*V12*^-driven eye growth enhancement by *RNAi* lines targeting 100 genes with top ranking TSOS scores. Enhanced rough eye phenotypes were scored on a scale ranging from 0–3, where a value of 0 indicates no and 3 shows maximum phenotype enhancement. At least two or more different hairpins were tested for 96 genes to confirm the *RNAi* effects, except for *Slu7*, *Synd*, *CG7378* and *l(2)k14710* (*CG8325*). Diamonds indicate scores for individual *RNAi* lines. Red line, mean of phenotype scores when multiple *RNAi* were tested.

We next selected the top one hundred candidate genes with the lowest expression scores in all human cancers assayed as compared to their respective normal tissue controls (Fig 3A, S4 Table). The respective fly orthologs (using several *RNAi* lines to each gene, where possible) were then retested in a secondary *Drosophila* screen using the *ey-GAL4 UAS-dRas*^*V12*^ system. In this system, expression of oncogenic *dRas*^*V12*^ in the eye epithelium results in a rough adult eye phenotype due to epithelial hyperplasia that can be scored for the effects of the *dRas*^*V12*^ cooperating genes [26]. Enhanced rough eye phenotypes were scored on a scale ranging from 0–3, where a value of 0 indicates no and 3 shows maximum phenotype enhancement. In these confirmation assays, downregulation of eighty out of the 100 retested genes resulted in increased hyperplasia of the eye epithelium, using an arbitrary cut off of an average score of 0.75 or above, specifically in the presence of constitutively activated *dRas*^*V12*^ (Fig 3B and 3C; S5 Table). It should be noted that although some of the *RNAi* lines tested were KK *RNAi* lines (indicated by KK in S5 Table), some of which may overexpress the *tiptop* (*tio*) gene and result in potentially aberrant effects [27], we have previously shown that *tio* overexpression does not modify the *ey>dRas*^*V12*^ phenotype [28]. Thus, the results from the KK lines tested, which mostly confirmed the results of GD lines, are valid. Interestingly, knockdown of *tio* was identified as cooperating with *dRas*^*V12*^ in our primary screen and was validated as a *dRas*^*V12*^ cooperating tumor suppressor in our secondary screen, consistent with its interaction with the Hippo pathway [27] and the known interaction of the Hippo pathway with Ras signaling [29]. Altogether, the results from our secondary screen indicate that eighty of the top 100 genes are evolutionarily conserved tumor suppressors that control epithelial tissue growth.

### A network map of *Ras*^*V12*^-driven tumorigenesis

To characterize the molecular networks collaborating with *Ras*^*V12*^-driven tumorigenesis we identified first degree binding partners for both the primary fly hits (S6 Table) and their conserved human orthologs (S7 Table). We only included binding partners if they interacted with at least two hits/orthologs. Gene Ontology (GO) analysis on the primary hits and their first degree binding partners revealed statistically significant enrichment of genes involved in cell adhesion, polarity, differentiation, vesicular transport, growth and apoptosis regulation in *Drosophila* (S6 Table). In humans, we observed enrichment of many biological processes linked with tumorigenesis including cell migration, morphogenesis, MAPK activation, mitochondrial functions, phosphorylation and signaling, growth, metabolism, lipid metabolism, and cell adhesion (S7 Table). Among known human tumor suppressor genes, we, for instance, identified *DCC*, *NF1*, *TSC2*, *FCLN*, as well as genes known to be involved in suppression of tumor cell motility and invasion, such as *Chordin*, *Stardust*, *TIMP2* and *Mdyn-D7* (encoding a nucleoside diphosphate kinase related to nm23/Awd, a metastasis tumor suppressor in both flies and mammals) [3, 30–33], or genes involved in cell polarity (e.g. *MDPZ/patj*, *MPP5/sdt*, *NCAM2/fas2*) [34, 35]. Other identified genes [e.g. *TMEM47* (*claudin* homolog), *MYH10/zip* (*MyoII*), *CTNNA1*, *A2 (*α*-Catenin*) *ROBO1-3*, or *VAV1*,*2*] have functions in cell adhesion, cell morphology, and have been implicated in the epithelial-to-mesenchymal transition (EMT) and invasion and metastasis [30, 36].

**Fig 4:**
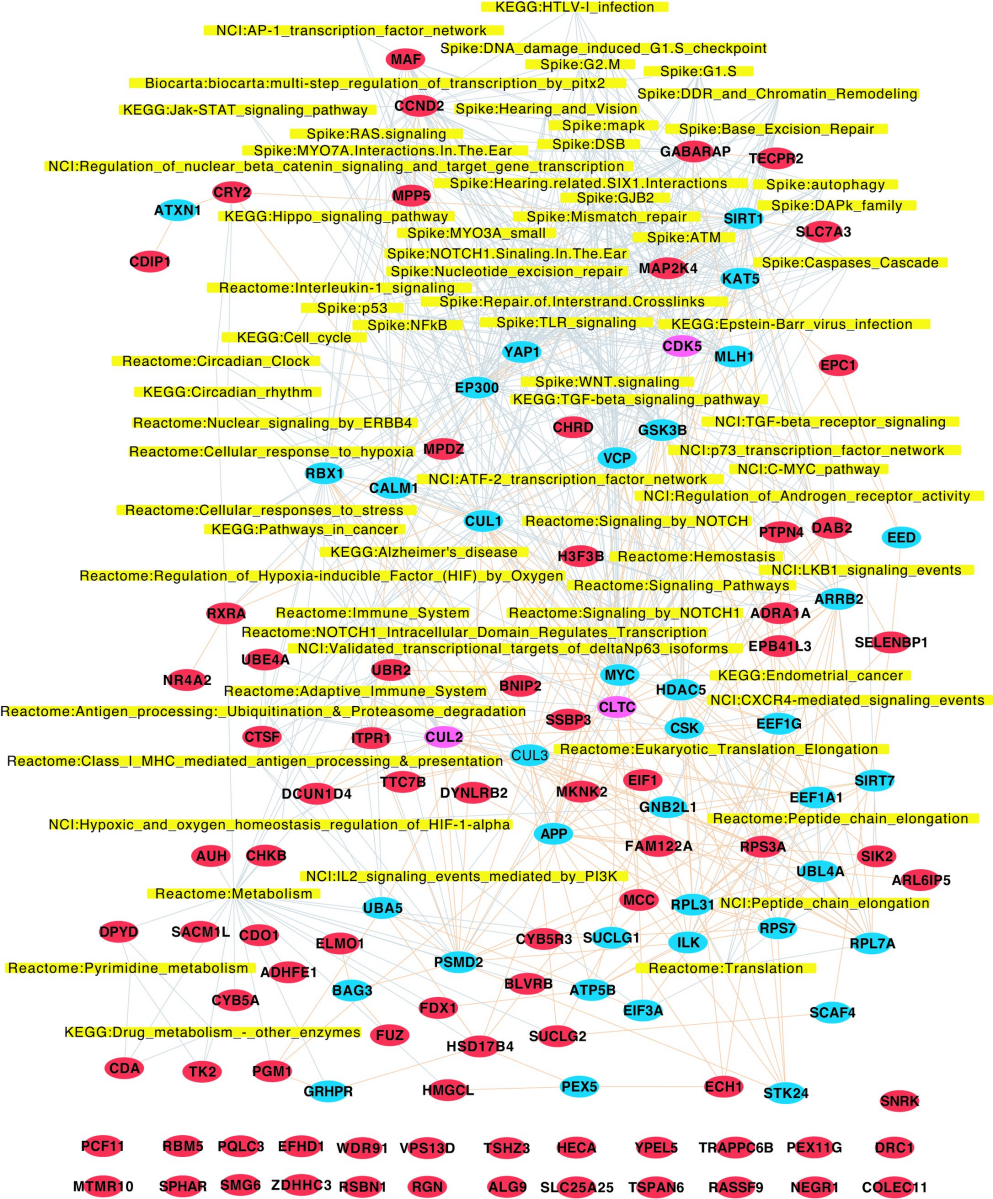
A conserved network map of oncogenic Ras-driven tumorigenesis identifies a novel sets of cancer genes. 70 significantly enriched terms of the graphite pathway gene set collection with nominal p-value < = 0.01 and terms with more than 2 candidate genes for the 80 confirmed tumor suppressor genes (red) with 40 BioGRID (v3.2.11) binding partners (blue and purple). Only first degree binding partners were included that interact with at least 2 direct hits and were present in the set of human orthologs from all screened *Drosophila* genes. The Systems Map describes 565 gene-to-term associations (grey) and 215 BioGrid protein interactions (light red) among the candidate genes and binding partners. The functional evaluation was performed by an enrichment analysis using a hypergeometric test for the gene sets of KEGGs, Reactome, NCI, Spike and Biocarta. The 25 genes that could not be assigned to pathways are shown in red at the bottom.

Enrichment analysis of KEGG pathways and MsigDB C2 gene sets [37, 38] on human orthologs and their first degree binding partners (S7 Table) identified MAPK signaling, Wnt, VEGF, TGFβ, or EGFR signaling pathways, all strongly implicated in cancer [39, 40]. Moreover, genes annotated to leukocyte transendothelial migration, cell adhesion, adherence or tight junctions, and, most importantly Ras signaling, were markedly enriched in our KEGG and C2 pathway maps (S3 Fig, S8 Table). Finally, we analysed the 80 human orthologs that were confirmed in our secondary *Drosophila* screen, along with their 40 first degree binding partners, which interacted directly with at least 2 of the 80 validated hits (S5 Table). Using this approach, 55 out of the 80 validated hits could be assigned to pathways associated with tumorigenesis and invasion, such as Ras, Wnt, Notch, or EGFR signaling, as well as DNA damage response and repair, cell cycle checkpoint control, immune responses, metabolism, autophagy or apoptosis (Fig 4, S9 Table). Further, we found that key nodes of regulation by our confirmed hits in this interaction map were known molecules that modulate survival, EMT and drug resistance in *Ras* mutated pancreatic cancers, such as YAP1 [41, 42], SIRT1 [43, 44], BAG3 [45, 46], ILK [47–49], CDK5 [50–52], HDACs or GSK3β [53, 54]. Overall, our functional screen in *Drosophila* has identified many known mammalian cancer genes. Importantly, 25 of the 80 validated hits could not be assigned to known signaling networks, indicating that this approach identified novel genes with putative tumor suppressor functions.

### Survival predictions in human pancreatic cancer patients

**Fig 5:**
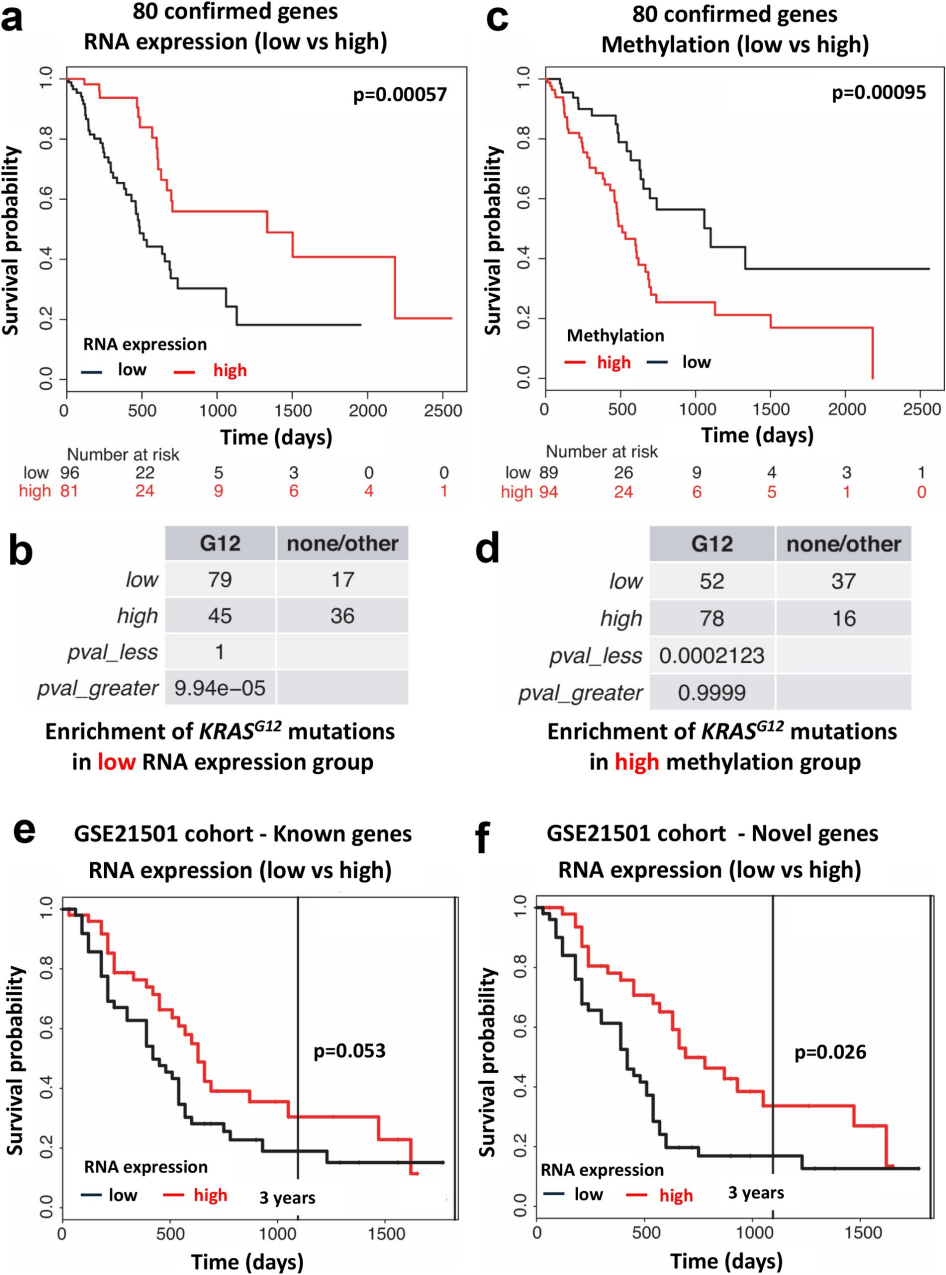
Expression and methylation of the validated cancer genes is associated the *KRAS*^*G12*^ status and predicts survival of pancreatic cancer patients. **(a)** mRNA expression of the human orthologs of the validated 80 cancer candidate genes is significantly associated with survival of pancreas cancer patients. Data were obtained using K-means clustering on the pancreatic adenocarcinoma TCGA patient cohort. P values (log rank test) and total numbers of patients with either low (black lines) or high (red lines) expression of the entire 80 candidate gene set are indicated. **(b)** mRNA expression levels of these 80 cancer genes are significantly associated with the *KRAS*^*G12*^ mutational status in human pancreas cancer (the same TCGA cohort as shown in (a) was analysed). The *KRAS^G12^* mutational status is shown for the low and high mRNA expression gene sets. None = no mutation; G12 = *KRAS*^*G12*^ mutation; other = mutations in *RAS* other than *KRAS^G12^*). **(c,d)** CpG methylation of the human orthologs of the validated 80 cancer candidate genes is significantly associated with **(c)** survival and **(d)** the *KRAS*^*G12*^ mutational status in human pancreas cancer. P values (log rank test) and total numbers of patients with either low (black lines) or high (red lines) methylation of the 80 candidate gene set are indicated. Data were obtained using K-means clustering on the pancreatic adenocarcinoma TCGA patient cohort. The *KRAS^G12^* mutational status is tabulated for the low and high methylation expression gene sets in (d). **(e)** mRNA Expression of the set of 55 known and **(f)** set of 25 novel gene sets is associated with survival of pancreatic cancer patients from the GSE21501 cohort. P values (log rank test) comparing the patient cohorts with either low (black lines) or high (red lines) mRNA expression of the 55 known and 25 novel gene sets are indicated. Pancreatic patient survival analysis was calculated using the ProgGeneV2 prognostic tool.

Our experimental approach aimed to identify evolutionarily conserved tumor suppressors of RAS codon 12 mutant-driven carcinomas. Based on the observed cooperative interaction, putative tumor suppressor genes identified in our screen should show low mRNA expression in human *KRAS*^*G12*^ mutated tumors. We first tested whether expression of the human orthologs of the 80 confirmed *Drosophila* hits correlate with the survival of patients with pancreatic adenocarcinomas, a tumor-type with frequent *KRAS* activating mutations. Tumor mRNA expression levels (stratified by high and low expression levels) for the 80 human gene orthologs showed that this gene set indeed predicts survival of pancreatic cancer patients (TCGA cohort), with low expression significantly correlating with poor survival (Fig 5A). In addition to poor survival, tumors exhibiting low mRNA expression of the 80 genes were markedly enriched for *KRAS*^*G12*^ mutations (Fig 5B). Analysis of the TCGA cohort of lung and colon adenocarcinomas, cancers in which *Ras*^*G12*^ mutations are known to be commonly involved, showed that whilst low expression of the 80 genes correlated with poor prognosis in lung cancer at high significance, this was not observed in colon cancer samples (Data File S1). Moreover, in lung and colon adenocarcinomas, there was no correlation with low expression of the 80 genes and the G12 activating mutation in *KRAS*, *HaRas* or *NRas* mutations, or with other Ras activating mutations, G13 or Q61 [55] (Data File S1).

Considering that we did not observe high frequency of genetic mutations for the 80 gene-set in these pancreatic tumors (CBioportal), we next asked whether these genes may be epigenetically regulated in *KRAS*^*G12*^ mutant tumors. In line with mRNA survival analysis, CpG methylation analysis of pancreatic adenocarcinoma tumors (TCGA) revealed that high methylation of the 80 genes in pancreatic tumors is significantly associated with worse overall survival (Fig 5C). Moreover, using enrichment analysis, high methylation was associated with the *KRAS*^*G12*^ mutational status (Fig 5D). Finally, we stratified our validated gene set into two groups: 1) the 55 known cancer genes and 2) the 25 novel, unassigned genes. Low expression of the set of 55 known cancer genes was associated with poorer patient survival in pancreatic cancer patients from the GSE21501 cohort, although this was just above statistical significance of p = 0.05 (Fig 5E). Importantly, low expression of the set of 25 novel unassigned genes showed a significant association with poor survival in these pancreatic cancer patients (Fig 5F). These results show that our screen has uncovered evolutionarily conserved cancer genes that, collectively, correlate with the *KRAS* mutation status of tumors and predict survival of patients diagnosed with pancreatic cancer.

### *Drosophila* Tsp29Fb affects *dRas*^*V12*^-driven tumor growth and metastases

**Fig 6:**
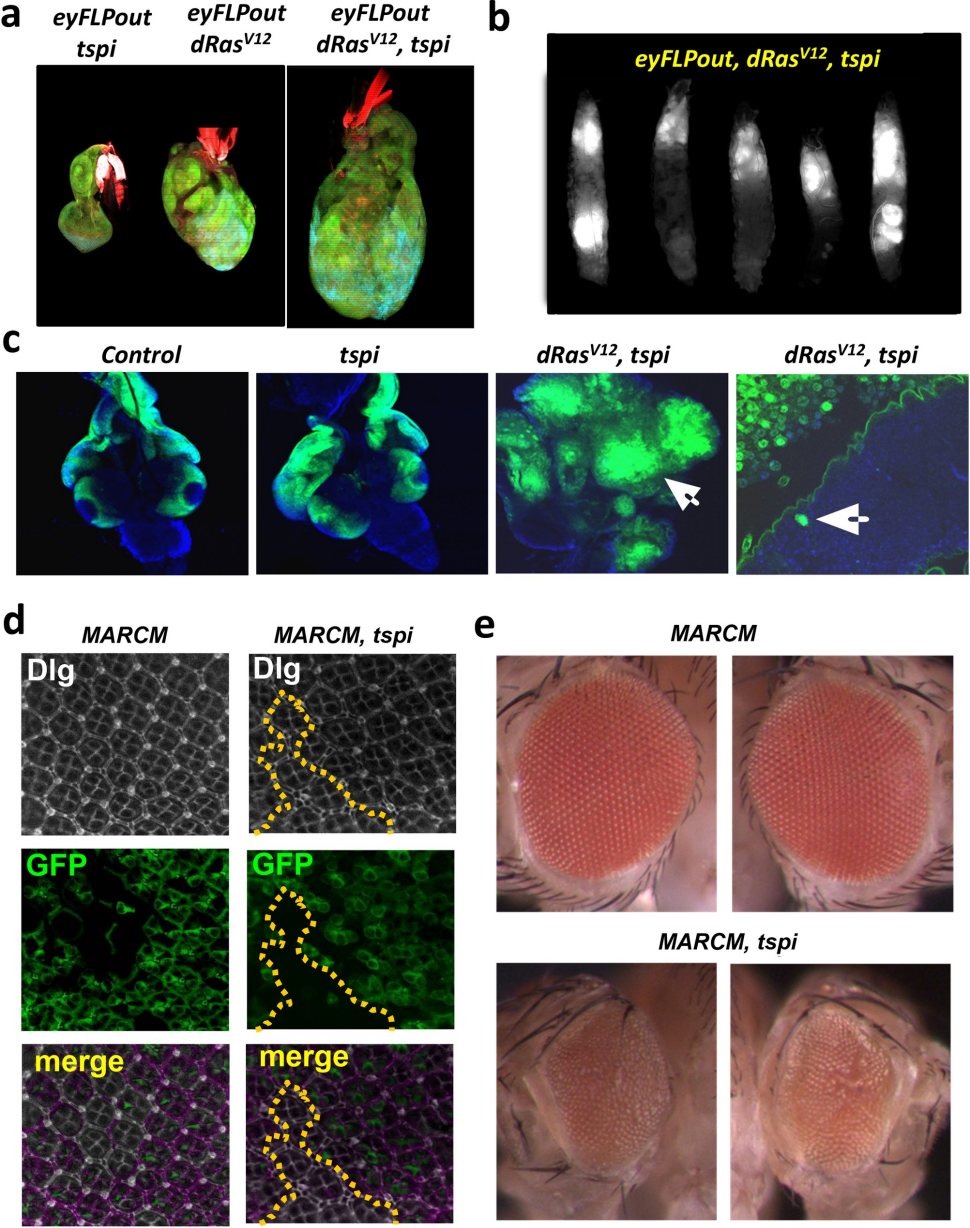
Knockdown of Tsp29Fb affects cell morphology and cooperates with dRas^V12^ in tumor growth and invasion. **(a)** Co-expression of *Tsp29FbRNAi (tspi)* with *dRas*^*V12*^ using the *ey-FLP-out* system results in neoplastic transformation and overgrowth of larval eye-antenna discs (right), whilst *tspi* expression shows no overt phenotype (left) and *dRas*^*V12*^ leads to hyperplastic larval discs without interfering with pupariation. Magnifications 20X. GFP (green) shows the ey-FLP-out tissue. The tissue is stained with Phallodin-TRITC (red) to detect F-actin. **(b)** *tspi dRas*^*V12*^ third instar larvae exhibit massive overgrowth and giant tumors. Magnifications 4X. **(c)** *ey-FLP-out tspi*, *dRas*^*V12*^ and *tspi dRas*^*V12*^ third instar larval eye discs propagated at 29°C, showing that *tspi dRas*^*V12*^ GFP+ cells have migrated into the VNC. Arrows show tissue disorganisation and tumor fusion to optic lobes (far right) with ventral nerve cord invasion (second from the right). **(d)** Pupal eye discs containing *MARCM* control or *tspi* clones (bottom, marked by the presence of GFP, and the clonal boundary is outlined by a yellow dotted line). Dlg is downregulated and mislocalized from the apical cortex upon *Tsp29Fb* knockdown. **(e)** Control *MARCM* adult eyes and *tspi MARCM* adult eyes. Tsp29Fb knockdown in clones results in a small rough eye phenotype.

Among the 25 novel hits, the strongest phenotypes in the secondary screen was observed for *Tsp29Fb*, a member of the Tetraspanin family coding for four-pass transmembrane proteins [56]. Therefore, we focused on the functional characterization of Tsp29Fb. During development, *Tsp29Fb* mRNA expression occurs in various larval tissues reaching the highest level at the third instar larval stage and in adults (http://flybase.org/reports/FBgn0032075.html). *ey-Flp*-mediated knockdown of Tsp29Fb (using a *RNAi* transgene (v2824), subsequently labelled *tspi* or *Tsp29Fb-RNAi* in the Figures), had no overt effect on untransformed eye discs (Fig 6A), but reduced adult eye and head size. However, in a *dRas*^*V12*^ background, *RNAi*-mediated knock-down of *Tsp29Fb* resulted in larval arrest and massive overgrowth with invasion of GFP-labeled tumor cells into the ventral nerve cord, ectopic foci formation, and invasion into the hemolymph (Fig 6A–6C). In the absence of *dRas*^*V12*^ expression, knockdown of *Tsp29Fb* driven by different eye or wing specific drivers with distinct temporal and tissue expression (*GMR-GAL4*, *en-GAL4* and *MS1096-GAL4*) resulted in reduced eye or wing sizes (S4A–S4D Fig), indicating that knockdown of *Tsp29Fb* itself can negatively affect tissue growth. Thus, Tsp29Fb knockdown results in reduced tissue growth, but in *dRas*^*V12*^ expressing tissue it enhances *dRas*^*V12*^-driven tumor growth and invasion.

### Tsp29Fb controls epithelial architecture and EGFR signaling

We next explored the molecular mechanism by which Tsp29Fb affects tissue growth using the power of fly genetics to specifically interfere with cell death pathways or defined signaling pathways. The eye and wing growth defects were not caused by changes in cell death, since no organ size rescue was observed when blocking apoptosis with Bsk^DN^ (kinase-dead JNK) or the caspase inhibitors DIAP1 and p35 (S4B Fig and S5A Fig), and no proliferation changes were detected in the larval epithelium by EdU and phosphorylation of Histone 3 (PH3) staining. However, knockdown of Tsp29Fb in the developing eye affected baso-lateral junction morphology with aberrant localization of the polarity protein Dlg and actin polymerization in the pupal retina (S5B Fig). Importantly, Dlg localization defects were also observed in *MARCM Tsp29Fb-RNAi* clones in the pupal retinal epithelium (GFP^+^) (Fig 6D), giving rise to smaller rough adult eyes (Fig 6E). Dlg localization appeared diffuse (fuzzy) at the junctions in the *MARCM Tsp29Fb-RNAi* clones relative to wild-type clones, whilst the baso-lateral junction cell polarity protein Scribbled (Scrib) and the adherens junction protein E-cadherin (E-Cad), abundance or localization were not affected (S6A Fig), suggesting that the Tsp29Fb specifically affects Dlg localization. Although Dlg protein junctional localization was abnormal in Tsp29Fb depleted tissue, Dlg protein abundance in the *Tsp29Fb-RNAi* clones was not significantly affected (S6A Fig, S10 Table), nor was Dlg protein abundance in *eyFLP-out* larval eye tissue as determined by Western blot quantification (S6B Fig, S11 Table). Thus, Tsp29Fb specifically affects Dlg junctional localization.

To assess whether the effect of Tsp29Fb knock-down on Dlg localization is functionally important, we examined genetic interactions with the cell polarity gene, *scrib*, which functions with *dlg* at baso-lateral junctions. We used a *UAS-scrib-RNAi* (previously shown to be effective in knocking down Scrib protein levels and specific for *scrib* [57]), expressed in the central region of the developing wing epithelium via *dpp*^*BLK*^*-GAL4* (S6C Fig), which resulted in a small wing phenotype with reduced area between wing veins L3 and L4, and breaks in the L3 wing vein. Co-knockdown of *Tsp29Fb* with *scrib*, strongly enhanced the loss of L3 vein defect (S6C Fig, S12 Table). Thus, Tsp29Fb affects Dlg junctional localization and genetically interacts with the Scrib/Dlg module.

**Fig 7:**
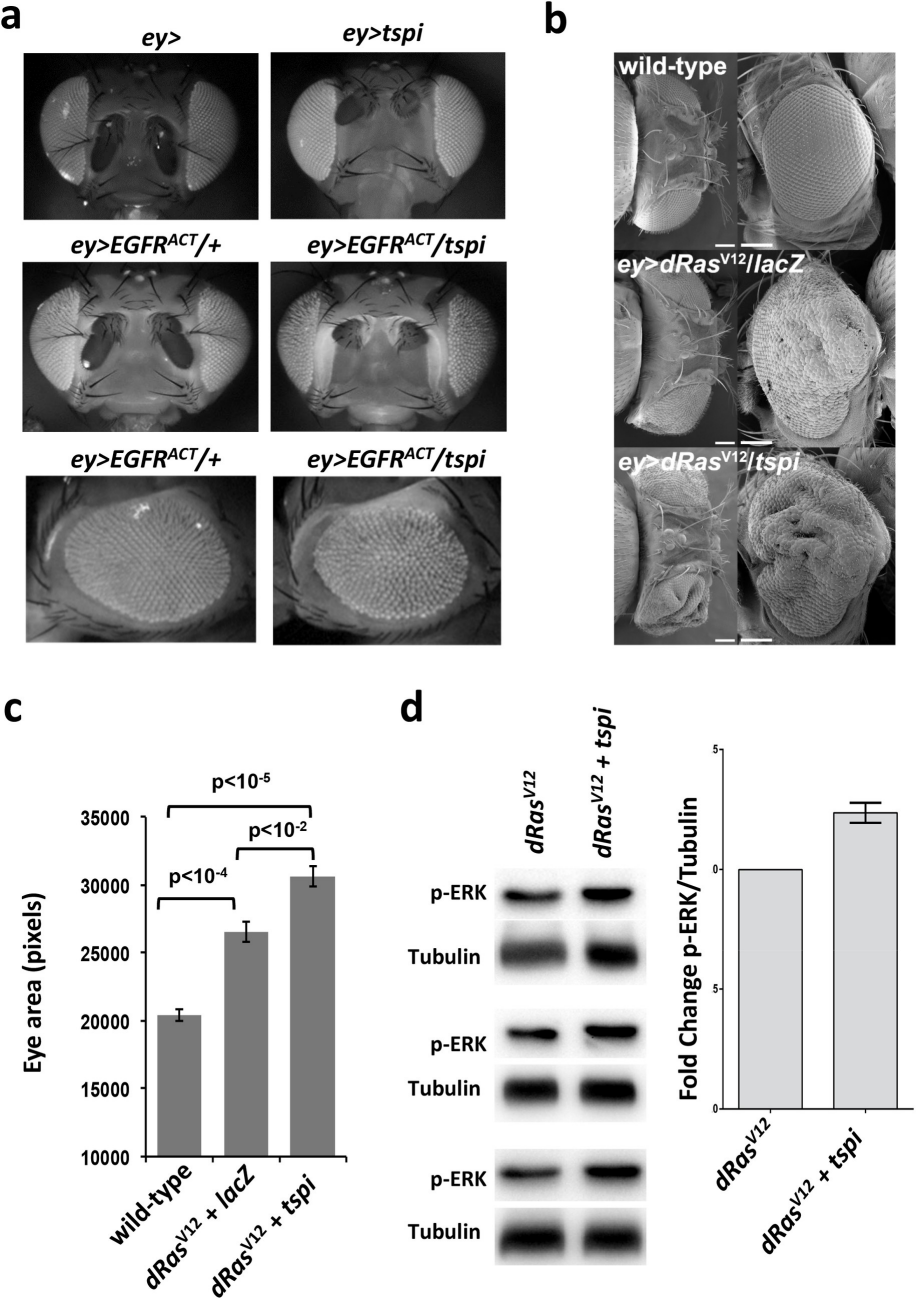
Tsp29Fb modulates EGFR/Ras activity in *Drosophila*. **(a)** *ey>tspi* enhances the reduced rough eye phenotype due to a *EGFR-ElpB1* dominant activating mutation *EGFR^ACT^* (middle panels front views, lower panels side views) relative to controls (top panels, front view, photographed on a CCD black-white camera). **(b,c)** Co-expression of *tspi* with *dRas^V12^* enhances the *dRas^V12^*-induced hyperplastic eye phenotype. **(b)** shows representative images. In **(c)** total eye areas were quantified for control wild-type *ey>* (n = 5), *ey>dRas^V12^*, *lacZ* (n = 6), and *ey>dRas^V12^*,*tspi* (n = 4) flies and are presented in the bar graph as mean values +/- SEM. Images made by scanning electron microscope. Flies propagated at 29 oC unless stated otherwise. **(d)** Western blots showing increased p-ERK levels in *ey-FLP-out tspi dRas^V12^* compared to *dRas^V12^* third instar larval eye-antennal discs at day 4 AEL. All crosses were propagated at 29°C. Results of three independent experiments are presented (left) with the mean value increase ± s.e.m. presented on the bar-graph.

We finally investigated which signaling pathway(s) could be modulated by *Tsp29Fb*. We failed to find any interactions or effects of *Tsp29Fb* down-regulation on Notch, Jak-Stat, JNK, Hippo, Wingless (Wnt) or Dpp (BMP/TGFβ) signaling as assessed by phenotypic modification or target gene expression (S5C Fig). However, *Tsp29Fb* knockdown in the eye showed a genetic interaction with a *Drosophila* constitutively active *EGFR* allele [58] (Fig 7A). In addition to functional interaction with fly *EGFR*, we again independently confirmed that simultaneous down-regulation of *Tsp29Fb* (using v2824) with *dRas*^*V12*^ expression via *ey* resulted in an enhanced overgrowth eye phenotype (Fig 7B and 7C, S13 Table). A second *Tsp29Fb RNAi* line (v2823) also showed enhancement of the *dRas*^*V12*^-driven eye tissue overgrowth (S7A Fig). Conversely, overexpression of *Tsp29Fb*, using the *UAS-Tsp29Fb-HA* transgene, showed suppression of the *ey>dRas*^*V12*^ hyperplastic phenotype (S7B Fig, S14 Table), consistent with Tsp29Fb acting to reduce dRas signaling. As expected from a functional association, knocking down *Tsp29Fb* expression in *dRas*^*V12*^ transformed eye-antenna discs enhanced phosphorylation of key EGFR-Ras-downstream target ERK (pERK) (Fig 7D, S15 Table). Tsp29Fb knockdown alone also led to increased pERK without effecting total ERK relative to the control (S7C Fig, S16 Table). These genetic and biochemical results in *Drosophila* indicate that knock-down of Tsp29Fb affects epithelial architecture and enhances EGFR-Ras signaling.

## Discussion

We have performed a large-scale screen in *Drosophila melanogaster* to identify genes involved in suppression of *Ras*^*V12*^-induced epithelial tumors and tested 10,777 *UAS-RNAi* lines corresponding to evolutionarily conserved 6675 genes. For this screen, we expressed *dRas*^*V12*^ and *UAS-RNAi* constructs in the eye imaginal disc of fly larvae, which allowed us to monitor tumor formation, invasion, and formation of distant site “metastases”. Moreover, *RNAi*-mediated knockdown allowed us to assay a large number of previously unexplored genes in epithelial tumorigenesis *in vivo*. Using this experimental set-up, we identified multiple known tumor suppressor pathways as well as many novel genes and pathways potentially involved in the suppression of *Ras*^*V12*^-driven epithelial tumorigenesis. Importantly, human genes orthologous to the primary fly hits that, as an entire data set, were massively downregulated when we assayed gene expression in thousands of human cancers, supporting the concept that genes controlling epithelial growth, fate and integrity are highly conserved across phyla. The top 100 candidate genes stratified in human cancers were then further validated in secondary *Drosophila* Ras-driven eye hyperplasia assays, confirming that 80 of these genes were cooperating tumor suppressors with *dRas*^*V12*^ in epithelial tissues. This screen allowed us to construct an evolutionarily conserved network of genes and pathways predicted to cooperate with Ras in tumor growth and invasion-metastasis. This data set should provide a starting point for the identification of novel genes and pathways involved in tumor suppression and metastasis of Ras-driven epithelial cancers.

Loss of function screens have previously been performed in *Drosophila*, identifying genes that modify Ras function [59, 60]. However, only 20–30% of genes in *Drosophila* genome can be mutated to give a detectable phenotype [61], leaving the function of a majority of expressed gene unexplored. In addition, these screens have been largely conducted in differentiating eye cells of the *Drosophila* larvae/pupae that display limited or no mitotic potential and undergo final steps of differentiation [60]. Moreover, in previous studies, *Drosophila* has been established as a tool to screen for invasive tumor growth [14, 15]; but these screens relied on genetic mutants and required 3 crosses to analyze the effects due to homozygous lethality of the mutants and pupal stage lethality of *dRas*^*V12*^ expression alone. We therefore established a model that circumvents *dRas*^*V12*^-induced lethality by using a *CyO*, *tub-GAL80* balancer [62], offering a system that can be used for rapid screening and to model hyperplastic tumor growth, and formation of distant site “metastases” in a pool of epithelial progenitor cells. Using this experimental system, we tested genes that are conserved between *Drosophila* and human using *RNAi*-mediated knockdown, which allowed us to assay the role of a large number of previously unexplored genes in Ras-driven epithelial tumorigenesis *in vivo*. Since hallmarks of cancer such as migration and proliferation are regulated by evolutionarily conserved pathways, e.g. Ras/MAPK, Wnt or Hedgehog signaling, our high primary hit rate can be explained by the selected set of conserved genes [63]. C2 and KEGG analysis of our hit list revealed that our screen had markedly enriched for known genes and pathways implicated in mammalian tumorigenesis including cell adhesion, cell polarity, phosphoinositide signaling, metabolism, or Wnt, Hedgehog, and, as one must expect, Ras signaling.

Interestingly, several genes and pathways identified in our primary screen have an effect on oxidative stress levels or proper mitochondrial function: detoxifying enzymes such as Gutathione S Transferases or Wwox [64], and many mitochondrial genes, the loss of which can potentially impede proper mitochondrial function and generate oxidative stress. Interestingly, we have recently shown that increased oxidative stress levels induced by autophagy impairment can indeed cooperate with oncogenic Ras by triggering the JNK stress response pathway [28].

Perhaps not surprisingly, giving the central role of Ras in conveying intracellular signals, we find that oncogenic Ras cooperates with a wide range of cellular processes scattered across all the different cellular compartments. However, we can attempt to group the Ras-cooperating tumor suppressors identified and validated in our screen into a few pivotal mechanisms that influence Ras tumorigenic outcome: i) modification of the DNA structure, replication or regulation (eg chromatin remodelling, cell cycle regulators, DNA repair enzymes); ii) regulation of Ras and other signalling pathways (eg Wnt, Notch, Hippo, Jak/Stat, TGFβ, PI3K); iii) regulation of apoptosis; iv) disruption of metabolic homeostasis and increase of oxidative stress (eg glycogen breakdown, glucose transport, TCA cycle, autophagy); iv) disruption of plasma membrane organisation or homeostasis (eg cell junction organization, endocytosis). Indeed in this study, in line with recent data suggesting that Tetraspanins are enriched in membrane micro-domains and regulate receptor signaling [65–67], we found that *Tsp29Fb* genetically interacts with *EGFR* and *dRas* and regulates EGFR-Ras signaling. Whilst we have concentrated here on the validated top 80 genes, future analysis of the remaining hits from our screen, may reveal new signaling pathways, or reveal further novel tumor suppressors, involved in Ras-driven tumorigenesis.

Of the 80 confirmed genes, 55 belonged to known signaling networks, whereas 25 could not be assigned to any known functional module. Expression as well as methylation analysis of these genes accurately stratified high and low risk survival groups of patients diagnosed with pancreatic cancer, and also correlated with the mutational status for *RAS*^*G12*^ in the pancreas tumors of these patients. Given our findings, it will be important to assess whether low expression of the 80 confirmed genes is correlated with poor patient survival in other cancers where RAS signaling is elevated, such as EGFR or RAS-GAP (NF1) tumors.

The top hit among the novel 25 tumor suppressor genes was the Tetraspanin family member, *Tsp29Fb*. Mechanistically, our genetic and biochemical experiments show that *Drosophila* Tsp29Fb modulates Ras activation and in genetic epistasis experiments affects EGFR-Ras signaling. Whether Tsp29Fb regulates other signaling pathways, alone or together with *dRas*^*V12*^, needs to be further explored. Interestingly, depletion of Tsp29Fb resulted in mislocalized Dlg at cell junctions, and ommatidial patterning defects in terminally-differentiating photoreceptor cells, implicating this protein in epithelial apico-basal polarity regulation and the control of epithelial architecture. It is possible that these effects of Tsp29Fb depletion on epithelial architecture directly affect EGFR/Ras activation, as Scrib depletion does in mammalian cells [68, 69] and in *Drosophila* epithelial tissue [70]. Indeed, our results showing that *Tsp29Fb* genetically interacts with *scrib* support that notion that Tsp29Fb regulates, or functions with, Scrib/Dlg in cell polarity regulation in epithelial development and in the control of signaling pathways. Further analysis of how *Tsp29Fb*, as well as the other novel tumor suppressor genes identified in our screen, cooperate with *dRas*^*V12*^ in tumorigenesis, will potentially reveal the importance of cell polarity regulation, as well as other novel pathways, in Ras-driven cancer.

## Materials and methods

### Fly stocks

All *RNAi* transgenic fly lines used in the primary screen were obtained from the VDRC *RNAi* stocks [71]. The following lines were used for generating the driver line for the screen: the *P(UAS-2xEGFP), P(ey3.1-FLP)1, y(1) w(1118)* and *P(Act5C(FRT.y[+])GAL4)2* insertion lines and *UAS-scrib-RNAi C2V* (VDRC 11663, 2^nd^ Chromosome, viable) were obtained from Barry Dickson (IMP, Vienna). The fly stocks carrying insertion *P (UAS-Ras85DV12)TL1 and P (UAS-Ras85DN17)TL1* were obtained from the Bloomington *Drosophila* Stock Centre (BDSC) (BL4847 and BL4846, respectively). The *CyO*, *P* (*Tub-Gal80*) line was a kind gift from Olaf Vef, Institute for Genetics, Mainz [62], *UAS-Raf*^*DN*^ was a kind gift from C. Samakovlis, Wenner-Gren Institute, Stockholm [72]. *w*^*1118*^ wild type control flies were obtained from the VDRC and used for controls since the *Drosophila melanogaster w*^*1118*^ background is isogenic to the VDRC *RNAi* library. Other fly stocks were: *UAS-Dicer2* (VDRC; 60009), *UAS-luciferase* (BL31603, BDRC), *ey-GAL4* (J. Treisman [73, 74]), *GMR-GAL4* (B. Hay; [75]), *MS1096-GAL4* (BDRC; BL8860), *en-GAL4 UAS-GFP* (L. Johnston), *C96-GAL4* (BDRC; BL43343), *UAS-lacZ* (G. Baeg), *UAS-bsk*^*DN*^ (M. Mlodzik [76]), *UAS-Diap1* (BDRC; BL6657), *UAS-p35* (B. Hay [77]), *UAS-mamH* (BL26672) and *EGFR-ElpB1/CyO* (*EGFR*^*E3*^; [58])), *ey>dRas*^*V12*^ [26], *ey-FLP; Actin>CD2>GAL4*, *UAS-GFP*/*TM6B* or *Actin>CD2>GAL4*, *UAS-RFP*/*TM6B* (*ey-FLP-out*, this study), *UAS-GFP; dpp*^*BLK*^*-GAL4 Tub-GAL80*^*ts*^*/TM6B* (this study), *UAS-scrib-RNAi UAS-GFP; dpp*^*BLK*^*-GAL4 Tub-GAL80*^*ts*^*/TM6B* (this study), *ey-FLP1*,*UAS-mCD8-GFP; Tub-GAL4*,*FRT82B*, *Tub-GAL80* (*MARCM3*, J. Treisman), *UAS-Tsp29Fb-HA* (NCBS *Drosophila* Facility, Bangalore), and the *Tsp29Fb* deficiency stock was a kind gift from R. Le Borgne (Université de Rennes, Rennes, France).

### Screening assay

Transgenic *UAS-RNAi* males were crossed to driver virgin females and the GFP positive progeny were analyzed at the indicated days after egg laying. The numbers of progeny arrested at the larval stage were counted. A Z score cut-off of 1.65 (Mean control-test)/SD was used to select for positive *RNAi* hits. Larval mouth hook invasion and occurrences of GFP-labelled foci at distinct sites were determined using a Leica fluorescence stereomicroscope.

### Secondary screening systems

*eyeless-GAL4*,*UAS-dRas*^*V12*^*/CyO* (*ey>Ras*^*V12*^) female flies were crossed to either *UAS-lacZ* males for control or to *UAS-RNAi* males. Crosses were raised at 29°C for 11 days before scoring. Enhancement of *Ras*^*V12*^-driven overgrowth was assessed visually in at least 20 F1 *ey-GAL4*,*UAS-dRas*^*V12*^*/UAS-RNAi* females by comparing them to *ey-GAL4*,*UAS-dRas*^*V12*^*/UAS-lacZ* females. Overgrowth was scored as: 0 = no enhancement, 1 = enhancement, 2 = strong enhancement, 3 = very strong enhancement. Intermediate values are representative of variable penetrance in the phenotype. Scores of each *RNAi* line tested were plotted on a graph. When several independent *RNAi* lines were scored for a particular gene, the mean was calculated and plotted on the graph as a red line. An *RNAi* line was considered validated when it showed enhancement of 1 or above.

### Immunohistochemistry of *Drosophila* larval cephalic complexes wing discs, and larval or pupal eye discs

For the analysis of cephalic complexes, eye/antennal and wing discs, larvae were picked at the third instar stage and appropriate age. Dissection was performed by pulling out larval mouth-hooks. Tissues were fixed in 3.7% buffered formalin solution for 20 min, blocked with 5% Normal Goat Serum in 1XPBST (0,1% Tween-20 in phosphate buffer saline) for 1hour at room temperature. Primary antibodies used were anti-Dlg (Mouse monoclonal 4F3, 1:50, Developmental Studies Hydridoma Bank (DSHB)), anti-Elav (Mouse monoclonal 1:50, DSHB), anti-E-Cad (Rat monoclonal, 1:50, DSHB), anti-Scrib (Rabbit polyclonal, 1:1000, C. Doe), anti-HA (rat 1:100, Roche). Secondary antibodies were anti-mouse Alexa 488, 568, 633, or anti-rat Alexa 488, 568 (Molecular probes). F-actin was detected using phalloidin–tetramethylrhodamine isothioblueate (TRITC; Sigma, 0.3 μM, 1/1000), and DNA using 2-(4-amidinophenyl)-1H-indole-6-carboxamidine (DAPI, 1mM). The samples were mounted with Vectashield DAPI containing medium (H-1200, Vector Laboratories, Inc., 30 Ingold Road, Burlingame, CA or in 80% (v/v) glycerol/PBS. Images were taken using Carl Zeiss LSM 410 UV, Bio-Rad MRC1000, Olympus FV1000, LEICA TCS SP5 or Zeiss LSM780 PicoQuant FLIM confocal laser scanning microscopes. Images were processed using Confocal AssistantR, Fluorview, Leica LAS AF Lite, Zeiss Zen or Image J software. Images were assembled using Adobe Photoshop CS5.1. Adult eyes were imaged with a Scitec Infinity1 camera.

For quantification of Dlg staining in pupal mosaic epithelial tissue, maximum intensity projection images were generated from a subset of stacks from each z-stack image. Large areas of GFP-positive or GFP-negative tissue were, roughly equal in size and adjacent to one another, were selected. Using the histogram tool in Adobe Photoshop CC, the mean pixel intensity for Dlg1 staining was determined for each GFP-positive and GFP-negative area, and the ratio between the two areas was determined. Statistical analyses were performed using GraphPad Prism 7.

For imaging of *Drosophila* adult eyes and wings, adult flies were frozen at -20°C before imaging in order to facilitate positioning them under the microscope. Images were captured on a Lumenera or Scitec Infinity1 camera attached to Olympus SZX7 dissection microscope and processed using Adobe Photoshop CS3. Flies were prepared for scanning electron microscopy by progressive dehydration in concentrations of ethanol ranging from 25% to 100% over the course of 4 days on a nutator. Flies were then desiccated by critical point drying in a Leica critical point dryer, mounted on steel stubs and coated with 20nm of gold particles in a sputter coater. Representative images of each phenotype were taken on a JEOL JCM-6000 NeoScope scanning electron microscope at 80x and 300x magnifications. Images were cropped and aligned in Adobe Photoshop. The area of adult eyes was measured in Photoshop CS5.1 and statistically analyzed using Microsoft Excel and GraphPad Prism. *Drosophila* wings were prepared from female flies and mounted in Canadian Balsam. Wing size was measured in Photoshop CS5.1 and statistically analyzed using Microsoft Excel and GraphPad Prism.

For quantification of the wing vein defects in the *dpp>scrib-RNAi* experiment, the total length of the L3 vein was measured in all samples using ImageJ, with a straight line drawn between any gaps (covering the shortest possible distance). The length of the gaps in the L3 vein were measured using the shortest possible distance between vein sections. The length of L3 vein loss to total L3 vein length was then expressed as a ratio, and analysed statistically using a Student’s t-test.

### Sample preparation and Western blot analysis of *Drosophila* eye-antennal discs

Eye-antennal discs (from 11 larvae per sample) were dissected from the *ey-FLP-out dRas*^*V12*^,*ey-FLP-out dRas*^*V12*^ *tspi*, *ey-FLP-out lacZ*, or *ey-FLP-out tspi* larvae, homogenized in 0.1 M Tris-HCl pH 6.8, 2% SDS, 5 mM EDTA, 5 mM DTT buffer containing Complete protease inhibitor cocktail (Roche, Basel, Switzerland), 1 mM Na3(VO)4, 5 mM NaF2. Protein concentration was determined by Lowry or DC assays. Samples containing 20 mg of protein were electrophoresed on a 10% SDS-PAGE gel and transferred to Immobilon-FL polyvinylidene difluoride membranes. Antibodies used were monoclonal mouse anti-pERK (diphosphorylated ERK1/2/MAPK1/2, 1:10.000, #M8159 Sigma), anti-ERK (rabbit polyclonal, 1:1000), anti-Dlg (mouse monoclonal 4F3, DHSB, 1:1000), and monoclonal mouse anti-α-Tubulin (1:10,000 Cell Biochem). Western band intensity was measured using ImageJ or Adobe Photoshop, and then normalized to the intensity value of the α-tubulin control using Microsoft Excel and statistical analyses were carried out using GraphPad Prism.

### Identification of mouse and human orthologs

To identify orthologs between *Drosophila* and mouse and between *Drosophila* and human, we obtained pre-computed one-to-one and one-to-many orthology predictions from Inparanoid (version 6.1) to Ensembl databases [78]. In case of one-to-many mappings, all the ortholog targets for a given gene were considered for downstream analysis.

### Expression analysis of human orthologs and TSOS calculations

3351 samples of tumor and normal control tissues were hybridized using an Affymetrix HG-U133ab chipset. If a defined gene showed a significantly higher expression in the tumor tissue compared to normal tissue (t-test, different variances, p-value < 0.01), it was scored +1. If the gene is significantly higher expressed in normal tissue, it was assigned a score of -1. If one gene has more than one probe set on the Affymetrix chip, the score was divided by the number of probe sets for this gene, e.g. if 2 of 3 probe sets of gene X showed a significantly higher expression in normal tissue and no significant differences in the 3rd probe set, expression was scored -2/3 for gene X in this specific tumor/normal tissue pair. The human orthologs of our putative tumor suppressor gene list were used to calculate the tumor-suppressor-oncogene-score (TSOS). For 619 of the 637 genes we found at least one probe set on our Affymetrix chip. Moreover, we assessed the TSOS scores using TCGA and GTEx RNAseq datasets. The TSOS is the sum of the described score for all genes in our list and all tumor/normal tissue pairs divided by the number of genes. It is a measure for an overall significantly higher expression in tumor tissue (positive values) or normal tissue (negative values). As a control, we randomly sampled 613 human genes from the *Drosophila* ortholog gene list one million times. Only 7 random lists had a lower TSOS than our gene list, which indicate a significantly low TSOS (p-value 7x10-6) for our gene list.

The following normal tissue/tumor tissue pairs were analyzed using arrays:

1. "BladderNormal-BladderTransCellCa"
2. "BreastGigantomastia-BreastInfDucCa"
3. "BreastGigantomastia-BreastInfLobCa"
4. "BreastNormal-BreastGigantomastia"
5. "BreastNormal-BreastInfDucCa"
6. "BreastNormal-BreastInfLobCa"
7. "BreastNormalCancerPatients-BreastInfDucCa"
8. "BreastNormalCancerPatients-BreastInfLobCa"
9. "BreastNormalEpithel.LCM-BreastMalignantEpithel.LCM"
10. "CerebellumNormal-BrainGlioblastMultiform"
11. "CervixNormal-CervixSquamCellCa"
12. "ColonNormal-ColonAdenoCa"
13. "ColonNormal-ColonMucinCa"
14. "ColonNormalEpithelCells.LCM-ColonMalignantEpithel.LCM"
15. "ColonNormalEpithelCells.LCM-ColonUlcerativeColEpithel.LCM"
16. "EsophagusNormal-EsophagusAdenoCa"
17. "KidneyNormal-KidneyClearCellCa"
18. "KidneyNormal-KidneyRenalCellCa"
19. "KidneyNormal-KidneyWilmsTumor"
20. "LarynxNormal-LarynxSquamCellCa"
21. "LiverNormal-LiverHepatoCellCa"
22. "LungNormal-LungAdenoCa"
23. "LungNormal-LungAdenosquamousCa_PV"
24. "LungNormal-LungLargeCellCa"
25. "LungNormal-LungSmallCellLungCa"
26. "LungNormal-LungSquamCellCa"
27. "LungNormalEpithel.LCM-LungAdenoEpithel.LCM"
28. "LungNormalEpithel.LCM-LungMalignantEpithel.LCM"
29. "LungNormalEpithel.LCM-LungSCCEpithel.LCM"
30. "LymphNodeNormal-LymphNodeAdenoCaMet"
31. "LymphNodeNormal-LymphNodeHodgkinDisease"
32. "LymphNodeNormal-LymphNodeInfDuctMet"
33. "LymphNodeNormal-LymphNodeMaligMelanoMet"
34. "LymphNodeNormal-LymphNodeNonHodgkinDisease"
35. "LymphNodeNormal-LymphNodeSquamCellCaMet"
36. "LymphocyteNormal-AllFollicularLymphoma"
37. "LymphocyteNormal-AllLargeBCellLymphoma"
38. "LymphocyteNormal-AllMantleCellLymphoma"
39. "LymphocyteNormal-AllMycosisFungoides"
40. "OvaryNormal-OvaryCa"
41. "PancreasNormal-PancreasAdenoCa"
42. "PancreasNormal-PancreasIsletCellCa"
43. "ProstateNormal-ProstateAdenoCa"
44. "ProstateNormalEpithel.LCM-ProstateBenHypertrEpithel.LCM"
45. "ProstateNormalEpithel.LCM-ProstateMalignantEpithel.LCM"
46. "SkinNormal-MelanocytesMaligMelanoma"
47. "SoftTissNormal-SoftTissMaligFibrousHistiocyt"
48. "SoftTissNormal-SoftTissOsteosarcoma"
49. "SoftTissNormal-SoftTissSarcoma"
50. "SoftTissNormal-SoftTissueNormalMalign"
51. "SpinalCordNormal-BrainGlioblastMultiform"
52. "SpleenNormal-AllFollicularLymphoma"
53. "SpleenNormal-AllLargeBCellLymphoma"
54. "SpleenNormal-AllMantleCellLymphoma"
55. "StomachNormal-StomachAdenoCa"
56. "ThymusNormal-AllMycosisFungoides"
57. "ThyroidGlandNormal-ThyroidGlandPapillCa"
58. "ThyroidGlandNormal-ThyroidGlandPapillFollicCa"

### Bioinformatics analysis of *Drosophila* hits and corresponding human orthologs

We performed a functional analysis separately for the *Drosophila* hits and their corresponding human orthologs for Gene Ontology, KEGG and C2 gene sets from the MsigDB database. In order to identify indirectly associated gene sets and pathways, we performed additional analysis considering also direct binding partners of the *Drosophila* hits and corresponding human orthologs. We defined protein interactions from the protein-protein interaction database BioGRID (version 2.0.50) in order to extract binding partners to the *Drosophila* candidate gene set and to the corresponding human orthologs. Only first degree binding partners that interact with at least two direct *Drosophila* hits or their human seed and inparalog orthologs were considered for the analysis.

Gene Ontology (GO) analysis was performed using the Biomart database [79] for *Drosophila melanogaster* and Ensembl (http://www.ensembl.org/) for *Homo sapiens*. For the Gene Ontology enrichment analysis we used the”topGO” package [80] from Bioconductor [81] version R-2.7.1. GO analysis was performed with fly hits and human orthologs whose *RNAi* hits had a Z-score >1.65. For each Gene Ontology term of the class”biological process”, "cellular component", and "molecular function" a hypergeometric test (one-sided Fisher exact test) was performed. A GO enrichment analysis was also performed for the *Drosophila* candidate gene set with and without binding partners and for human seed and inparalog orthologs with and without binding partners, where the reference gene set is defined by all screened *Drosophila* genes and for the corresponding human seed and inparalog orthologs. GO terms with a nominal p-value ≤ 0.05 were selected as significantly enriched. Since terms that occur at a deeper level in the GO tree hierarchy, therefore containing lesser numbers of genes, are considered more biologically informative, we discarded terms containing more than 500 genes from further analysis.

KEGG (Kyoto Encyclopedia of Genes and Genomes) annotation of genes for *Drosophila melanogaster* and *Homo sapiens* were retrieved from the KEGG database (http://www.genome.jp/kegg/). The C2 gene set assignments to human genes were obtained from the Molecular Signature Database (http://www.broadinstitute.org/gsea/msigdb). The C2 dataset defines a collection of curated gene sets derived from KEGG, BioCarta (http://www.biocarta.com/), GenMAPP (http://www.genmapp.org/), chemical and genetic perturbations gene sets and canonical pathway gene sets. All screened *Drosophila* genes and the corresponding human seed or inparalog orthologs were used as reference gene sets. A hypergeometric test (one-sided Fisher's Exact Test), similar to the test used for GO enrichment analysis, was used to identify over-represented gene lists (C2) and pathways (KEGG) amongst the hits. This analysis was performed on the gene list identified as human orthologs corresponding to the *Drosophila* cancer hits (Z-score > 1.65).

### Gene Ontology—GO—Graph visualization

Significantly enriched terms were arranged in a GO graph structure that was extracted using the bioconductor packages GOstat and GO.db. We implemented a procedure in R that creates a condensed GO graph structure for the set of significant terms by removing iteratively all non-significant parental terms from the graph. The child terms of non-significant parental terms that were removed were reconnected by an edge to the corresponding grandparental terms. The resulting graph layout was performed in Cytoscape (http://www.cytoscape.org/). The GO terms were subsequently manually assigned to "functional groups" based on their shared roles in a "biological process", "cellular component", "molecular function", for visual representation of the GO data.

### Generation of a systems map for the 80 confirmed highest scoring genes

We associated a total of 40 proteins that directly interact with at least two of the 80 candidate genes from experimental protein interactions defined from BioGrid (version 3.2.11) present in the set of human orthologs from all screened *Drosophila* genes. The analysis of the 80 selected candidate genes and their direct interactors was performed by an enrichment analysis using a hypergeometric test separately for the MsigDB database (v4.0), Gene Ontology gene sets (org.Hs.eg.db, Bioconductor package: Biological Process, Molecular Function, Cellular Component), Consensus pathway database (cpdb, http://consensuspathdb.org/), Pathway Commons (pcom, http://www.pathwaycommons.org/), gene sets from the graphite Bioconductor package including KEGG, Biocarta, NCI, Spike and Reactome. The MsigDB database (v4.0) includes gene sets for positional gene sets, curated gene sets (pathways), motif gene sets, computational gene sets, gene ontology (GO) gene sets, oncogenic signatures gene sets, and immunologic signatures gene sets (http://www.broadinstitute.org/gsea/msigdb).

### Human patient data survival analysis

We retrieved the RNAseq, methylation, *KRAS* mutation profiles and clinical data on patient survival from the TCGA database (version 2016_01_28, https://gdac.broadinstitute.org/). For mutational profiles we used MutSig [82] variant calls (TP.MutSigNozzleReport2.0.Level_4.2016012800). For the RNAseq and methylation profile survival analysis we considered only patient samples that were present in the MutSig report "TP.final_analysis_set.maf" and had clinical overall survival information. Using these criteria, for PAAD, 132/177 (74%) RNAseq patient samples with KRAS mutations were available; retrieving normalized illumina hiseq rnaseqv2 RSEM Level 3 data, the Clinical Level_1 and the methylation profiles of preprocessed mean aggregated gene level methylation beta values (Methylation_Preprocess.Level_3.2016012800, meth.by_mean.data). Patient samples were stratified in *RAS-G12* mutation carriers ("G12"), "other" for samples with KRAS mutations assigned but not *G12* mutations, and "none" for no *RAS* mutations. For the enrichments, the category “none” and “other” were aggregated. For survival analysis, patients were split into two groups using k-means clustering. The patient cohort with a larger average RNAseq expression or methylation beta values were denoted as "high" and the cohorts with lower average expression as "low". The k-means clustering procedure was repeated 100 times on all samples and a patient sample was designated to the most frequently assigned group (low or high). For the clustering procedure we log_e(1+x) transformed the normalized RSEM gene RNAseq samples and scaled the data (z-transformed and centered) for each patient sample. Enrichment and underrepresentation of *KRAS-G12* mutations in the low group compared to the high group was assessed using a one-sided Fisher’s exact test. The processing and analysis of the data was performed in R. For the survival analysis we used the survplot function of the survival R package (http://www.cbs.dtu.dk/~eklund/survplot/).

Survival analysis using PROGgeneV2 web-based tool was conducted using the following genes represented by probes on the respective arrays:

Set of known cancer genes: ADHFE1, ARL6IP5, AUH, BLVRB, BNIP2, CCND2, CDA, CDO1, CHRD, CRY2, CTSF, CYB5A, CYB5R3, DAB2, DCUN1D4, DPYD, DYNLRB2, ECH1, EIF1, ELMO1, EPB41L3, EPC1, FAM122A, FDX1, FUZ, GABARAP, H3F3B, HMGCL, HSD17B4, ITPR1, MAF, MAP2K4, MCC, MKNK2, MPP5, NR4A2, PGM1, PTPN4, RPS3A, RXRA, SACM1L, SELENBP1, SIK2, SLC7A3, SSBP3, SUCLG2, TK2, UBE4A, UBR2.

Set of novel cancer genes: ALG9, EFHD1, NEGR1, PCF11, RBM5, RGN, RSBN1, SNRK, PQLC3, VPS13D, WDR91, YPEL5, ZDHHC3, TSPAN6, HECA, MTMR10, PEX11G, RAB4A, TRAPPC6B, SLC25A25, COLEC11, RASSF9, SMG6, TSHZ3.

The analysis was conducted using GSE21501 pancreatic cancer patient cohort in PROGgeneV2 prognostic database (http://watson.compbio.iupui.edu/chirayu/proggene/database/index.php).

### Statistics

Statistical analysis using gene expression data was performed with R version 2.5.1 and BioConductor version 1.8. The remaining statistical analyzes were performed on SPSS 18.0. Normally distributed data was statistically analyzed using unpaired two-tailed Student’s t-test for single comparisons, and one-way or two-way analysis of variance–ANOVA- for multiple comparisons. ANOVA analyzes were followed by Bonferroni’s post hoc tests. Data that was ordinary was analyzed using unpaired two-tailed Mann-Whitney test. The statistical test used and p values are indicated in each figure legend. p ≤ 0.05 was considered to indicate statistical significance. Data are shown as mean values ± standard error of the mean (SEM).

### Supplementary data

Supplemental data includes 7 supplemental figures, a supplemental data file and 16 supplemental tables.

## Supporting information

S1 Fig *dRasV12*-induced pupal lethality can be rescued by co-expression of dominant negative dRaf. **(a)** *eyeless-Flp* does not drive expression of the GFP-transgene of the fly driver line in the larval ring gland, which has previously been shown to affect pupariation, confirming previous findings that defective pupal development is due to epithelial tumor formation. Magnification X 40. **(b)** Rescue of pupal lethality in both male and female *dRas*^*V12*^ bearing flies by co-expression of dominant negative (DN) *dRaf* (*dRaf*^*DN*^), confirming that the observed phenotypes are not due to genetic load of the experimental line but due to *dRas*^*V12*^-induced overgrowth of the larval eye-antennal disc. Note that compared to wild type flies (left), *dRas*^*V12*^ *dRaf*^*DN*^ flies exhibited normal eye-morphology (white arrows) but still incomplete formation of the antenna (black arrows) and reduced body size.
(TIF)

S2 Fig Tumor-suppressor-oncogene-scores (TSOS). **(a)** Receiver Operator Characteristics (ROC) for the hit-list among the *Drosophila* orthologs and calculated Area Under the Curve (AUC). **(b)** Tumor suppressor oncogene scores (TSOS) to assess gene expression of our candidate hits in malignancies of the indicated tissues compared to the corresponding normal tissues for all human orthologs of our fly hits (red line) versus the median distribution of gene expression of randomly chosen gene sets (numbers of samplings are indicated for each tumor, n = 619 genes for microarray gene sets and n = 637 for the TCGA and GTEx RNAseq data sets). Only human orthologs of all tested fly genes (S1 Table) were included to avoid bias of comparison. Positive values represent a higher expression in tumors, a negative TSOS shows a higher expression in normal tissues on average. For data on gene expression in tumors and normal tissue see S3 Table. P values are indicated for each tumor type.
(TIF)

S3 Fig A global network map of oncogenic Ras-driven tumorigenesis. Shown are the significantly enriched C2 terms with a nominal p-value ≤0.05 (yellow) for human genes that are ortholog to primary fly *RNAi* hits (red) and first degree binding partners (blue). Only first degree binding partners were included that interact with at least 2 direct hits. The edges denote the protein assignments to C2 terms (grey) and the reported protein-protein interactions in BioGrid 8.0 are shown as green lines. For the entire data, see S8-4 and S8-5 Table.
(TIF)

S4 Fig Analysis of the *Tsp29Fb* knockdown phenotype in *Drosophila*. **(a)** The observed small rough phenotype of *GMR* >*tspi* flies is augmented by reducing endogenous*Tsp29Fb* using a deficiency (*Df; GMR>tspi*), confirming the fidelity of the *Tsp29Fb* shRNAs. Flies were propagated at 25°C and photographed with a black & white CCD camera. **(b)** Expression of *tspi* via the *engrailed* (*en*) driver in the posterior region of the developing wing results in reduced wing size, which was not rescued by blocking apoptosis with expression of the dominant negative JNK transgene (*bskDN*), the *Drosophila* inhibitor of apoptosis *Diap1*, or the effector caspase inhibitor *p35*. Quantification of the wing sizes (pixels) is shown in the bar graph (+/- SEM, n>10 wings per sample). Flies were propagated at 29°C. **(c)** Expression of *tspi* via the *MS1096* driver in the presence of *dicer* in the wing margins results in a reduced wing phenotype. Quantification of the wing sizes (pixels) is shown in the scatter plot (n>10 wings per sample). Flies were propagated at 29°C. **(d)** The efficacy of *Tsp29Fb-RNAi* is revealed by knockdown of HA expression of a HA-tagged *Tsp29Fb* (*tspHA*) transgene; Tsp29Fb-HA protein accumulated apically in wing as well as eye-antennal discs and co-expression of *Tsp29Fb-RNAi* substantially reduced Tsp29Fb protein levels, underscoring the efficacy of shRNA targeting and specificity of the Tsp29Fb knockdown-mediated effects. Top panels: Expression of a *Tsp29Fb-HA* transgene via the *en* driver in third instar larval wing discs stained with anti-HA, GFP and DAPI, shows apical accumulation of Tsp29Fb-HA in the posterior compartment (GFP+). Middle panels: Co-expression of *tspi* with the *Tsp29Fb-HA* transgene results in downregulation of HA staining, indicating that the *RNAi* targets *Tsp29Fb*. Lower panels: Driving *Tsp29Fb-HA* in the posterior region of the third instar larval eye disc using the *GMR* driver, stained with anti-HA, phalloidin (to detect F-actin) and anti-Elav (to detect differentiating neurons), shows that Tsp29Fb-HA is colocalized apically with F-actin. Flies were propagated at 29oC.
(TIF)

S5 Fig Knockdown of Tsp29Fb affects cell morphology, but does not genetically interact with Notch. **(a)** The *GMR*>*tspi* reduced adult eye size is not rescued by co-expression of p35 or DIAP. Flies were propagated at 25°C and photographed with a black & white CCD camera. **(b)** Control (*GMR*) and *GMR>tspi* pupal retinas, stained for *Dlg* and F-actin (phalloidin) and DAPI to detect DNA. Knockdown of *Tsp29Fb* throughout the pupal retina results in disruption of the organized hexagonal array (bottom panel) relative to the wild type eyes (top panel), and *Dlg* and F-actin apical localization is disrupted. **(c)** Notched wing phenotype due to expression of dominant negative mastermind (*mamH*), to inhibit Notch signaling, in the wing margins via the *C96* driver (left panels) is not modified by *tspi* expression. Right bar graphs indicate quantification of wing areas and the wing perimeters in pixels (+/- SEM, n>10 wings per sample) in both male (M) and female (F) flies with the indicated genotypes. No significant differences in *C96>mamH* wing area or perimeter measurements were observed upon *tspi* expression relative to the *GFP* control. Flies were propagated at 29°C.
(TIF)

S6 Fig Tsp29Fb knock-down affects Dlg junctional localization and genetically interacts with the Scrib/Dlg module. (a) Planar confocal images of mosaic pupal retinas from control (top panel), and *Tsp29Fb-RNAi* expressing clones marked with GFP (middle and bottom panel) stained with anti-Dlg (top and middle panels) or anti-Scrib and anti-E-cad (bottom panel), showing that Tsp29Fb affects Dlg localization but not Scrib or E-cad at junctions. Quantification of Dlg abundance in the *Tsp29Fb-RNAi* clones versus wild-type clones indicates that Dlg overall abundance is not significantly affected. **(b)** Western blot analysis of Dlg protein abundance relative to Tubulin in *eyFLP-out* larval eye-antennal discs, showing that Dlg protein abundance is not significantly affected by Tsp29Fb depletion. A representative Western blot is shown probed with anti-Dlg and anti-Tub and the graph is the quantification of 4 independent experiments and 2 technical replicates for each experiment. **(c)** Genetic interaction between *scrib* and *Tsp29Fb* in the adult wing from adult females. Expression of *scrib-RNAi* via *dpp*^*BLK*^*-GAL4* in the region between wing veins L3 and L4, results in reduced wing size, reduced area between wing veins L3 and L4 and breaks in wing vein L3 (bottom left) relative to the *luciferase-RNAi* control (top left), whilst expression of *Tsp29Fb-RNAi* has no effect (top right). Co-expression of *Tsp29Fb-RNAi* with *scrib-RNAi* results in enhancement of the *scrib* knock-down phenotype, resulting in an increase in the breaks in wing vein L3 (bottom right, quantified in the graph), indicating that *Tsp29Fb* genetically interacts with *scrib* in epithelial tissue development. For all experiments, crosses were propagated at 29°C.
(TIF)

S7 Fig Tsp29Fb modulates Ras signaling. **(a)** Scanning electron micrographs of top or side views of adult female eyes of the indicated genotypes, showing that expression of a second *Tsp29Fb-RNAi* line (GD2823, bottom left) enhances the *ey>dRas*^*V12*^ overgrown eye phenotype (top right) similarly to GD2824 (bottom right). **(b)** Images of adult female eyes of the indicated genotypes, showing that expression of *UAS-Tsp29Fb-HA* (bottom right) suppresses the overgrown eye phenotype of the *ey>dRas*^*V12*^ *lacZ* control (bottom left) whereas expression of *UAS-Tsp29Fb-HA* alone (top right) has no effect relative to the *ey>lacZ* control (top left). **(c)** Western blot analysis of pERK, total-ERK protein abundance relative to Tubulin in *eyFLP-out* larval eye-antennal discs, showing that Tsp29Fb depletion results in a significant increase in pERK but is not significantly affect total-ERK relative to Tubulin. A representative Western blot is shown probed with anti-pERK, anti-total-ERK and anti-Tub and the graph is the quantification of 2 independent experiments and 2 technical replicates for each experiment. For all experiments, crosses were propagated at 29°C.
(TIF)

S1 Data File Heat maps of expression data of the top 80, 55 or 25 genes in lung, colon and pancreatic adenocarcinoma TCGA cohorts and association of gene expression with patient survival and Ras mutant status. **(a)** mRNA expression analysis of 80, 55 or 25 genes correlations with patient survival and *KRAS*^*G12*^ mutant status in pancreatic, lung or colon cancer. mRNA expression of the human orthologs of the validated 80 and 55 cancer candidate genes is significantly associated with survival of pancreas cancer patients, but not in lung or colon cancer patients. Data were obtained using K-means clustering on TCGA patient cohorts. P values (log rank test) and total numbers of patients with either low (black lines) or high (red lines) expression of the entire 80 candidate gene set are indicated. Low mRNA expression level of the 80, 55 cancer genes are significantly associated with the *KRAS*^*G12*^ mutational status in human pancreas cancer but not in lung or colon cancer. The *KRAS*^*G12*^ mutational status is shown for the low and high mRNA expression gene sets. None = no mutation; G12 = *KRAS*^*G12*^ mutation; other = mutations in *RAS* other than *KRAS*^*G12*^). Expression heat maps and KRAS mutations are also shown. **(b)** mRNA expression analysis of 80, 55 or 25 genes correlations with patient survival and combined Ras activating mutations (G12, G13, Q61) in *KRAS*, *HaRAS* and *NRAS* in pancreatic, lung or colon cancer. No significant correlation was observed in lung or colon cancer. Expression heat maps and *RAS* mutations are shown. **(c)** Summary table of the specific *RAS* mutations in patient cohorts are shown.
(PDF)

S1 Table *Drosophila* lines screened. All *Drosophila* genes the CG identifiers and gene symbols (http://flybase.org/) are shown corresponding to all screened *Drosophila* transformant lines (TRANSFORMANT_ID) and the corresponding construct identifiers (CONSTRUCT_ID). S19 scores, number of CAN Repeats and number of Off Target genes are indicated for each line. Human and mouse ortholog predictions for *Drosophila* genes are based on Inparanoid v6.1. Shown are the gene symbols for human and mouse seed orthologs (HOMSA, MUSMU) and human and mouse inparalogs (HOMSA-INP, MUSMU-INP). The Table also shows one-to-one and one-to-many relations of each *Drosophila* gene in human and mouse.
(XLS)

S2 Table *Drosophila* primary hits. Invasion phenotypes were noted as appearance of GFP labeled tumor cells on mouth-hooks (MH) of the arrested larval progeny (MH INVASION yes /no (Y/N)) or “metastatic” foci throughout the larval body (FOCI yes/no (Y/N)). Additional observations were noted in the separate column (ADD.REMARKS). The CG identifiers and gene symbols (http://flybase.org/) are shown corresponding to all identified primary *Drosophila* hit transformant lines (TRANSFORMANT_ID) and corresponding construct identifiers (CONSTRUCT_ID). The CG identifier, S19 scores, number of CAN Repeats and number of Off Targets genes are indicated. Only hits with an S19 score ≥ 0.8 and CAN repeats ≤ 6 were considered candidate genes. The gene symbols for human and mouse seed orthologs (HOMSA, MUSMU) and human and mouse inparalog orthologs (HOMSA-INP, MUSMU-INP) are also shown.
(XLS)

S3 Table Differential expression of human seed orthologs in human tumors. **Table S3-0:** Differential expression of human seed ortholog candidate genes in human tumors. Table S3-0 contains the direction scores (higher expression in Tumor +1, higher in Normal Tissue -1) which are the basis for the TSOS for all human genes that are seed orthologs to our primary direct fly hits. If more than one probeSet is available on the Affymetrix chip, the direction is divided by the number of probe sets for this gene (even when multiple probe sets per gene are present on the chip). "ss1" (Sample set number 1 (column O) = normal Tissue), "ss2" (Sample Set 2 (column P) = tumor tissue). Direction value of the gene divided by the number of probe sets indicates the extent of down-regulation (beige) or upregulation in tumors as compared to the corresponding normal tissue of the same organ.
**Table S3-1: Gene probes and tissues used for differential expression analysis of human seed ortholog candidate genes in human tumors.** List of genes with full gene symbols (symbol), names of genes (geneName) with their respective chromosomal localization (localization) and ENSEMBL gene IDs (ensemblGene). Probes that show cross-hybridisation (crossHyb) have not been included in the analysis. Column ss1 shows a list of normal human tissues and ss2-coressponding matching tumor tissues used for the differential expression analysis. Number of probes for each gene present on the chip is given in column K.
(XLS)

S4 Table Top 100 human seed ortholog candidate genes with maximum differential expression scores in human tumors. Table contains the list of 100 human seed ortholog genes to our primary direct fly hits with the highest TSOS scores. If more than one probe set is available on the Affymetrix chip, the direction is divided by the number of probe sets for this gene (even when multiple probe sets per gene are present on the chip). "ss1" (Sample Set number 1 (column O) = normal Tissue), "ss2" (Sample Set 2 (column P) = tumor tissue). Direction value of the gene divided by the number of probe sets indicates the extent of down-regulation (beige) or upregulation in tumors as compared to the corresponding normal tissue of the same organ.
(XLSX)

S5 Table Secondary screen for 100 top ranking hits. Summary of secondary *RNAi* screen results in the fly, preselected based on the top 100 primary hits of gene found to be downregulated in multiple human tumors (see also S4 Table). Yellow highlighted lines were observed to result in enhanced tumorigenesis in the primary screen (see S2 Table). Grey highlighted genes indicate those where the average score was less than 0.75.
(XLSX)

S6 Table Significantly enriched *Drosophila* GO terms and KEGG pathways. Gene Ontology and KEGG pathway enrichment analysis for *Drosophil*a candidate genes (primary hits) with and without binding partners. **Table S6-0.** All *Drosophila* candidate genes (HIT_CG and HIT_GENE_SYMBOL) with corresponding binding partners (BioGrid_CG and Biogrid_GENE SYMBOL) defined in BioGrid v8.0. Binding partners were only added when protein-protein interactions were assigned to at least two *Drosophila* candidate genes. **Tables S6-1 to S6-6.** All significantly enriched GO terms with p< = 0.05 analyzed for *Drosophila* candidate genes without and with binding partners (+BP) in the 3 GO domains Biological process (GOBP), Molecular function (GOMF) and Cellular Component (GOCC). **Table S6-7 and S6-8.** All over-presented KEGGids with a nominal p-value p≤0.05 analysed for *Drosophila* candidate genes without and with binding partners (+BP).
**Table S6-0**. List of primary *Drosophila* hits with binding partners, Sheet label: DROME+BP list. **Table S6-1**: Gene ontology enrichment analysis for primary *Drosophila* hits (Biological Process), Sheet label: GOBP.DROME. **Table S6-2**. Gene ontology enrichment analysis for primary *Drosophila* + binding partners (Biological Process), Sheet label: GOBP.DROME+BP. **Table S6-3**. Gene ontology enrichment analysis for primary *Drosophila* hits (Cellular Component), Sheet label: GOCC.DROME. **Table S6-4**. Gene ontology enrichment analysis for primary *Drosophila* hits + binding partners (Cellular Component), Sheet label: GOCC.DROME+BP. **Table S6-5.** Gene ontology enrichment analysis for primary *Drosophila* hits (Molecular Function), Sheet label: GOMF.DROME. **Table S6-6.** Gene ontology enrichment analysis for primary *Drosophila* hits + binding partners (Molecular Function), Sheet label: GOMF.DROME+BP.
(XLS)

S7 Table Significantly enriched human GO terms. GO enrichment analysis for human seed orthologs and inparalog candidate genes without and with binding partners (+BP). **Tables S7-0 and S7-1.** All human seed ortholog and inparalog candidate genes (HIT_ENSG and HIT_GENE SYMBOL) with corresponding binding partners defined in BioGrid v8.0 (BioGrid_ENSG and BioGrid_GENE SYMBOL). Binding partners were only added when protein-protein interactions were assigned to at least two human candidate genes. **Tables S7-2 to S7-13.** Significantly enriched GO terms with p≤0.05 analyzed for human seed orthologs and inparalog candidate genes with and without binding partners (+BP) in the 3 GO domains Biological process (GOBP), Molecular function (GOMF) and Cellular Component (GOCC).
**Table S7-0.** Human seed orthologs and binding partners, Sheet label: HSA.SEED+BP list. **Table S7-1**. Human inparalogs and binding partners, Sheet label: HSA.INPARALOG+BP list. **Table S7-2**: Gene ontology enrichment analysis Biological Process (Human Seed Orthologs), Sheet label: GOBP.HSA.SEED. **Table S7-3**. Gene ontology enrichment analysis Biological Process (Human Seed Orthologs + Binding Partners), Sheet label: GOBP.HSA.SEED+BP. **Table S7-4.** Gene ontology enrichment analysis Biological Process (Human Inparalogs), Sheet label: GOBP.HSA.INPARALOG. **Table S7-5**. Gene ontology enrichment analysis Biological Process (Human Inparalogs + Binding Partner), Sheet label: GOBP.HSA.INPARALOG+BP. **Table S7-6.** Gene ontology enrichment analysis Molecular Function (Human Seed Orthologs), Sheet label: GOMF.HSA.SEED. **Table S7-7**. Gene ontology enrichment analysis Molecular Function (Human Seed Orthologs + Binding Partners), Sheet label: GOMF.HSA.SEED+BP. **Table S7-8.** Gene ontology enrichment analysis Molecular Function (Human Inparalogs), Sheet label: GOMF.HSA.INPARALOG. **Table S7-9**. Gene ontology enrichment analysis Molecular Function (Human Inparalogs + Binding Partner), Sheet label: GOMF.HSA.INPARALOG+BP. **Table S7-10.** Gene ontology enrichment analysis Cellular Component (Human Seed Orthologs), Sheet label: GOCC.HSA.SEED. **Table S7-11**. Gene ontology enrichment analysis Cellular Component (Human Seed Orthologs + Binding Partners), Sheet label: GOCC.HSA.SEED+BP. **Table S7-12**. Gene ontology enrichment analysis Cellular Component (Human Inparalogs), Sheet label: GOCC.HSA.INPARALOG. **Table 7–13**. Gene ontology enrichment analysis Cellular Component (Human Inparalogs + Binding Partner), Sheet label: GOCC.HSA.INPARALOG+BP.
(XLS)

S8 Table Significantly enriched KEGG pathways and Human Curated gene set (C2) terms. KEGG pathway and Curated gene set (C2) database enrichment analyses for human seed orthologs and inparalog candidate genes with and without binding partners (+BP). For seed orthologs, inparalog candidate genes, and inclusion of binding partners see **Table S8-0**. **Tables S8-1 to S8-5** show significant KEGG ids with a nominal p-value p≤0.05. **Tables S8-6 to S8-9** show all significant C2 terms with a nominal p-value p≤0.05. For each C2 term, the fraction of candidate genes, the number of candidate genes, numbers of genes annotated to a particular C2 term, the expected number of candidate genes (to exclude a random distribution), the nominal p-value, the FDR (false discovery rate) value, gene symbols for all significant candidate genes, and gene symbols for all annotated genes are shown.
**Table S8-0**. KEGG pathway enrichment analysis (Human Seed Orthologs). **Table 8–1.** KEGG pathway enrichment analysis (Human Seed Orthologs + Binding Partners). **Table S8-2.** KEGG pathway enrichment analysis (Human Seed Orthologs), Sheet label: KEGG.HSA.SEED. **Table S8-3.** KEGG pathway enrichment analysis (Human Seed Orthologs + binding Partner), Sheet label: KEGG.HSA.SEED+BP. **Table S8-4.** KEGG pathway enrichment analysis (Human Inparalogs), Sheet label: KEGG.HSA.INPARALOG. **Table S8-5.** KEGG pathway enrichment analysis (Human Inparalogs + Binding Partners), Sheet label: KEGG.HSA.INPARALOG+BP. **Table S8-6.** Curated gene set (C2) enrichment analysis (Human Seed Orthologs), Sheet label: C2.HSA.SEED. **Table S8-7.** Curated gene set (C2) enrichment analysis (Human Seed Orthologs + binding Partner), Sheet label: C2.HSA.SEED+BP. **Table S8-8.** Curated gene set (C2) enrichment analysis (Human Inparalogs), Sheet label: C2.HSA.INPARALOG. **Table S8-9.** Curated gene set (C2) enrichment analysis (Human Inparalogs + Binding Partners), Sheet label: C2.HSA.INPARALOG+BP.
(XLSX)

S9 Table Signaling module analysis for the top 80 human seed orthologs with binding partners. An enrichment analysis for signaling pathways based on a hypergeometric test for the selected 80 tumor suppressor candidates (TSC) including their direct protein interaction partners (TSCi). Direct interaction partners interacting with at least two TSC genes were considered for the analysis. Shown are the pathway database name (DB), pathway name, the number of TSC/TSCi, the total number of genes included (annotated), p-values, the FDR (false discovery rate) values and the respective gene names of TSC and TSCi annotated to the individual pathways.
(XLS)

S10 Table Quantification of Dlg protein abundance in *Tsp29Fb-RNAi* clones compared with wild-type clones. Quantification of data shown in S6A Fig.
(XLSX)

S11 Table Quantification of Dlg protein abundance in *ey-FLPout Tsp29Fb-RNAi* eye epithelial tissue compared to the control. Quantification of data shown in S6B Fig.
(XLSX)

S12 Table Quantification of wing vein loss in *dpp>scrib-RNAi* upon *Tsp29Fb* knockdown relative to the *scrib* knockdown alone. Quantification of data shown in S6C Fig.
(XLSX)

S13 Table Quantification of the adult eye size of *ey>dRas^V12^* upon *Tsp29Fb* knockdown relative to *ey>dRas^V12^* alone and wild-type. Quantification of data shown in Fig 7C.
(XLSX)

S14 Table Quantification of the effect of Tsp29Fb-HA expression on adult eye size of ey> and ey>dRas^V12^. Quantification of data shown in S7B Fig.
(XLSX)

S15 Table Quantification of the effect of Tsp29Fb knockdown on EGFR-Ras signalling, as assayed by pERK abundance, in *dRas^V12^* expressing epithelial tissue. Quantification of data shown in Fig 7D.
(XLSX)

S16 Table Quantification of the effect of Tsp29Fb knockdown on EGFR-Ras signalling, as assayed by pERK abundance, in wild-type epithelial tissue. Quantification of data shown in S7C Fig.
(XLSX)

## References

[1] Young A, Lyons J, Miller AL, Phan VT, Alarcon IR, McCormick F. Ras signaling and therapies. Advances in cancer research. 2009;102:1–17. 10.1016/S0065-230X(09)02001-6 . 19595305

[2] Dimauro T, David G. Ras-induced senescence and its physiological relevance in cancer. Current cancer drug targets. 2010;10(8):869–76. ; PubMed Central PMCID: PMC4023163. 20718709 10.2174/156800910793357998 PMC4023163

[3] Krauthammer M, Kong Y, Bacchiocchi A, Evans P, Pornputtapong N, Wu C, et al Exome sequencing identifies recurrent mutations in NF1 and RASopathy genes in sun-exposed melanomas. Nature genetics. 2015;47(9):996–1002. 10.1038/ng.3361 . 26214590 PMC4916843

[4] Steeg PS. Tumor metastasis: mechanistic insights and clinical challenges. Nature medicine. 2006;12(8):895–904. 10.1038/nm1469 . 16892035

[5] Bacac M, Stamenkovic I. Metastatic cancer cell. Annual review of pathology. 2008;3:221–47. 10.1146/annurev.pathmechdis.3.121806.151523 . 18233952

[6] Chiang AC, Massague J. Molecular basis of metastasis. The New England journal of medicine. 2008;359(26):2814–23. 10.1056/NEJMra0805239 ; PubMed Central PMCID: PMC4189180. 19109576 PMC4189180

[7] Kang Y, Pantel K. Tumor cell dissemination: emerging biological insights from animal models and cancer patients. Cancer cell. 2013;23(5):573–81. 10.1016/j.ccr.2013.04.017 ; PubMed Central PMCID: PMC3667710. 23680145 PMC3667710

[8] Brumby AM, Richardson HE. Using Drosophila melanogaster to map human cancer pathways. Nature reviews Cancer. 2005;5(8):626–39. 10.1038/nrc1671 . 16034367

[9] Homem CC, Knoblich JA. Drosophila neuroblasts: a model for stem cell biology. Development. 2012;139(23):4297–310. 10.1242/dev.080515 . 23132240

[10] Artavanis-Tsakonas S, Matsuno K, Fortini ME. Notch signaling. Science. 1995;268(5208):225–32. . 7716513 10.1126/science.7716513

[11] Jindal GA, Goyal Y, Burdine RD, Rauen KA, Shvartsman SY. RASopathies: unraveling mechanisms with animal models. Disease models & mechanisms. 2015;8(8):769–82. 10.1242/dmm.020339 ; PubMed Central PMCID: PMC4527292. 26203125 PMC4527292

[12] Ntziachristos P, Lim JS, Sage J, Aifantis I. From fly wings to targeted cancer therapies: a centennial for notch signaling. Cancer cell. 2014;25(3):318–34. 10.1016/j.ccr.2014.02.018 ; PubMed Central PMCID: PMC4040351. 24651013 PMC4040351

[13] Miles WO, Dyson NJ, Walker JA. Modeling tumor invasion and metastasis in Drosophila. Disease models & mechanisms. 2011;4(6):753–61. 10.1242/dmm.006908 ; PubMed Central PMCID: PMC3209645. 21979943 PMC3209645

[14] Brumby AM, Richardson HE. scribble mutants cooperate with oncogenic Ras or Notch to cause neoplastic overgrowth in Drosophila. The EMBO journal. 2003;22(21):5769–79. 10.1093/emboj/cdg548 ; PubMed Central PMCID: PMC275405. 14592975 PMC275405

[15] Pagliarini RA, Xu T. A genetic screen in Drosophila for metastatic behavior. Science. 2003;302(5648):1227–31. 10.1126/science.1088474 . 14551319

[16] Stefanatos RK, Vidal M. Tumor invasion and metastasis in Drosophila: a bold past, a bright future. Journal of genetics and genomics = Yi chuan xue bao. 2011;38(10):431–8. 10.1016/j.jgg.2011.09.004 . 22035864

[17] Neuman-Silberberg FS, Schejter E, Hoffmann FM, Shilo BZ. The Drosophila ras oncogenes: structure and nucleotide sequence. Cell. 1984;37(3):1027–33. . 6430564 10.1016/0092-8674(84)90437-9

[18] Borjeson H, Fellenius E. Towards a valid technique of sampling fish muscle to determine redox substrates. Acta physiologica Scandinavica. 1976;96(2):202–6. 10.1111/j.1748-1716.1976.tb10189.x . 1258670

[19] Gafuik C, Steller H. A gain-of-function germline mutation in Drosophila ras1 affects apoptosis and cell fate during development. PloS one. 2011;6(8):e23535 10.1371/journal.pone.0023535 ; PubMed Central PMCID: PMC3155559. 21858158 PMC3155559

[20] Ashburner M, Misra S, Roote J, Lewis SE, Blazej R, Davis T, et al An exploration of the sequence of a 2.9-Mb region of the genome of Drosophila melanogaster: the Adh region. Genetics. 1999;153(1):179–219. ; PubMed Central PMCID: PMC1460734. 10471707 10.1093/genetics/153.1.179 PMC1460734

[21] Hobbs GA, Der CJ, Rossman KL. RAS isoforms and mutations in cancer at a glance. Journal of cell science. 2016;129(7):1287–92. 10.1242/jcs.182873 ; PubMed Central PMCID: PMC4869631. 26985062 PMC4869631

[22] McGuire SE, Roman G, Davis RL. Gene expression systems in Drosophila: a synthesis of time and space. Trends in genetics: TIG. 2004;20(8):384–91. 10.1016/j.tig.2004.06.012 . 15262411

[23] Symons M, Takai Y. Ras GTPases: singing in tune. Sci STKE. 2001;2001(68):PE1 10.1126/stke.2001.68.pe1 . 11752638

[24] Uhlirova M, Bohmann D. JNK- and Fos-regulated Mmp1 expression cooperates with Ras to induce invasive tumors in Drosophila. The EMBO journal. 2006;25(22):5294–304. 10.1038/sj.emboj.7601401 ; PubMed Central PMCID: PMC1636619. 17082773 PMC1636619

[25] Dominguez M, Casares F. Organ specification-growth control connection: new in-sights from the Drosophila eye-antennal disc. Developmental dynamics: an official publication of the American Association of Anatomists. 2005;232(3):673–84. 10.1002/dvdy.20311 . 15704149

[26] Brumby AM, Goulding KR, Schlosser T, Loi S, Galea R, Khoo P, et al Identification of novel Ras-cooperating oncogenes in Drosophila melanogaster: a RhoGEF/Rho-family/JNK pathway is a central driver of tumorigenesis. Genetics. 2011;188(1):105–25. 10.1534/genetics.111.127910 ; PubMed Central PMCID: PMC3120157. 21368274 PMC3120157

[27] Vissers JH, Manning SA, Kulkarni A, Harvey KF. A Drosophila *RNAi* library modulates Hippo pathway-dependent tissue growth. Nature communications. 2016;7:10368 10.1038/ncomms10368 ; PubMed Central PMCID: PMCPMC4735554. 26758424 PMC4735554

[28] Manent J, Banerjee S, de Matos Simoes R, Zoranovic T, Mitsiades C, Penninger JM, et al Autophagy suppresses Ras-driven epithelial tumourigenesis by limiting the accumulation of reactive oxygen species. Oncogene. 2017;36(40):5576–92. 10.1038/onc.2017.175 ; PubMed Central PMCID: PMCPMC5633656. 28581519 PMC5633656

[29] Pascual J, Jacobs J, Sansores-Garcia L, Natarajan M, Zeitlinger J, Aerts S, et al Hippo Reprograms the Transcriptional Response to Ras Signaling. Developmental cell. 2017;42(6):667–80 e4. 10.1016/j.devcel.2017.08.013 . 28950103

[30] Duman-Scheel M. Netrin and DCC: axon guidance regulators at the intersection of nervous system development and cancer. Current drug targets. 2009;10(7):602–10. ; PubMed Central PMCID: PMC2756184. 19601764 10.2174/138945009788680428 PMC2756184

[31] D'Armiento J, Shiomi T, Marks S, Geraghty P, Sankarasharma D, Chada K. Mesenchymal Tumorigenesis Driven by TSC2 Haploinsufficiency Requires HMGA2 and Is Independent of mTOR Pathway Activation. Cancer research. 2016;76(4):844–54. 10.1158/0008-5472.CAN-15-1287 . 26837766 PMC5554010

[32] Tsun ZY, Bar-Peled L, Chantranupong L, Zoncu R, Wang T, Kim C, et al The folliculin tumor suppressor is a GAP for the RagC/D GTPases that signal amino acid levels to mTORC1. Molecular cell. 2013;52(4):495–505. 10.1016/j.molcel.2013.09.016 ; PubMed Central PMCID: PMC3867817. 24095279 PMC3867817

[33] Moll F, Millet C, Noel D, Orsetti B, Bardin A, Katsaros D, et al Chordin is underexpressed in ovarian tumors and reduces tumor cell motility. FASEB journal: official publication of the Federation of American Societies for Experimental Biology. 2006;20(2):240–50. 10.1096/fj.05-4126com . 16449796

[34] Humbert PO, Grzeschik NA, Brumby AM, Galea R, Elsum I, Richardson HE. Control of tumourigenesis by the Scribble/Dlg/Lgl polarity module. Oncogene. 2008;27(55):6888–907. 10.1038/onc.2008.341 . 19029932

[35] Chia WJ, Tang BL. Emerging roles for Rab family GTPases in human cancer. Biochimica et biophysica acta. 2009;1795(2):110–6. . 19425190 10.1016/j.bbcan.2008.10.001

[36] Taube JH, Herschkowitz JI, Komurov K, Zhou AY, Gupta S, Yang J, et al Core epithelial-to-mesenchymal transition interactome gene-expression signature is associated with claudin-low and metaplastic breast cancer subtypes. Proceedings of the National Academy of Sciences of the United States of America. 2010;107(35):15449–54. 10.1073/pnas.1004900107 ; PubMed Central PMCID: PMC2932589. 20713713 PMC2932589

[37] Liberzon A, Subramanian A, Pinchback R, Thorvaldsdottir H, Tamayo P, Mesirov JP. Molecular signatures database (MSigDB) 3.0. Bioinformatics. 2011;27(12):1739–40. 10.1093/bioinformatics/btr260 ; PubMed Central PMCID: PMC3106198. 21546393 PMC3106198

[38] Subramanian A, Kuehn H, Gould J, Tamayo P, Mesirov JP. GSEA-P: a desktop application for Gene Set Enrichment Analysis. Bioinformatics. 2007;23(23):3251–3. 10.1093/bioinformatics/btm369 . 17644558

[39] Sawa M, Masuda M, Yamada T. Targeting the Wnt signaling pathway in colorectal cancer. Expert opinion on therapeutic targets. 2015:1–11. 10.1517/14728222.2016.1098619 . 26439805

[40] Carbone C, Tamburrino A, Piro G, Boschi F, Cataldo I, Zanotto M, et al Combined inhibition of IL1, CXCR1/2, and TGFbeta signaling pathways modulates in-vivo resistance to anti-VEGF treatment. Anti-cancer drugs. 2015 10.1097/CAD.0000000000000301 . 26473526

[41] Lin L, Sabnis AJ, Chan E, Olivas V, Cade L, Pazarentzos E, et al The Hippo effector YAP promotes resistance to RAF- and MEK-targeted cancer therapies. Nature genetics. 2015;47(3):250–6. 10.1038/ng.3218 ; PubMed Central PMCID: PMC4930244. 25665005 PMC4930244

[42] Lin JI, Mitchell NC, Kalcina M, Tchoubrieva E, Stewart MJ, Marygold SJ, et al Drosophila ribosomal protein mutants control tissue growth non-autonomously via effects on the prothoracic gland and ecdysone. PLoS genetics. 2011;7(12):e1002408 10.1371/journal.pgen.1002408 ; PubMed Central PMCID: PMC3240600. 22194697 PMC3240600

[43] Giry-Laterriere M, Pinho AV, Eling N, Chantrill L, Rooman I. Emerging Drug Target In Pancreatic Cancer: Placing Sirtuin 1 on the Canvas. Current cancer drug targets. 2015;15(6):463–8. . 26282546 10.2174/1568009615666150512102957

[44] Oon CE, Strell C, Yeong KY, Ostman A, Prakash J. SIRT1 inhibition in pancreatic cancer models: contrasting effects in vitro and in vivo. European journal of pharmacology. 2015;757:59–67. 10.1016/j.ejphar.2015.03.064 . 25843411

[45] Rosati A, Basile A, D'Auria R, d'Avenia M, De Marco M, Falco A, et al BAG3 promotes pancreatic ductal adenocarcinoma growth by activating stromal macrophages. Nature communications. 2015;6:8695 10.1038/ncomms9695 ; PubMed Central PMCID: PMC4659838. 26522614 PMC4659838

[46] Rosati A, Bersani S, Tavano F, Dalla Pozza E, De Marco M, Palmieri M, et al Expression of the antiapoptotic protein BAG3 is a feature of pancreatic adenocarcinoma and its overexpression is associated with poorer survival. The American journal of pathology. 2012;181(5):1524–9. 10.1016/j.ajpath.2012.07.016 . 22944597

[47] Yau CY, Wheeler JJ, Sutton KL, Hedley DW. Inhibition of integrin-linked kinase by a selective small molecule inhibitor, QLT0254, inhibits the PI3K/PKB/mTOR, Stat3, and FKHR pathways and tumor growth, and enhances gemcitabine-induced apoptosis in human orthotopic primary pancreatic cancer xenografts. Cancer research. 2005;65(4):1497–504. 10.1158/0008-5472.CAN-04-2940 . 15735038

[48] Schaeffer DF, Assi K, Chan K, Buczkowski AK, Chung SW, Scudamore CH, et al Tumor expression of integrin-linked kinase (ILK) correlates with the expression of the E-cadherin repressor snail: an immunohistochemical study in ductal pancreatic adenocarcinoma. Virchows Archiv: an international journal of pathology. 2010;456(3):261–8. 10.1007/s00428-009-0866-z . 20091050

[49] Chu PC, Yang MC, Kulp SK, Salunke SB, Himmel LE, Fang CS, et al Regulation of oncogenic KRAS signaling via a novel KRAS-integrin-linked kinase-hnRNPA1 regulatory loop in human pancreatic cancer cells. Oncogene. 2015 10.1038/onc.2015.458 . 26616862

[50] Feldmann G, Mishra A, Bisht S, Karikari C, Garrido-Laguna I, Rasheed Z, et al Cyclin-dependent kinase inhibitor Dinaciclib (SCH727965) inhibits pancreatic cancer growth and progression in murine xenograft models. Cancer biology & therapy. 2011;12(7):598–609. 10.4161/cbt.12.7.16475 ; PubMed Central PMCID: PMC3218385. 21768779 PMC3218385

[51] Hu C, Dadon T, Chenna V, Yabuuchi S, Bannerji R, Booher R, et al Combined Inhibition of Cyclin-Dependent Kinases (Dinaciclib) and AKT (MK-2206) Blocks Pancreatic Tumor Growth and Metastases in Patient-Derived Xenograft Models. Molecular cancer therapeutics. 2015;14(7):1532–9. 10.1158/1535-7163.MCT-15-0028 ; PubMed Central PMCID: PMC4497872. 25931518 PMC4497872

[52] Feldmann G, Mishra A, Hong SM, Bisht S, Strock CJ, Ball DW, et al Inhibiting the cyclin-dependent kinase CDK5 blocks pancreatic cancer formation and progression through the suppression of Ras-Ral signaling. Cancer research. 2010;70(11):4460–9. 10.1158/0008-5472.CAN-09-1107 ; PubMed Central PMCID: PMC3071300. 20484029 PMC3071300

[53] Bang D, Wilson W, Ryan M, Yeh JJ, Baldwin AS. GSK-3alpha promotes oncogenic KRAS function in pancreatic cancer via TAK1-TAB stabilization and regulation of noncanonical NF-kappaB. Cancer discovery. 2013;3(6):690–703. 10.1158/2159-8290.CD-12-0541 ; PubMed Central PMCID: PMC3679268. 23547054 PMC3679268

[54] Damaskos C, Garmpis N, Karatzas T, Nikolidakis L, Kostakis ID, Garmpi A, et al Histone Deacetylase (HDAC) Inhibitors: Current Evidence for Therapeutic Activities in Pancreatic Cancer. Anticancer research. 2015;35(6):3129–35. . 26026072

[55] Prior IA, Lewis PD, Mattos C. A comprehensive survey of Ras mutations in cancer. Cancer research. 2012;72(10):2457–67. 10.1158/0008-5472.CAN-11-2612 ; PubMed Central PMCID: PMCPMC3354961. 22589270 PMC3354961

[56] Rubinstein E. The complexity of tetraspanins. Biochemical Society transactions. 2011;39(2):501–5. 10.1042/BST0390501 . 21428928

[57] Grzeschik NA, Parsons LM, Allott ML, Harvey KF, Richardson HE. Lgl, aPKC, and Crumbs regulate the Salvador/Warts/Hippo pathway through two distinct mechanisms. Current biology: CB. 2010;20(7):573–81. 10.1016/j.cub.2010.01.055 . 20362447

[58] Lesokhin AM, Yu SY, Katz J, Baker NE. Several levels of EGF receptor signaling during photoreceptor specification in wild-type, Ellipse, and null mutant Drosophila. Developmental biology. 1999;205(1):129–44. 10.1006/dbio.1998.9121 . 9882502

[59] Karim FD, Chang HC, Therrien M, Wassarman DA, Laverty T, Rubin GM. A screen for genes that function downstream of Ras1 during Drosophila eye development. Genetics. 1996;143(1):315–29. ; PubMed Central PMCID: PMC1207264. 8722784 10.1093/genetics/143.1.315 PMC1207264

[60] Dickson BJ, van der Straten A, Dominguez M, Hafen E. Mutations Modulating Raf signaling in Drosophila eye development. Genetics. 1996;142(1):163–71. ; PubMed Central PMCID: PMC1206944. 8770593 10.1093/genetics/142.1.163 PMC1206944

[61] Miklos GL, Rubin GM. The role of the genome project in determining gene function: insights from model organisms. Cell. 1996;86(4):521–9. . 8752207 10.1016/s0092-8674(00)80126-9

[62] Vef O, Cleppien D, Loffler T, Altenhein B, Technau GM. A new strategy for efficient in vivo screening of mutagenized Drosophila embryos. Development genes and evolution. 2006;216(2):105–8. 10.1007/s00427-005-0036-5 . 16328480

[63] Chen R, Amoui M, Zhang Z, Mardon G. Dachshund and eyes absent proteins form a complex and function synergistically to induce ectopic eye development in Drosophila. Cell. 1997;91(7):893–903. . 9428513 10.1016/s0092-8674(00)80481-x

[64] Bleau AM, Freire J, Pajares MJ, Zudaire I, Anton I, Nistal-Villan E, et al New syngeneic inflammatory-related lung cancer metastatic model harboring double KRAS/WWOX alterations. International journal of cancer Journal international du cancer. 2014;135(11):2516–27. 10.1002/ijc.28574 . 24473991

[65] Zuidscherwoude M, Dunlock VE, van den Bogaart G, van Deventer SJ, van der Schaaf A, van Oostrum J, et al Tetraspanin microdomains control localized protein kinase C signaling in B cells. Science signaling. 2017;10(478). 10.1126/scisignal.aag2755 . 28487417

[66] Delos Santos RC, Garay C, Antonescu CN. Charming neighborhoods on the cell surface: plasma membrane microdomains regulate receptor tyrosine kinase signaling. Cell Signal. 2015;27(10):1963–76. 10.1016/j.cellsig.2015.07.004 . 26163824

[67] Termini CM, Gillette JM. Tetraspanins Function as Regulators of Cellular Signaling. Front Cell Dev Biol. 2017;5:34 10.3389/fcell.2017.00034 ; PubMed Central PMCID: PMCPMC5382171. 28428953 PMC5382171

[68] Dow LE, Elsum IA, King CL, Kinross KM, Richardson HE, Humbert PO. Loss of human Scribble cooperates with H-Ras to promote cell invasion through deregulation of MAPK signalling. Oncogene. 2008;27(46):5988–6001. 10.1038/onc.2008.219 . 18641685

[69] Nagasaka K, Pim D, Massimi P, Thomas M, Tomaic V, Subbaiah VK, et al The cell polarity regulator hScrib controls ERK activation through a KIM site-dependent interaction. Oncogene. 2010;29(38):5311–21. 10.1038/onc.2010.265 . 20622900

[70] Rives-Quinto N, Franco M, de Torres-Jurado A, Carmena A. Synergism between canoe and scribble mutations causes tumor-like overgrowth via Ras activation in neural stem cells and epithelia. Development. 2017;144(14):2570–83. 10.1242/dev.148171 . 28619817

[71] Dietzl G, Chen D, Schnorrer F, Su KC, Barinova Y, Fellner M, et al A genome-wide transgenic *RNAi* library for conditional gene inactivation in Drosophila. Nature. 2007;448(7150):151–6. 10.1038/nature05954 . 17625558

[72] Gallio M, Englund C, Kylsten P, Samakovlis C. Rhomboid 3 orchestrates Slit-independent repulsion of tracheal branches at the CNS midline. Development. 2004;131(15):3605–14. 10.1242/dev.01242 . 15229181

[73] Hauck B, Gehring WJ, Walldorf U. Functional analysis of an eye specific enhancer of the eyeless gene in Drosophila. Proceedings of the National Academy of Sciences of the United States of America. 1999;96(2):564–9. ; PubMed Central PMCID: PMCPMC15176. 9892673 10.1073/pnas.96.2.564 PMC15176

[74] Hazelett DJ, Bourouis M, Walldorf U, Treisman JE. decapentaplegic and wingless are regulated by eyes absent and eyegone and interact to direct the pattern of retinal differentiation in the eye disc. Development. 1998;125(18):3741–51. . 9716539 10.1242/dev.125.18.3741

[75] Hay BA, Maile R, Rubin GM. P element insertion-dependent gene activation in the Drosophila eye. Proceedings of the National Academy of Sciences of the United States of America. 1997;94(10):5195–200. ; PubMed Central PMCID: PMCPMC24655. 9144214 10.1073/pnas.94.10.5195 PMC24655

[76] Weber U, Paricio N, Mlodzik M. Jun mediates Frizzled-induced R3/R4 cell fate distinction and planar polarity determination in the Drosophila eye. Development. 2000;127(16):3619–29. . 10903185 10.1242/dev.127.16.3619

[77] Hay BA, Wolff T, Rubin GM. Expression of baculovirus P35 prevents cell death in Drosophila. Development. 1994;120(8):2121–9. . 7925015 10.1242/dev.120.8.2121

[78] Kuzniar A, van Ham RC, Pongor S, Leunissen JA. The quest for orthologs: finding the corresponding gene across genomes. Trends in genetics: TIG. 2008;24(11):539–51. 10.1016/j.tig.2008.08.009 . 18819722

[79] Smedley D, Haider S, Ballester B, Holland R, London D, Thorisson G, et al BioMart—biological queries made easy. BMC genomics. 2009;10:22 10.1186/1471-2164-10-22 ; PubMed Central PMCID: PMC2649164. 19144180 PMC2649164

[80] Alexa A, Rahnenfuhrer J, Lengauer T. Improved scoring of functional groups from gene expression data by decorrelating GO graph structure. Bioinformatics. 2006;22(13):1600–7. 10.1093/bioinformatics/btl140 . 16606683

[81] Gentleman RC, Carey VJ, Bates DM, Bolstad B, Dettling M, Dudoit S, et al Bioconductor: open software development for computational biology and bioinformatics. Genome biology. 2004;5(10):R80 10.1186/gb-2004-5-10-r80 ; PubMed Central PMCID: PMC545600. 15461798 PMC545600

[82] Lawrence MS, Stojanov P, Polak P, Kryukov GV, Cibulskis K, Sivachenko A, et al Mutational heterogeneity in cancer and the search for new cancer-associated genes. Nature. 2013;499(7457):214–8. 10.1038/nature12213 ; PubMed Central PMCID: PMC3919509. 23770567 PMC3919509

