# Supplementary figures and images for: A genome-wide Drosophila epithelial tumorigenesis screen identifies Tetraspanin 29Fb as an evolutionarily conserved suppressor of Ras-driven cancer
Source: PLoS Genet. 2018 Oct 16;14(10):e1007688. doi: 10.1371/journal.pgen.1007688 (PMC6203380; doi:10.1371/journal.pgen.1007688)

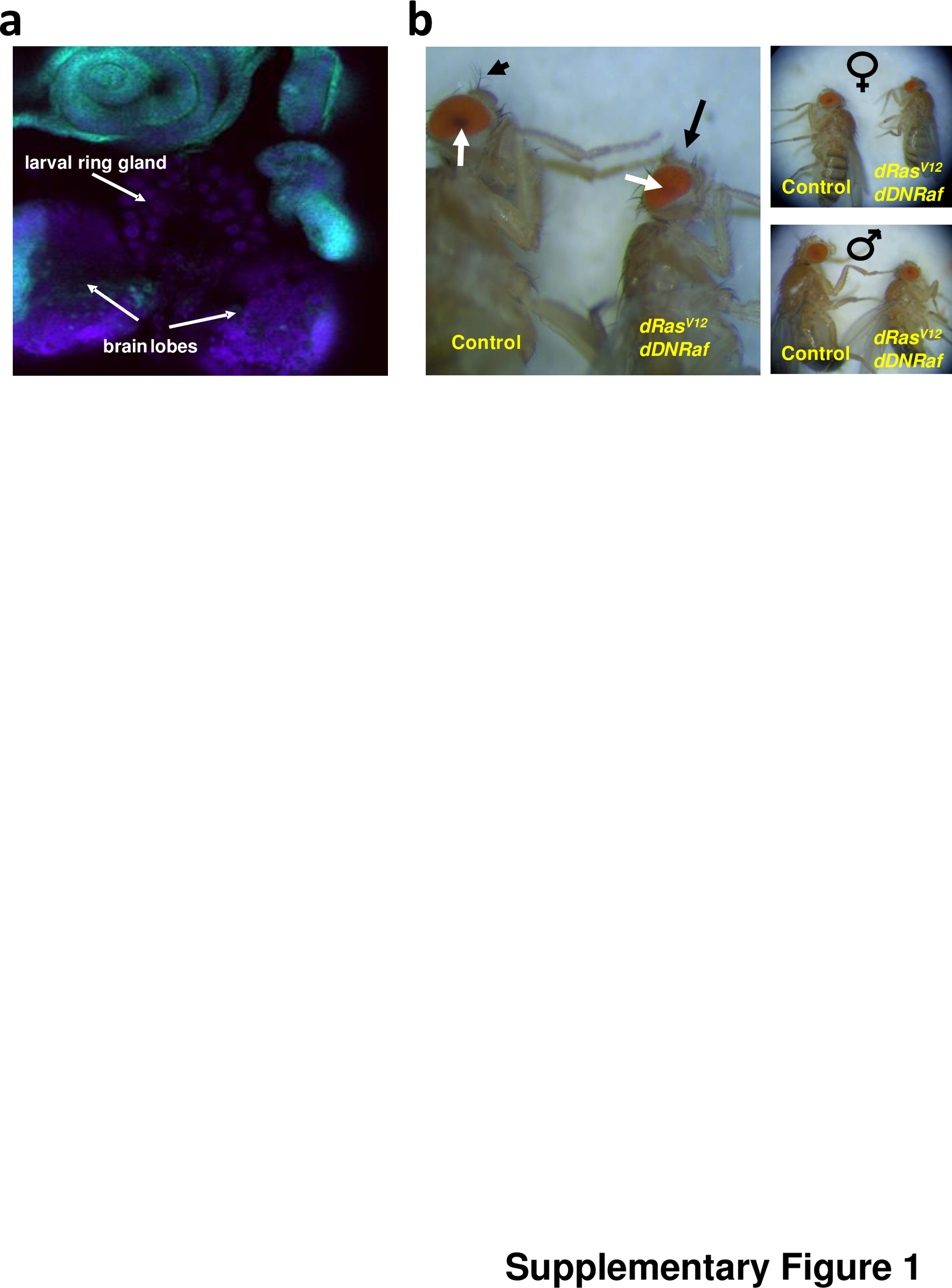

Supplement: S1 Fig — (a) eyeless-Flp does not drive expression of the GFP-transgene of the fly driver line in the larval ring gland, which has previously been shown to affect pupariation, confirming previous findings that defective pupal development is due to epithelial tumor formation. Magnification X 40. (b) Rescue of pupal lethality in both male and female dRasV12 bearing flies by co-expression of dominant negative (DN) dRaf (dRafDN), confirming that the observed phenotypes are not due to genetic load of the experimental line but due to dRasV12-induced overgrowth of the larval eye-antennal disc. Note that compared to wild type flies (left), dRasV12 dRafDN flies exhibited normal eye-morphology (white arrows) but still incomplete formation of the antenna (black arrows) and reduced body size. (TIF) [file pgen.1007688.s001.tif]

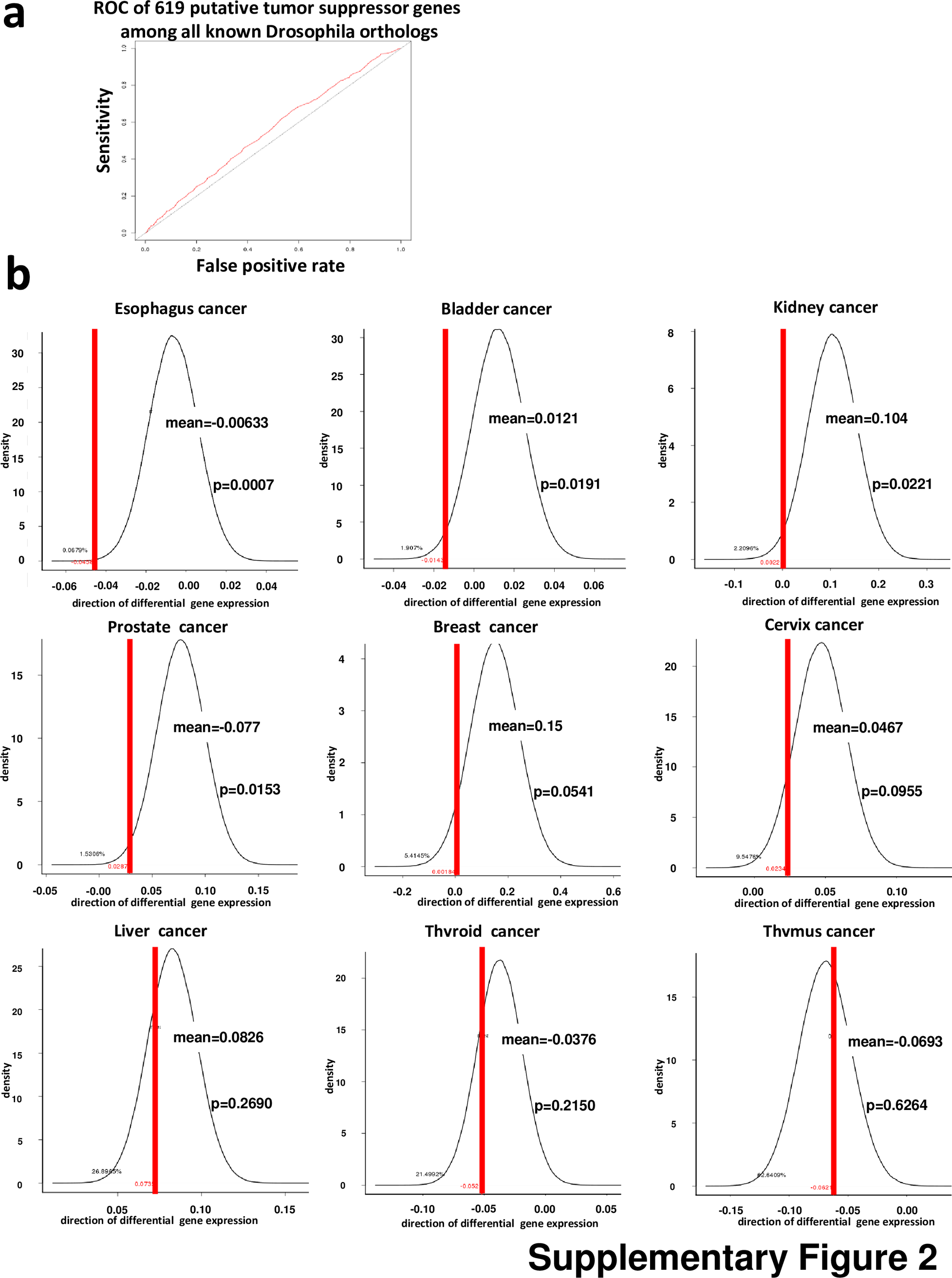

Supplement: S2 Fig — (a) Receiver Operator Characteristics (ROC) for the hit-list among the Drosophila orthologs and calculated Area Under the Curve (AUC). (b) Tumor suppressor oncogene scores (TSOS) to assess gene expression of our candidate hits in malignancies of the indicated tissues compared to the corresponding normal tissues for all human orthologs of our fly hits (red line) versus the median distribution of gene expression of randomly chosen gene sets (numbers of samplings are indicated for each tumor, n = 619 genes for microarray gene sets and n = 637 for the TCGA and GTEx RNAseq data sets). Only human orthologs of all tested fly genes (S1 Table) were included to avoid bias of comparison. Positive values represent a higher expression in tumors, a negative TSOS shows a higher expression in normal tissues on average. For data on gene expression in tumors and normal tissue see S3 Table. P values are indicated for each tumor type. (TIF) [file pgen.1007688.s002.tif]

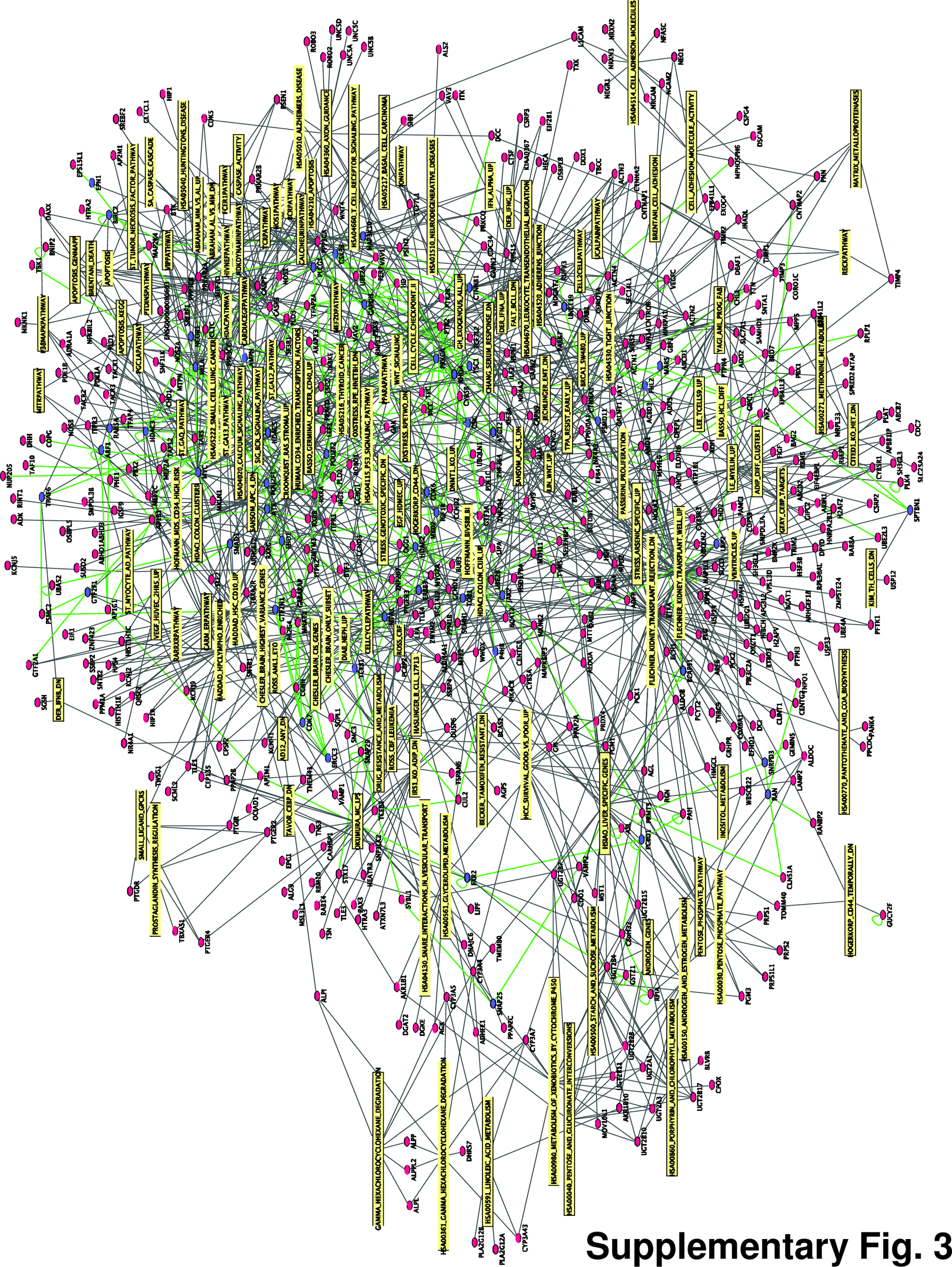

Supplement: S3 Fig — Shown are the significantly enriched C2 terms with a nominal p-value ≤0.05 (yellow) for human genes that are ortholog to primary fly RNAi hits (red) and first degree binding partners (blue). Only first degree binding partners were included that interact with at least 2 direct hits. The edges denote the protein assignments to C2 terms (grey) and the reported protein-protein interactions in BioGrid 8.0 are shown as green lines. For the entire data, see S8-4 and S8-5 Table. (TIF) [file pgen.1007688.s003.tif]

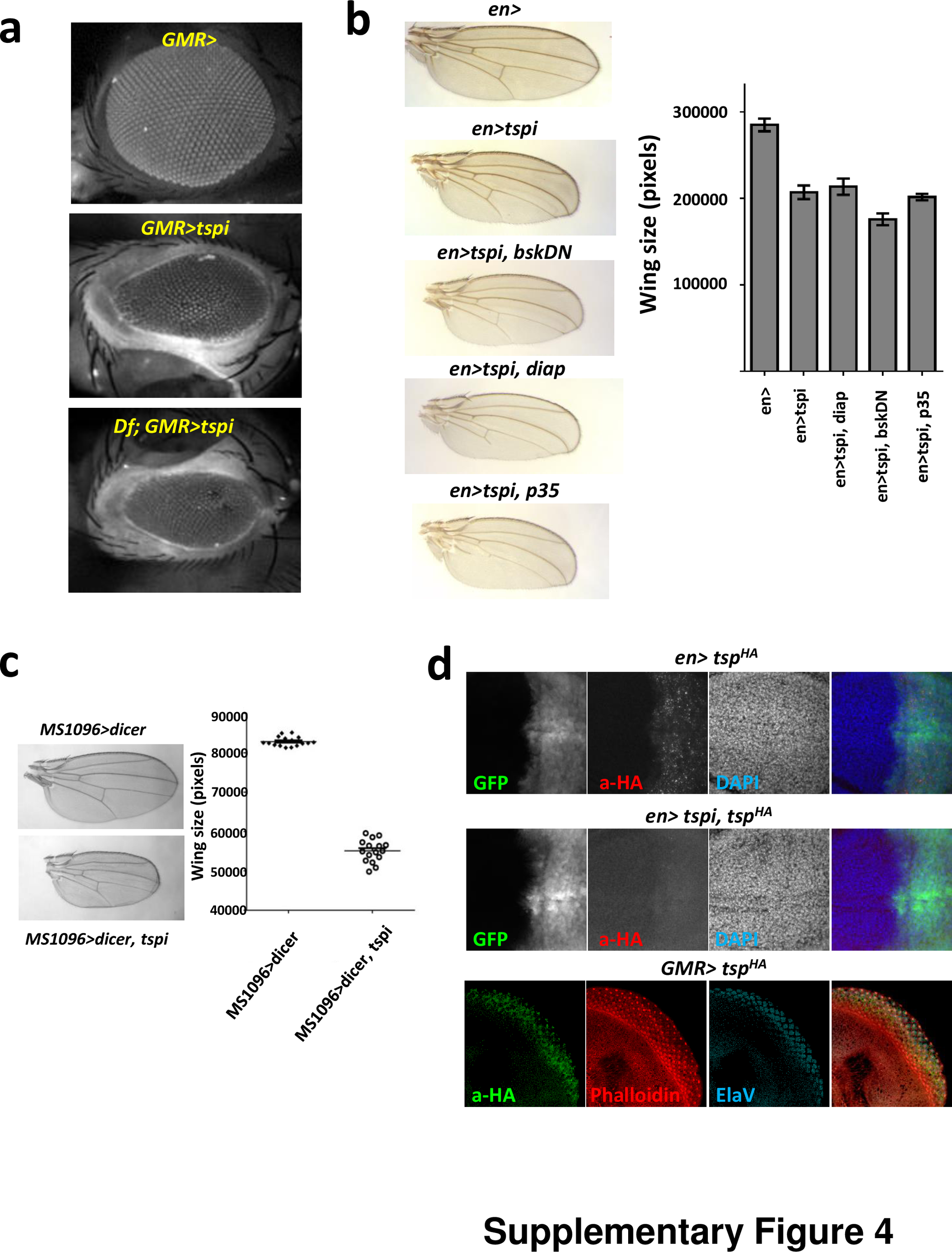

Supplement: S4 Fig — (a) The observed small rough phenotype of GMR >tspi flies is augmented by reducing endogenousTsp29Fb using a deficiency (Df; GMR>tspi), confirming the fidelity of the Tsp29Fb shRNAs. Flies were propagated at 25°C and photographed with a black & white CCD camera. (b) Expression of tspi via the engrailed (en) driver in the posterior region of the developing wing results in reduced wing size, which was not rescued by blocking apoptosis with expression of the dominant negative JNK transgene (bskDN), the Drosophila inhibitor of apoptosis Diap1, or the effector caspase inhibitor p35. Quantification of the wing sizes (pixels) is shown in the bar graph (+/- SEM, n>10 wings per sample). Flies were propagated at 29°C. (c) Expression of tspi via the MS1096 driver in the presence of dicer in the wing margins results in a reduced wing phenotype. Quantification of the wing sizes (pixels) is shown in the scatter plot (n>10 wings per sample). Flies were propagated at 29°C. (d) The efficacy of Tsp29Fb-RNAi is revealed by knockdown of HA expression of a HA-tagged Tsp29Fb (tspHA) transgene; Tsp29Fb-HA protein accumulated apically in wing as well as eye-antennal discs and co-expression of Tsp29Fb-RNAi substantially reduced Tsp29Fb protein levels, underscoring the efficacy of shRNA targeting and specificity of the Tsp29Fb knockdown-mediated effects. Top panels: Expression of a Tsp29Fb-HA transgene via the en driver in third instar larval wing discs stained with anti-HA, GFP and DAPI, shows apical accumulation of Tsp29Fb-HA in the posterior compartment (GFP+). Middle panels: Co-expression of tspi with the Tsp29Fb-HA transgene results in downregulation of HA staining, indicating that the RNAi targets Tsp29Fb. Lower panels: Driving Tsp29Fb-HA in the posterior region of the third instar larval eye disc using the GMR driver, stained with anti-HA, phalloidin (to detect F-actin) and anti-Elav (to detect differentiating neurons), shows that Tsp29Fb-HA is colocalized apically with F-actin. Flies [file pgen.1007688.s004.tif]

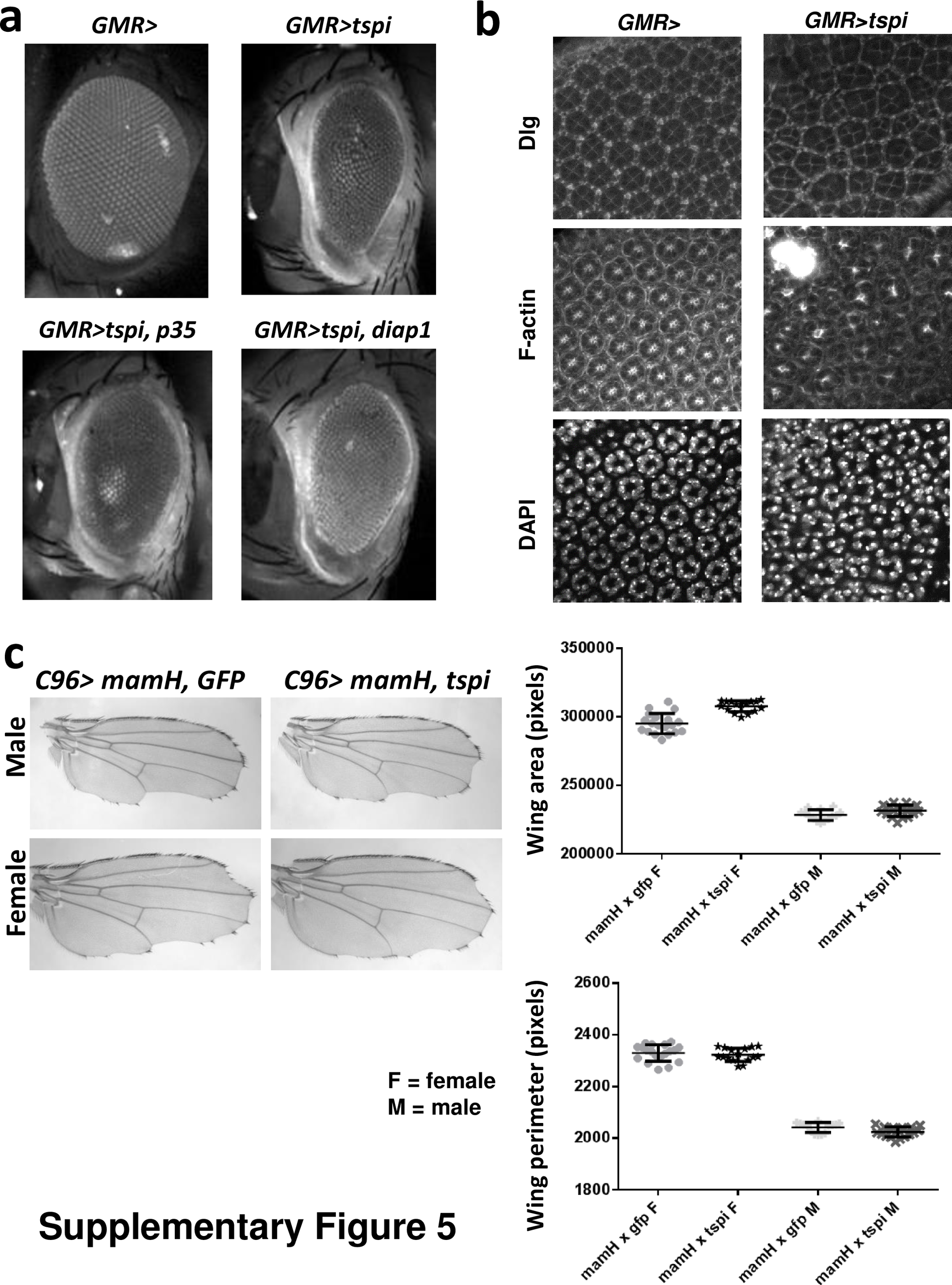

Supplement: S5 Fig — (a) The GMR>tspi reduced adult eye size is not rescued by co-expression of p35 or DIAP. Flies were propagated at 25°C and photographed with a black & white CCD camera. (b) Control (GMR) and GMR>tspi pupal retinas, stained for Dlg and F-actin (phalloidin) and DAPI to detect DNA. Knockdown of Tsp29Fb throughout the pupal retina results in disruption of the organized hexagonal array (bottom panel) relative to the wild type eyes (top panel), and Dlg and F-actin apical localization is disrupted. (c) Notched wing phenotype due to expression of dominant negative mastermind (mamH), to inhibit Notch signaling, in the wing margins via the C96 driver (left panels) is not modified by tspi expression. Right bar graphs indicate quantification of wing areas and the wing perimeters in pixels (+/- SEM, n>10 wings per sample) in both male (M) and female (F) flies with the indicated genotypes. No significant differences in C96>mamH wing area or perimeter measurements were observed upon tspi expression relative to the GFP control. Flies were propagated at 29°C. (TIF) [file pgen.1007688.s005.tif]

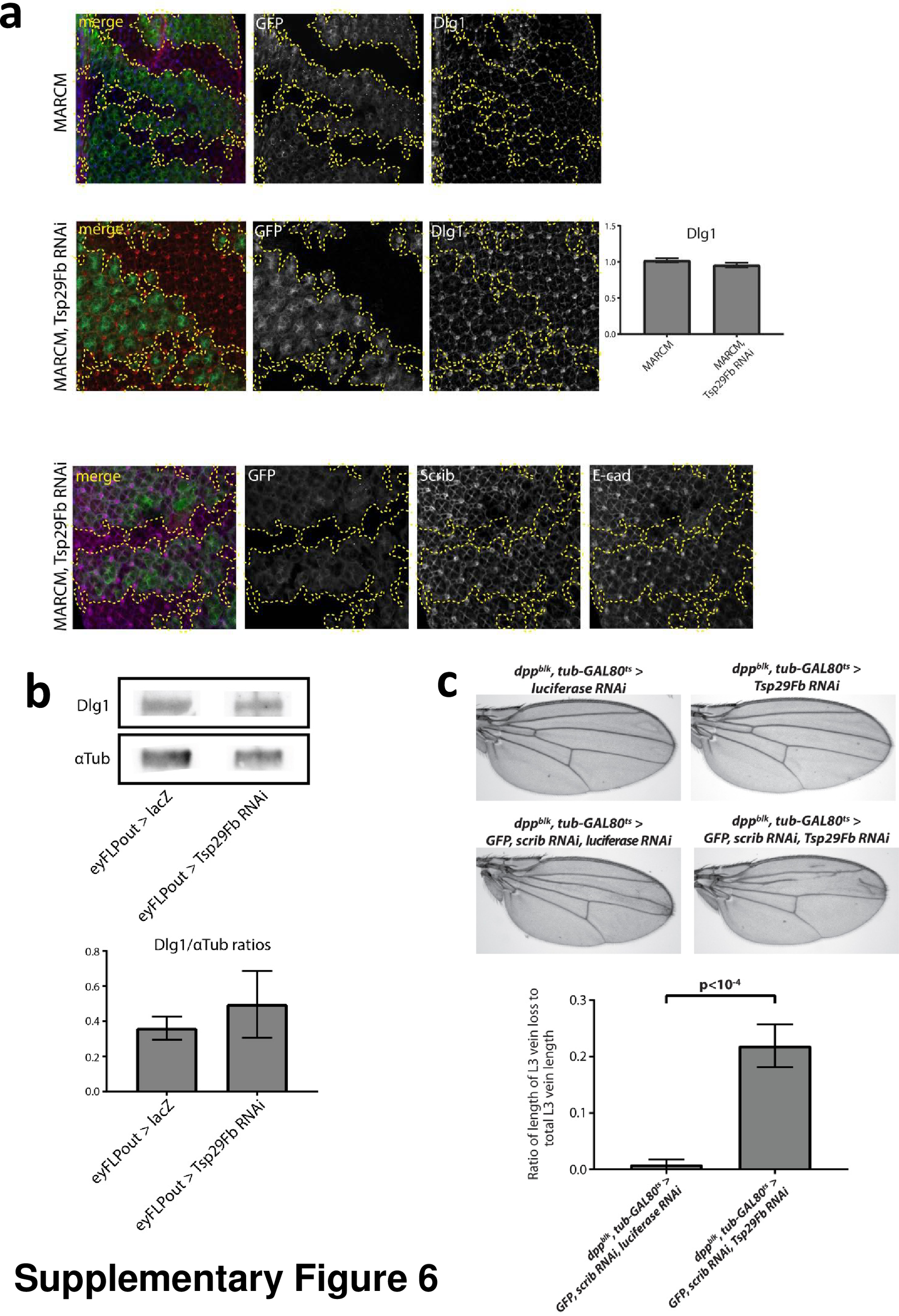

Supplement: S6 Fig — (a) Planar confocal images of mosaic pupal retinas from control (top panel), and Tsp29Fb-RNAi expressing clones marked with GFP (middle and bottom panel) stained with anti-Dlg (top and middle panels) or anti-Scrib and anti-E-cad (bottom panel), showing that Tsp29Fb affects Dlg localization but not Scrib or E-cad at junctions. Quantification of Dlg abundance in the Tsp29Fb-RNAi clones versus wild-type clones indicates that Dlg overall abundance is not significantly affected. (b) Western blot analysis of Dlg protein abundance relative to Tubulin in eyFLP-out larval eye-antennal discs, showing that Dlg protein abundance is not significantly affected by Tsp29Fb depletion. A representative Western blot is shown probed with anti-Dlg and anti-Tub and the graph is the quantification of 4 independent experiments and 2 technical replicates for each experiment. (c) Genetic interaction between scrib and Tsp29Fb in the adult wing from adult females. Expression of scrib-RNAi via dppBLK-GAL4 in the region between wing veins L3 and L4, results in reduced wing size, reduced area between wing veins L3 and L4 and breaks in wing vein L3 (bottom left) relative to the luciferase-RNAi control (top left), whilst expression of Tsp29Fb-RNAi has no effect (top right). Co-expression of Tsp29Fb-RNAi with scrib-RNAi results in enhancement of the scrib knock-down phenotype, resulting in an increase in the breaks in wing vein L3 (bottom right, quantified in the graph), indicating that Tsp29Fb genetically interacts with scrib in epithelial tissue development. For all experiments, crosses were propagated at 29°C. (TIF) [file pgen.1007688.s006.tif]

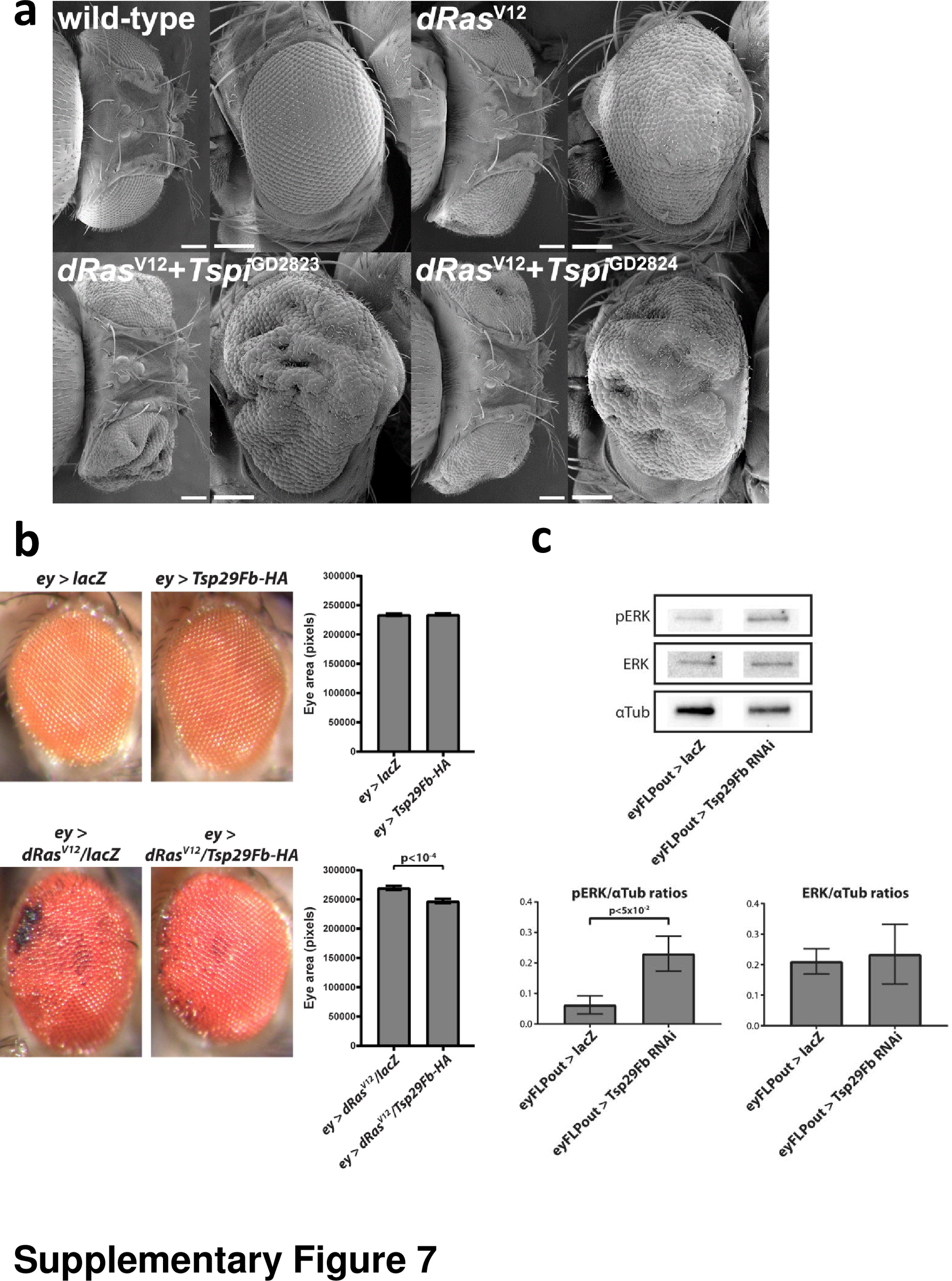

Supplement: S7 Fig — (a) Scanning electron micrographs of top or side views of adult female eyes of the indicated genotypes, showing that expression of a second Tsp29Fb-RNAi line (GD2823, bottom left) enhances the ey>dRasV12 overgrown eye phenotype (top right) similarly to GD2824 (bottom right). (b) Images of adult female eyes of the indicated genotypes, showing that expression of UAS-Tsp29Fb-HA (bottom right) suppresses the overgrown eye phenotype of the ey>dRasV12 lacZ control (bottom left) whereas expression of UAS-Tsp29Fb-HA alone (top right) has no effect relative to the ey>lacZ control (top left). (c) Western blot analysis of pERK, total-ERK protein abundance relative to Tubulin in eyFLP-out larval eye-antennal discs, showing that Tsp29Fb depletion results in a significant increase in pERK but is not significantly affect total-ERK relative to Tubulin. A representative Western blot is shown probed with anti-pERK, anti-total-ERK and anti-Tub and the graph is the quantification of 2 independent experiments and 2 technical replicates for each experiment. For all experiments, crosses were propagated at 29°C. (TIF) [file pgen.1007688.s007.tif]
