# Supplementary material for: A genome-wide Drosophila epithelial tumorigenesis screen identifies Tetraspanin 29Fb as an evolutionarily conserved suppressor of Ras-driven cancer
Source: PLoS Genet. 2018 Oct 16;14(10):e1007688. doi: 10.1371/journal.pgen.1007688 (PMC6203380; doi:10.1371/journal.pgen.1007688)
Supplement: S1 Data File — (a) mRNA expression analysis of 80, 55 or 25 genes correlations with patient survival and KRASG12 mutant status in pancreatic, lung or colon cancer. mRNA expression of the human orthologs of the validated 80 and 55 cancer candidate genes is significantly associated with survival of pancreas cancer patients, but not in lung or colon cancer patients. Data were obtained using K-means clustering on TCGA patient cohorts. P values (log rank test) and total numbers of patients with either low (black lines) or high (red lines) expression of the entire 80 candidate gene set are indicated. Low mRNA expression level of the 80, 55 cancer genes are significantly associated with the KRASG12 mutational status in human pancreas cancer but not in lung or colon cancer. The KRASG12 mutational status is shown for the low and high mRNA expression gene sets. None = no mutation; G12 = KRASG12 mutation; other = mutations in RAS other than KRASG12). Expression heat maps and KRAS mutations are also shown. (b) mRNA expression analysis of 80, 55 or 25 genes correlations with patient survival and combined Ras activating mutations (G12, G13, Q61) in KRAS, HaRAS and NRAS in pancreatic, lung or colon cancer. No significant correlation was observed in lung or colon cancer. Expression heat maps and RAS mutations are shown. (c) Summary table of the specific RAS mutations in patient cohorts are shown. (PDF) [file pgen.1007688.s008.pdf]

(a) KRAS-G12
Lung

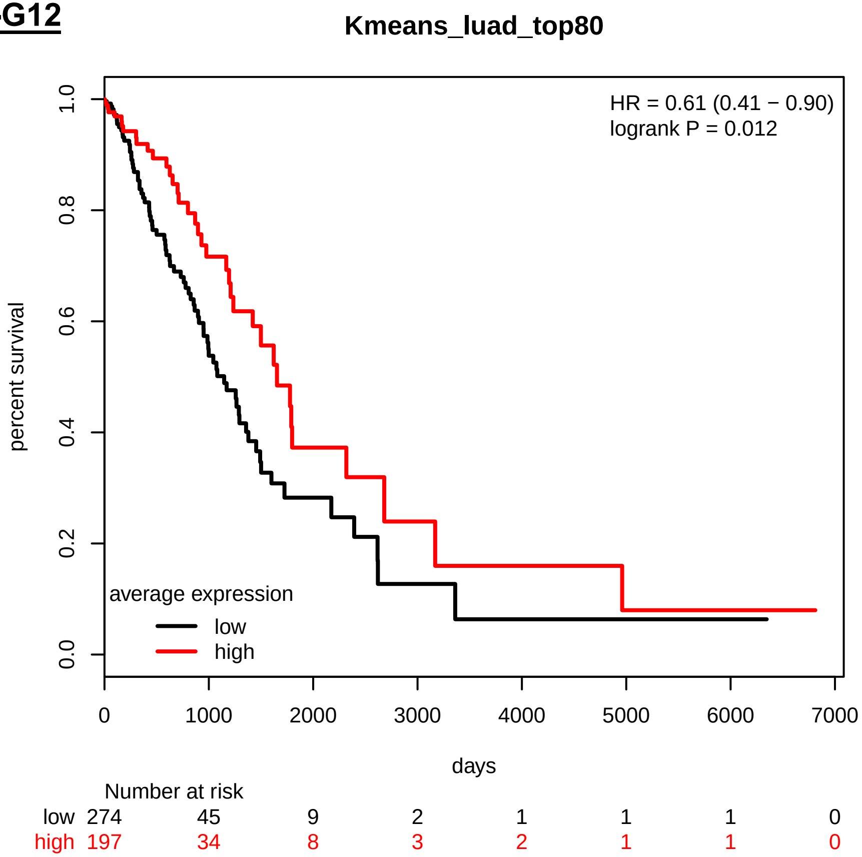

	KRAS_G12	none/other
low	65	209
high	67	130
pval_less	0.009622	
pval_greater	0.9946	

Kmeans_luad_top55

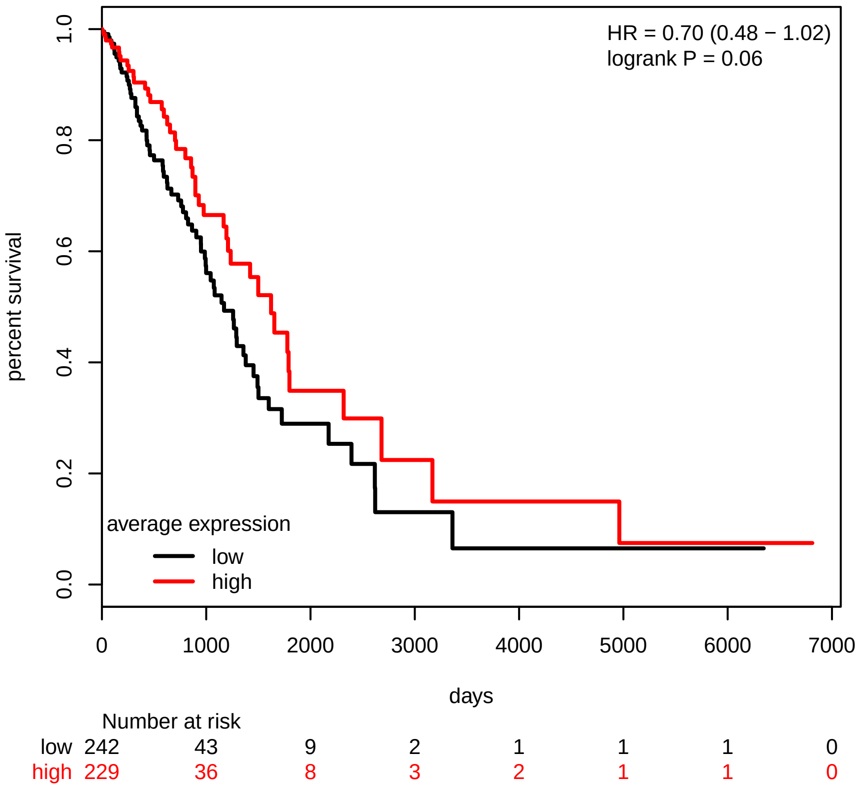

	KRAS_G12	none/other
low	56	186
high	76	153
pval_less	0.01002	
pval_greater	0.9943	

Kmeans_luad_top25

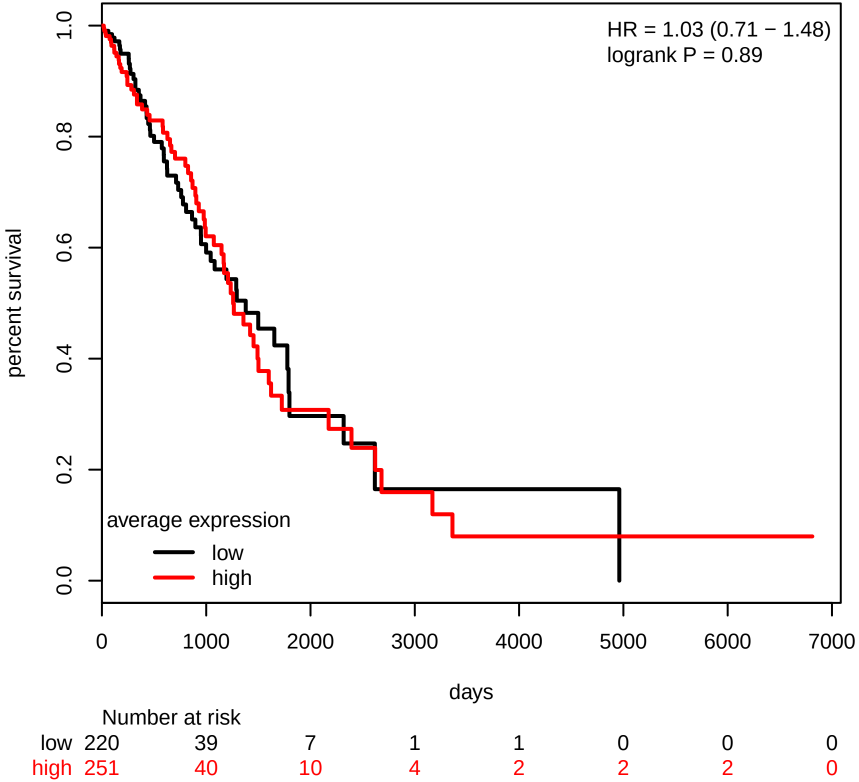

	KRAS_G12	none/other
low	67	153
high	65	186
pval_less	0.8852	
pval_greater	0.1596	

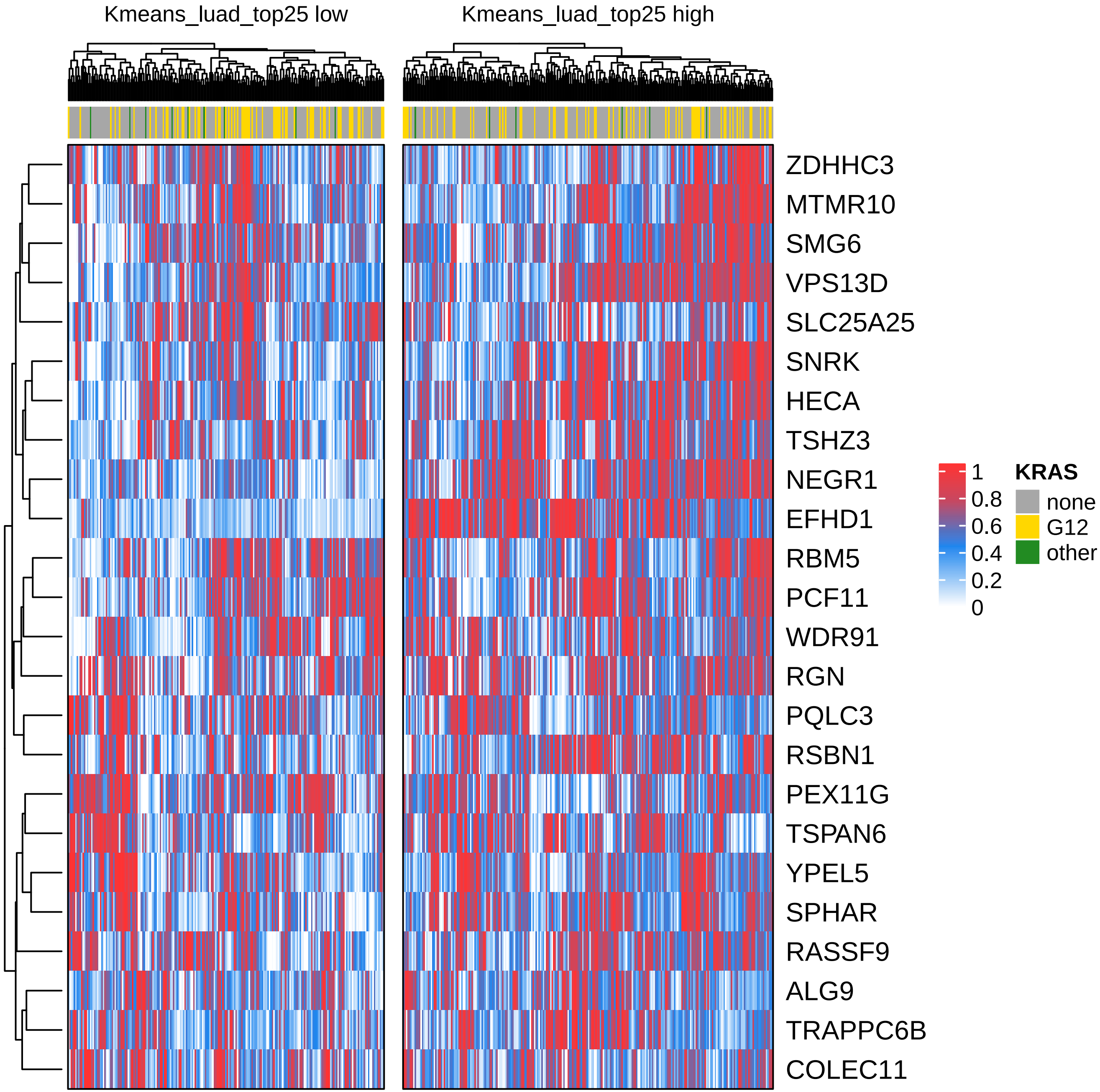
Kmeans_luad_top25 low
Kmeans_luad_top25 high
ZDHHC3
MTMR10
SMG6
VPS13D
SLC25A25
SNRK
HECA
TSHZ3
NEGR1
EFHD1
RBM5
PCF11
WDR91
RGN
PQLC3
RSBN1
PEX11G
TSPAN6
YPEL5
SPHAR
RASSF9
ALG9
TRAPPC6B
COLEC11
1
0.8
0.6
0.4
0.2
0
KRAS
none
G12
other

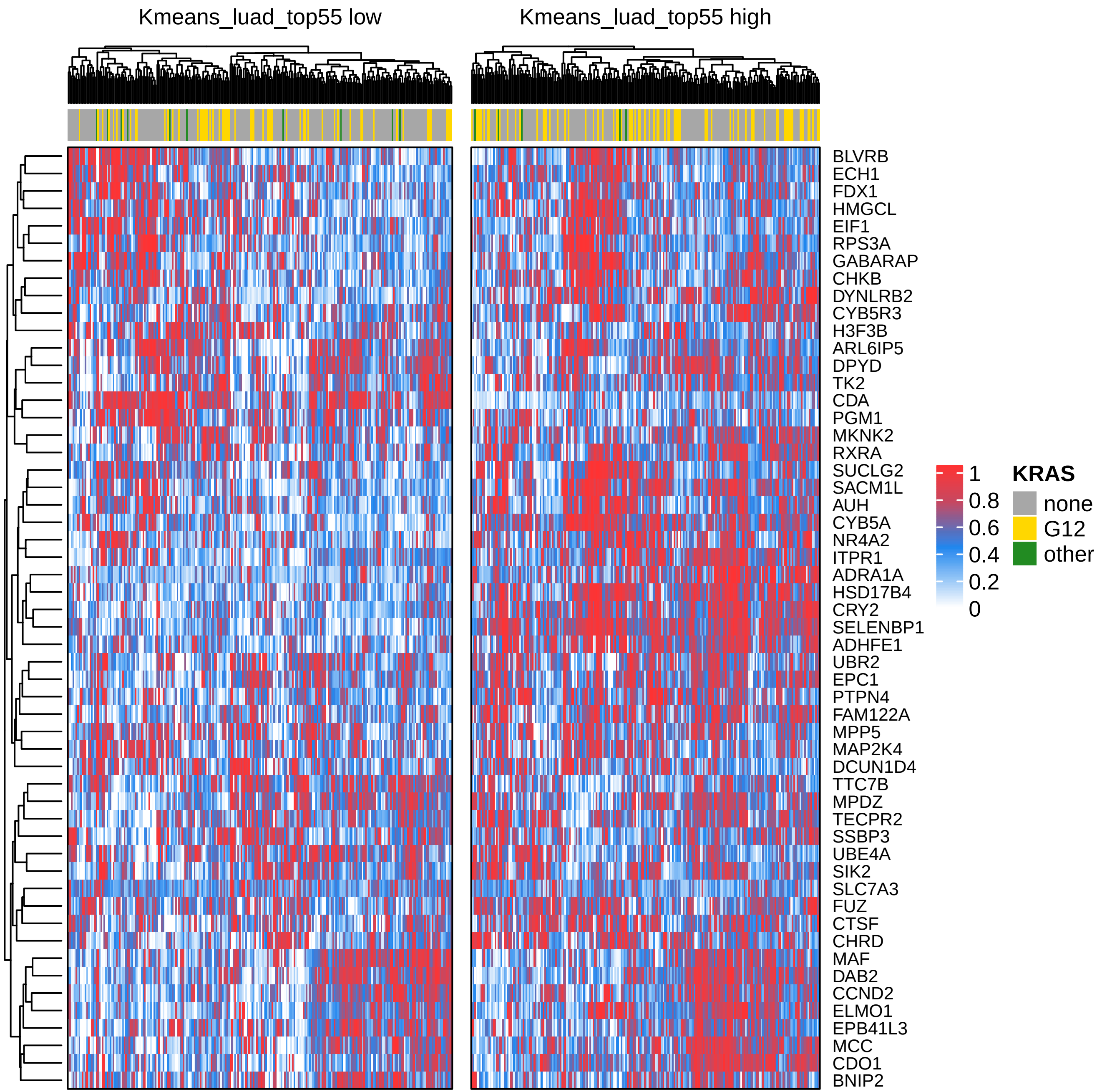
Kmeans_luad_top55 low
Kmeans_luad_top55 high
BLVRB
ECH1
FDX1
HMGCL
EIF1
RPS3A
GABARAP
CHKB
DYNLRB2
CYB5R3
H3F3B
ARL6IP5
DPYD
TK2
CDA
PGM1
MKNK2
RXRA
SUCLG2
SACM1L
AUH
CYB5A
NR4A2
ITPR1
ADRA1A
HSD17B4
CRY2
SELENBP1
ADHFE1
UBR2
EPC1
PTPN4
FAM122A
MPP5
MAP2K4
DCUN1D4
TTC7B
MPDZ
TECPR2
SSBP3
UBE4A
SIK2
SLC7A3
FUZ
CTSF
CHRD
MAF
DAB2
CCND2
ELMO1
EPB41L3
MCC
CDO1
BNIP2
1
0.8
0.6
0.4
0.2
0
KRAS
none
G12
other

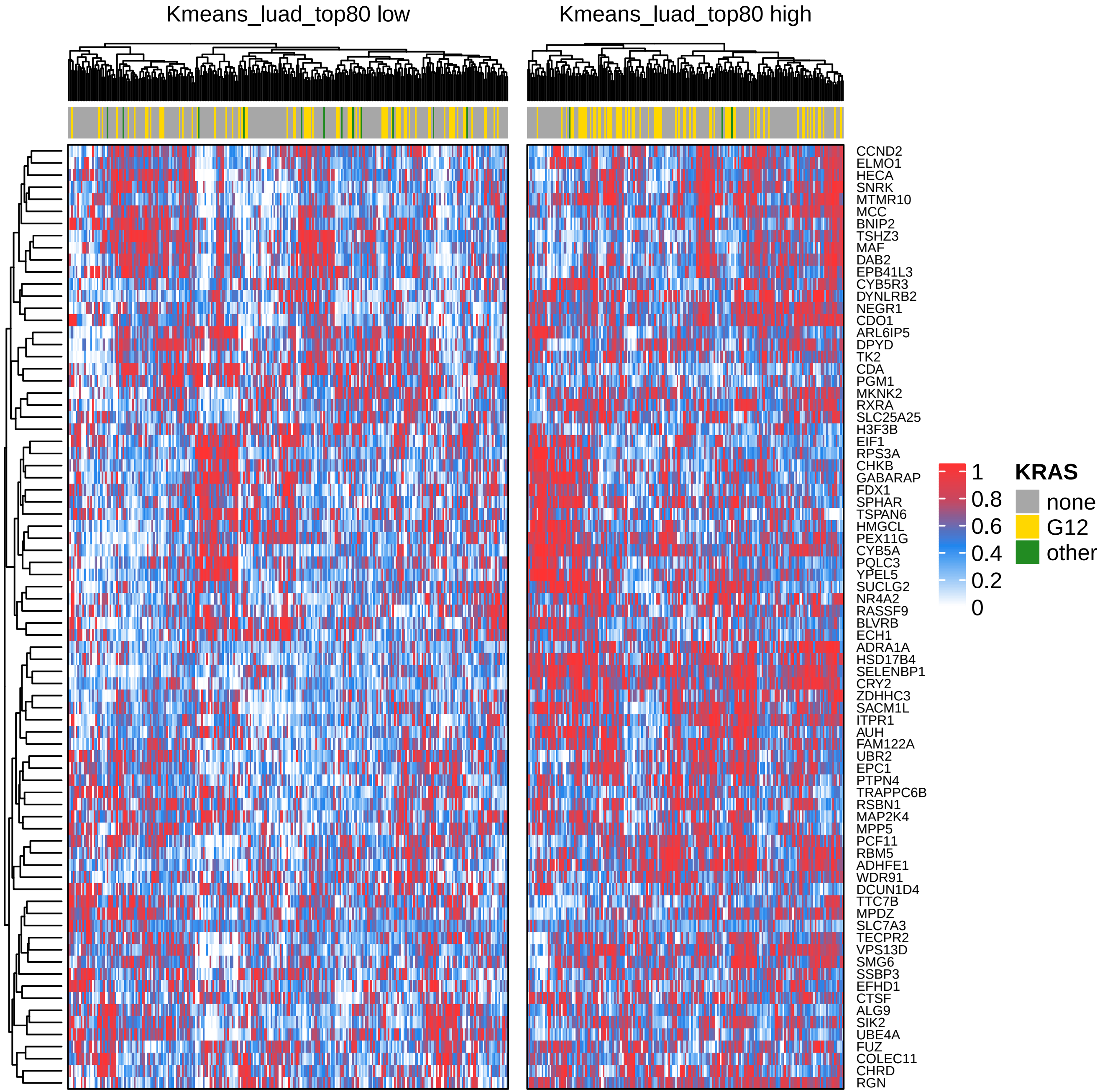
Kmeans_luad_top80 low
Kmeans_luad_top80 high
CCND2
ELMO1
HECA
SNRK
MTMR10
MCC
BNIP2
TSHZ3
MAF
DAB2
EPB41L3
CYB5R3
DYNLRB2
NEGR1
CDO1
ARL6IP5
DPYD
TK2
CDA
PGM1
MKNK2
RXRA
SLC25A25
H3F3B
EIF1
RPS3A
CHKB
GABARAP
FDX1
SPHAR
TSPAN6
HMGCL
PEX11G
CYB5A
PQLC3
YPEL5
SUCLG2
NR4A2
RASSF9
BLVRB
ECH1
ADRA1A
HSD17B4
SELENBP1
CRY2
ZDHHC3
SACM1L
ITPR1
AUH
FAM122A
UBR2
EPC1
PTPN4
TRAPPC6B
RSBN1
MAP2K4
MPP5
PCF11
RBM5
ADHFE1
WDR91
DCUN1D4
TTC7B
MPDZ
SLC7A3
TECPR2
VPS13D
SMG6
SSBP3
EFHD1
CTSF
ALG9
SIK2
UBE4A
FUZ
COLEC11
CHRD
RGN
1
0.8
0.6
0.4
0.2
0
KRAS
none
G12
other

KRAS-G12
Colon

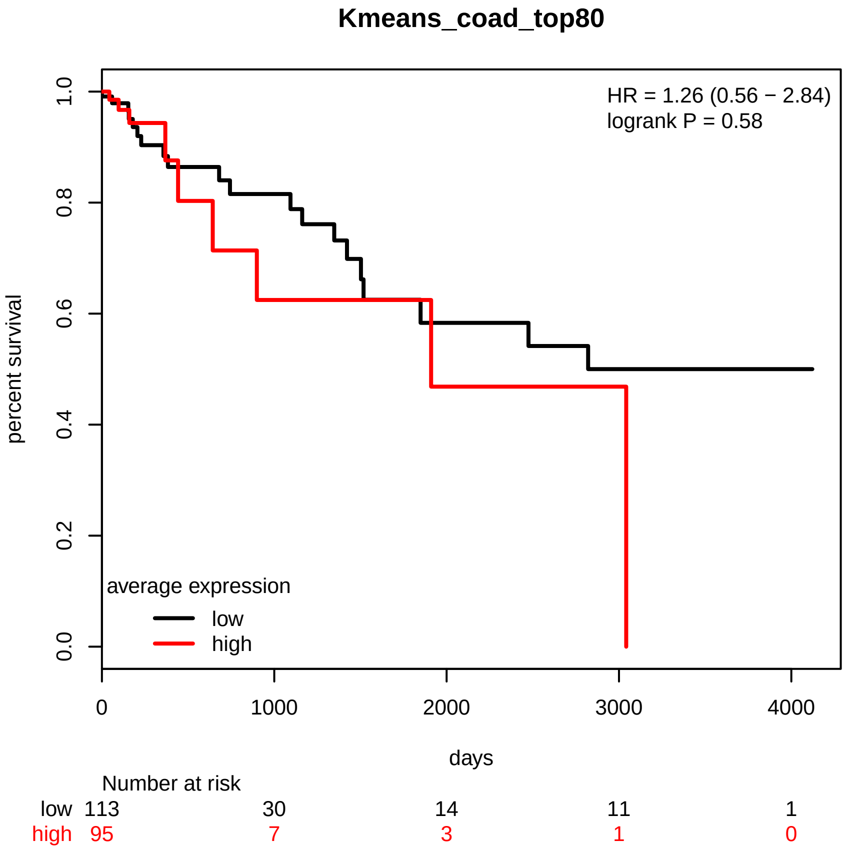

	KRAS_G12	none/other
low	31	82
high	24	71
pval_less	0.6948	
pval_greater	0.4233	

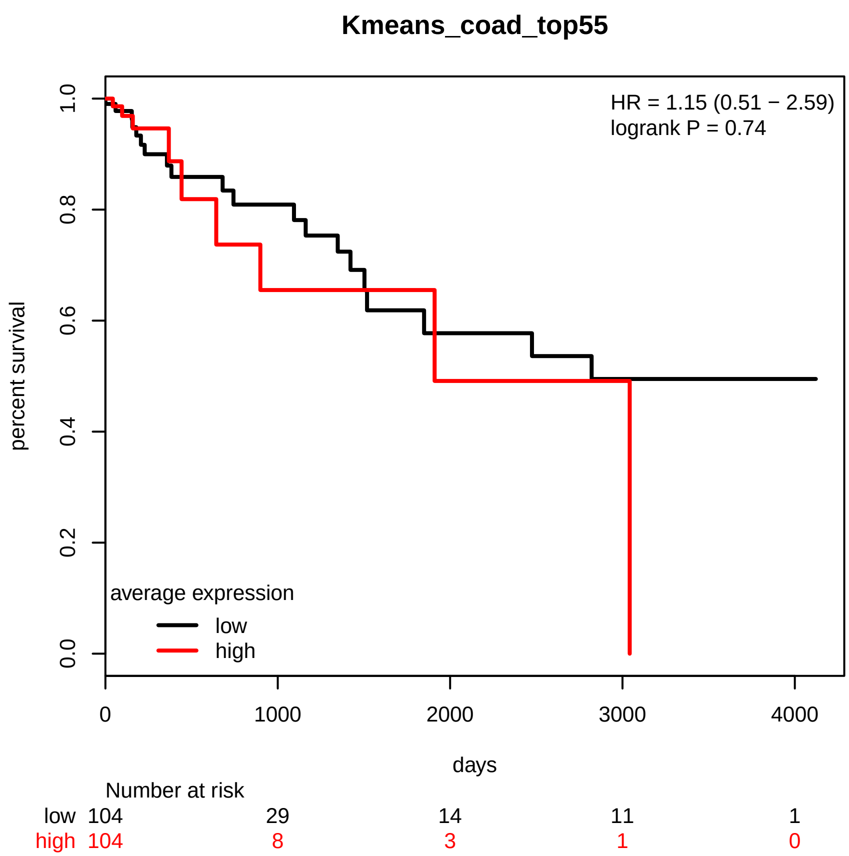

	KRAS_G12	none/other
low	29	75
high	26	78
pval_less	0.7352	
pval_greater	0.3767	

Kmeans_coad_top25

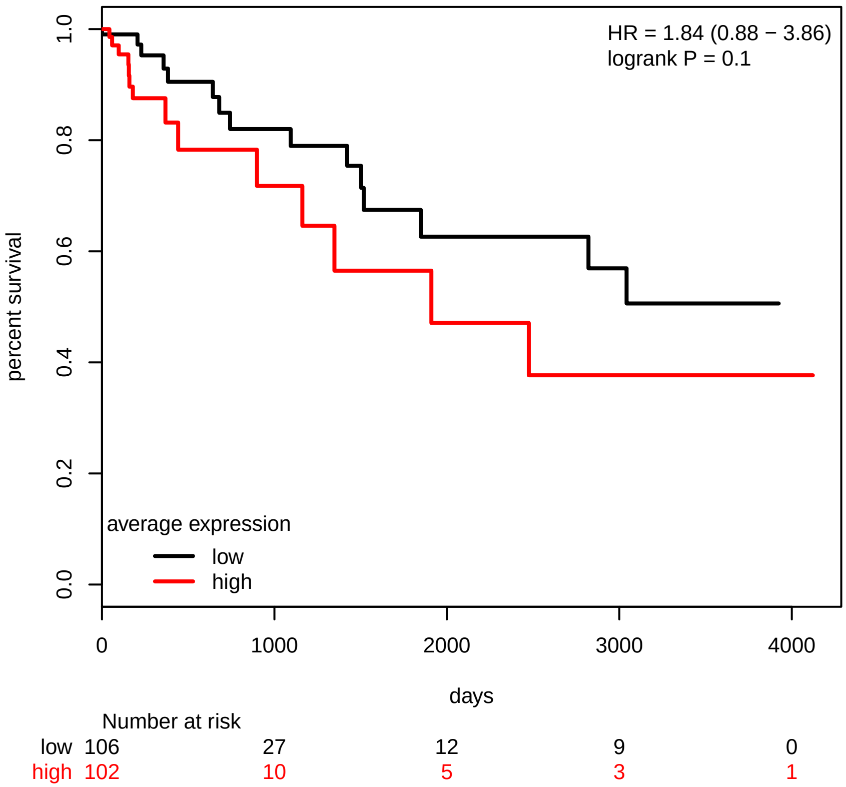

	KRAS_G12	none/other
low	32	74
high	23	79
pval_less	0.9204	
pval_greater	0.1374	

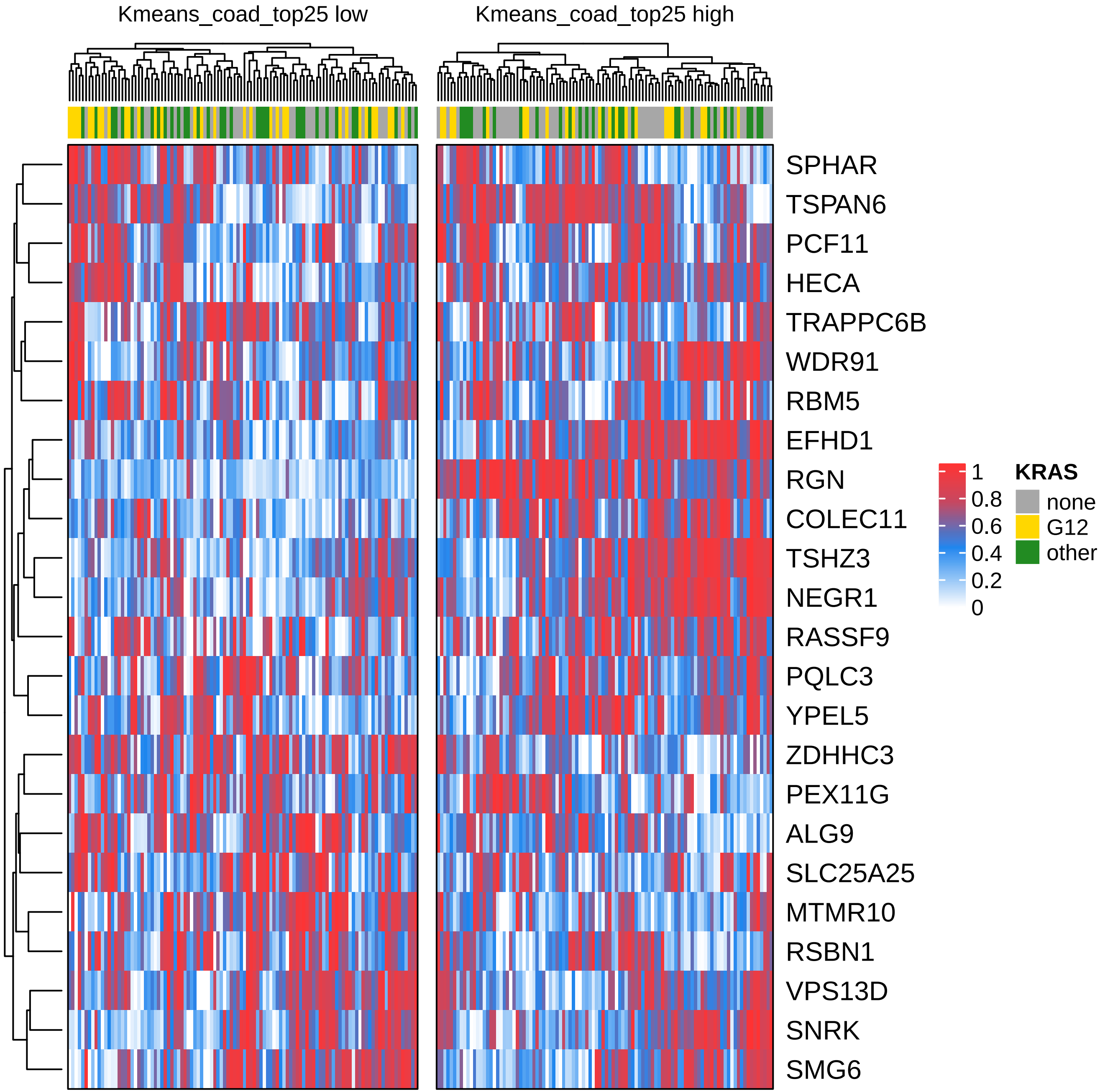
Kmeans_coad_top25 low
Kmeans_coad_top25 high
SPHAR
TSPAN6
PCF11
HECA
TRAPPC6B
WDR91
RBM5
EFHD1
RGN
COLEC11
TSHZ3
NEGR1
RASSF9
PQLC3
YPEL5
ZDHHC3
PEX11G
ALG9
SLC25A25
MTMR10
RSBN1
VPS13D
SNRK
SMG6
1
0.8
0.6
0.4
0.2
0
KRAS
none
G12
other

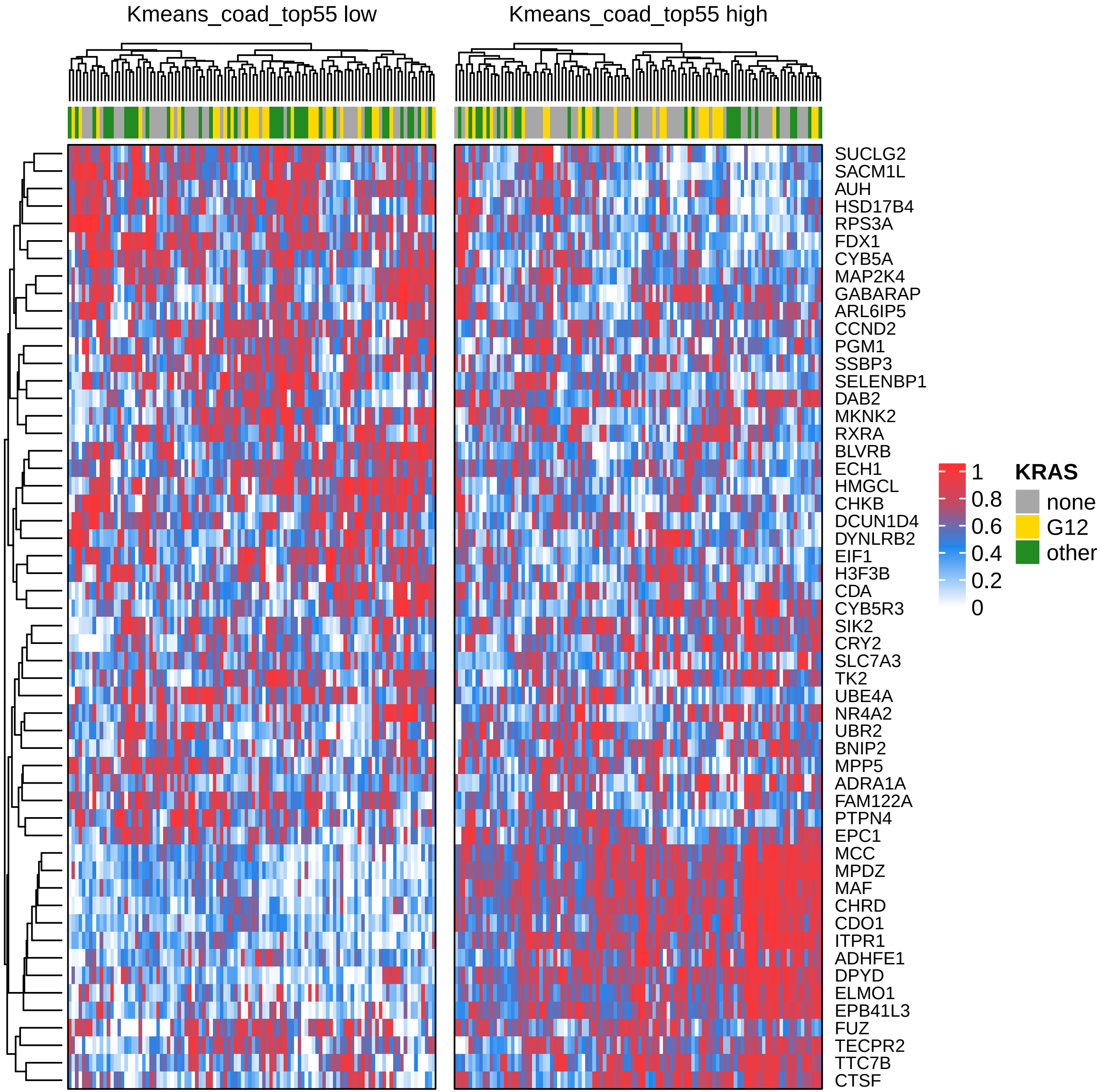
Kmeans_coad_top55 low
Kmeans_coad_top55 high
SUCLG2
SACM1L
AUH
HSD17B4
RPS3A
FDX1
CYB5A
MAP2K4
GABARAP
ARL6IP5
CCND2
PGM1
SSBP3
SELENBP1
DAB2
MKNK2
RXRA
BLVRB
ECH1
HMGCL
CHKB
DCUN1D4
DYNLRB2
EIF1
H3F3B
CDA
CYB5R3
SIK2
CRY2
SLC7A3
TK2
UBE4A
NR4A2
UBR2
BNIP2
MPP5
ADRA1A
FAM122A
PTPN4
EPC1
MCC
MPDZ
MAF
CHRD
CDO1
ITPR1
ADHFE1
DPYD
ELMO1
EPB41L3
FUZ
TECPR2
TTC7B
CTSF
1
0.8
0.6
0.4
0.2
0
KRAS
none
G12
other

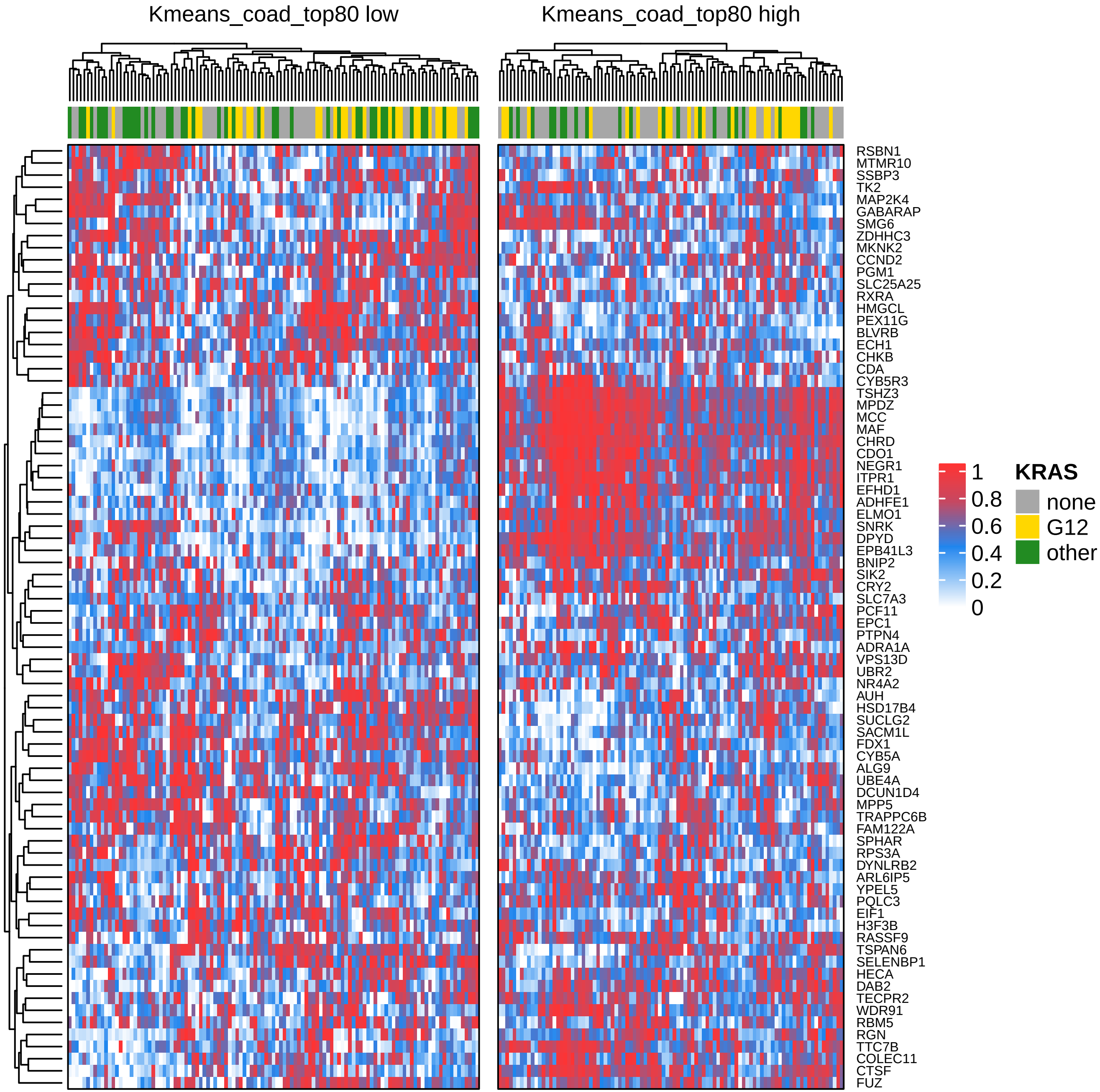

Kmeans_coad_top80 low
Kmeans_coad_top80 high
RSBN1
MTMR10
SSBP3
TK2
MAP2K4
GABARAP
SMG6
ZDHHC3
MKNK2
CCND2
PGM1
SLC25A25
RXRA
HMGCL
PEX11G
BLVRB
ECH1
CHKB
CDA
CYB5R3
TSHZ3
MPDZ
MCC
MAF
CHRD
CDO1
NEGR1
ITPR1
EFHD1
ADHFE1
ELMO1
SNRK
DPYD
EPB41L3
BNIP2
SIK2
CRY2
SLC7A3
PCF11
EPC1
PTPN4
ADRA1A
VPS13D
UBR2
NR4A2
AUH
HSD17B4
SUCLG2
SACM1L
FDX1
CYB5A
ALG9
UBE4A
DCUN1D4
MPP5
TRAPPC6B
FAM122A
SPHAR
RPS3A
DYNLRB2
ARL6IP5
YPEL5
PQLC3
EIF1
H3F3B
RASSF9
TSPAN6
SELENBP1
HECA
DAB2
TECPR2
WDR91
RBM5
RGN
TTC7B
COLEC11
CTSF
FUZ
1
0.8
0.6
0.4
0.2
0
KRAS
none
G12
other

KRAS-G12 Pancreatic

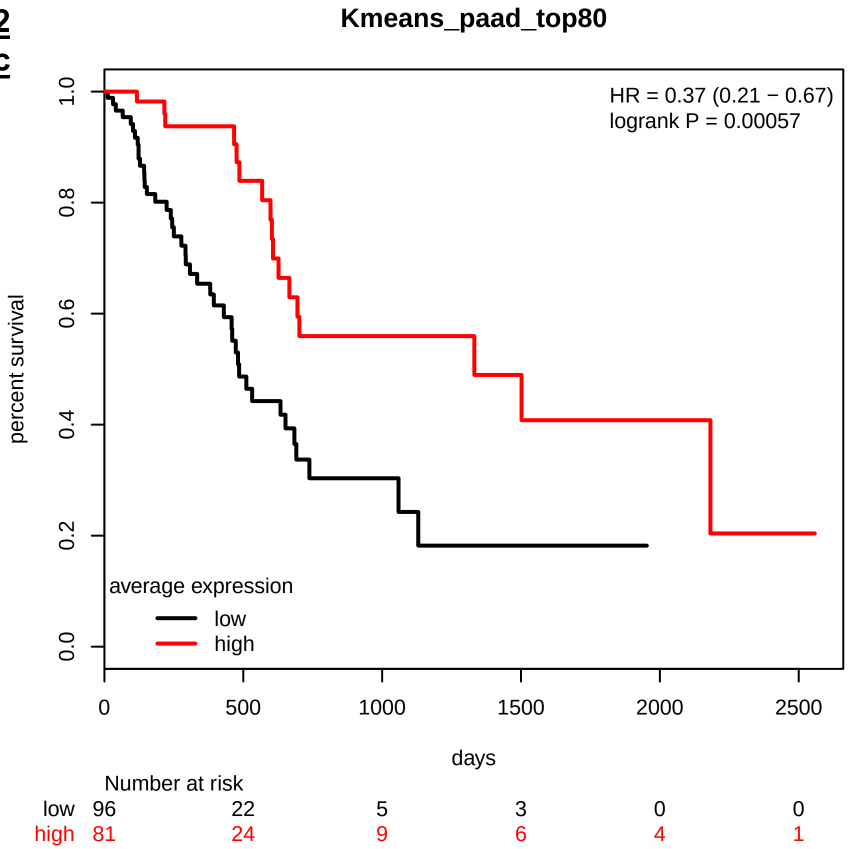

	KRAS_G12	none/other
low	79	17
high	45	36
pval_less	1	
pval_greater	9.94e−05	

Kmeans_paad_top55

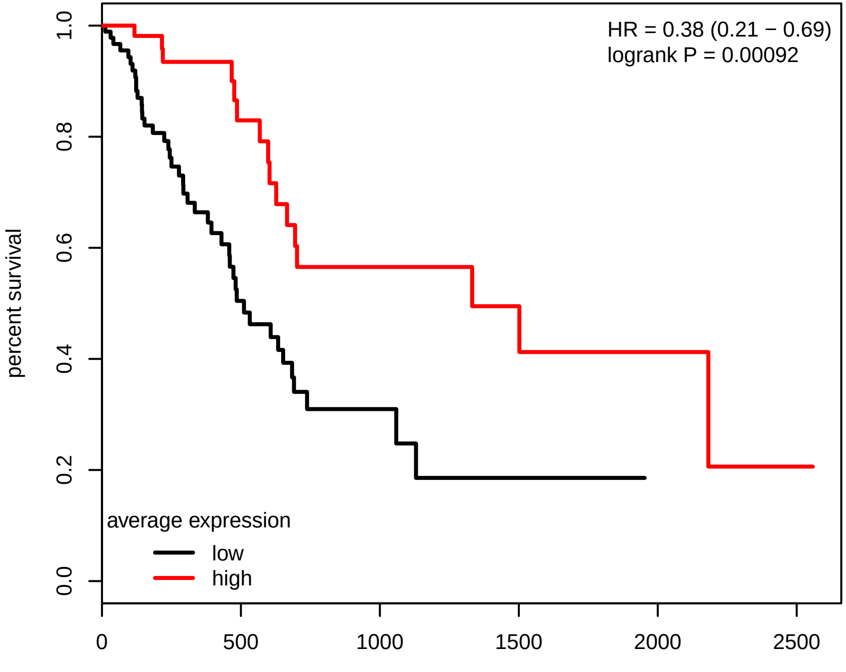

Number at risk

	0	500	1000	1500	2000	2500
low	99	24	5	3	0	0
high	78	22	9	6	4	1

	**KRAS_G12**	**none/other**
low	81	18
high	43	35
pval_less	1	
pval_greater	0.0001122	

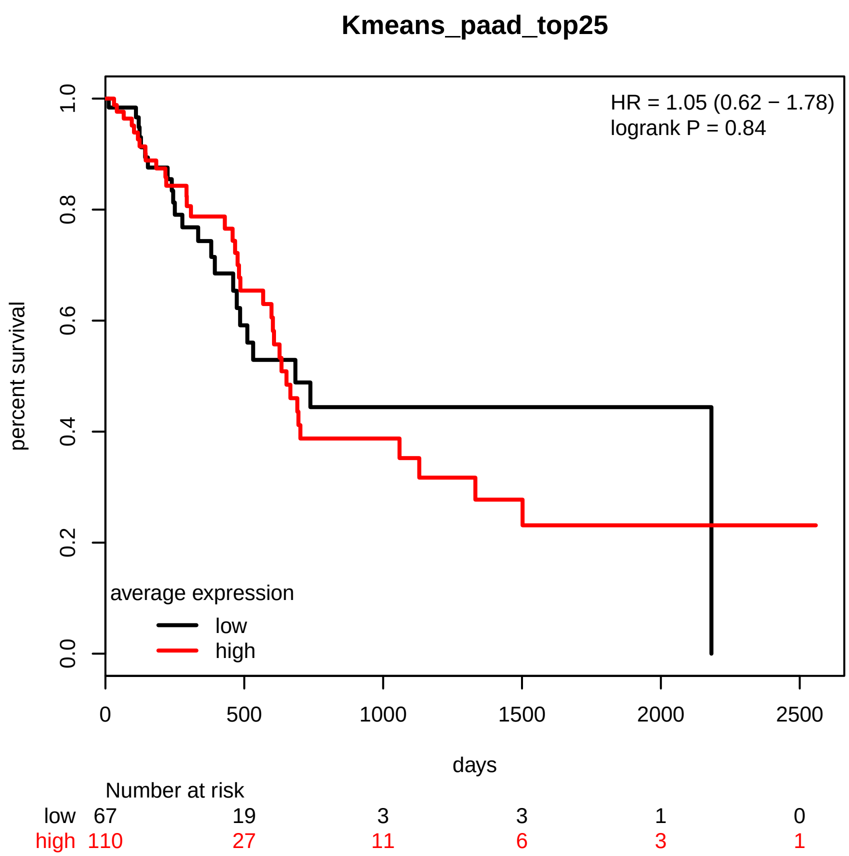

	KRAS_G12	none/other
low	53	14
high	71	39
pval_less	0.9878	
pval_greater	0.02862	

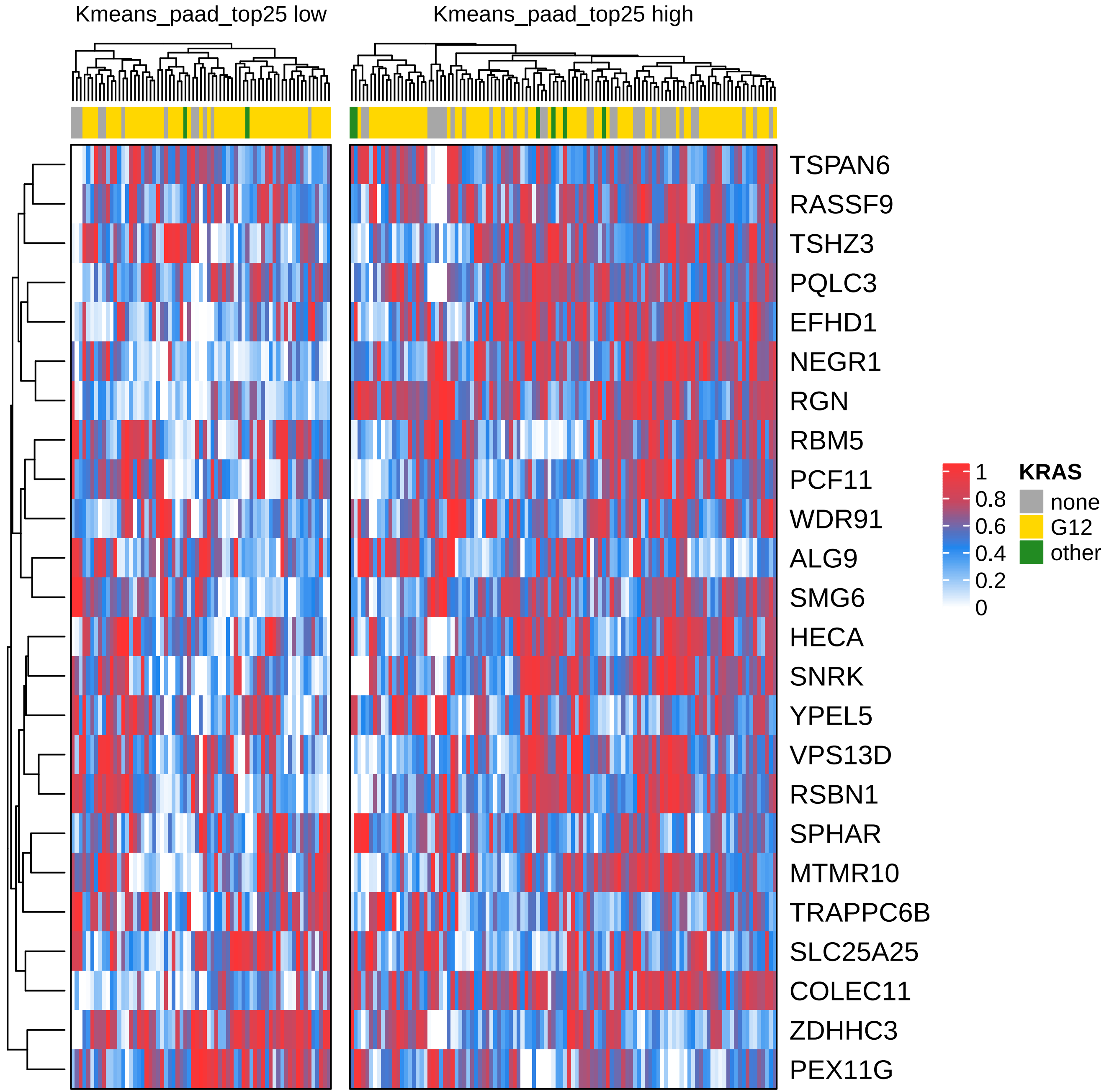
Kmeans_paad_top25 low
Kmeans_paad_top25 high
TSPAN6
RASSF9
TSHZ3
PQLC3
EFHD1
NEGR1
RGN
RBM5
PCF11
WDR91
ALG9
SMG6
HECA
SNRK
YPEL5
VPS13D
RSBN1
SPHAR
MTMR10
TRAPPC6B
SLC25A25
COLEC11
ZDHHC3
PEX11G
1
0.8
0.6
0.4
0.2
0
KRAS
none
G12
other

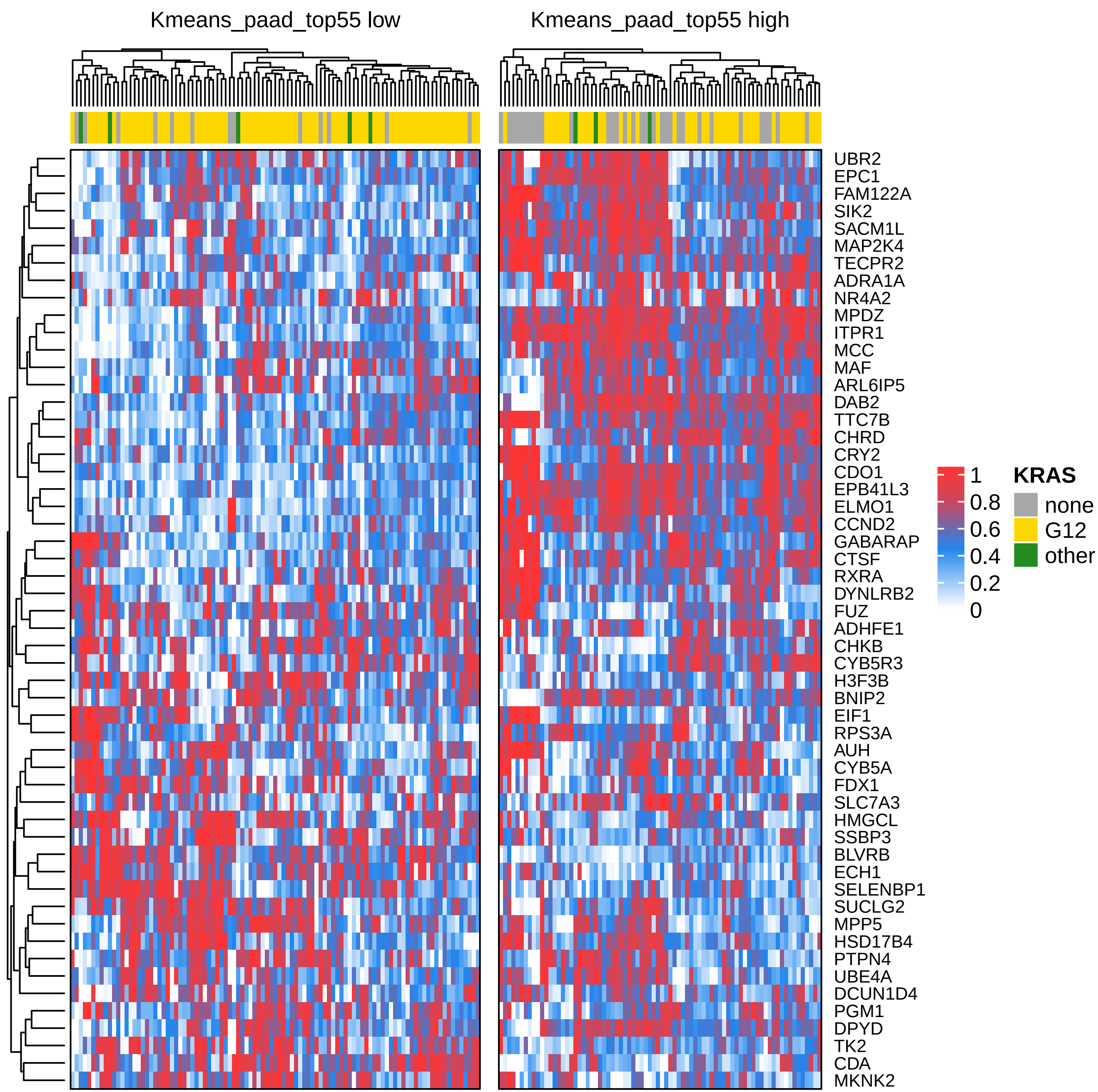

Kmeans_paad_top55 low
Kmeans_paad_top55 high
UBR2
EPC1
FAM122A
SIK2
SACM1L
MAP2K4
TECPR2
ADRA1A
NR4A2
MPDZ
ITPR1
MCC
MAF
ARL6IP5
DAB2
TTC7B
CHRD
CRY2
CDO1
EPB41L3
ELMO1
CCND2
GABARAP
CTSF
RXRA
DYNLRB2
FUZ
ADHFE1
CHKB
CYB5R3
H3F3B
BNIP2
EIF1
RPS3A
AUH
CYB5A
FDX1
SLC7A3
HMGCL
SSBP3
BLVRB
ECH1
SELENBP1
SUCLG2
MPP5
HSD17B4
PTPN4
UBE4A
DCUN1D4
PGM1
DPYD
TK2
CDA
MKNK2
1
0.8
0.6
0.4
0.2
0
KRAS
none
G12
other

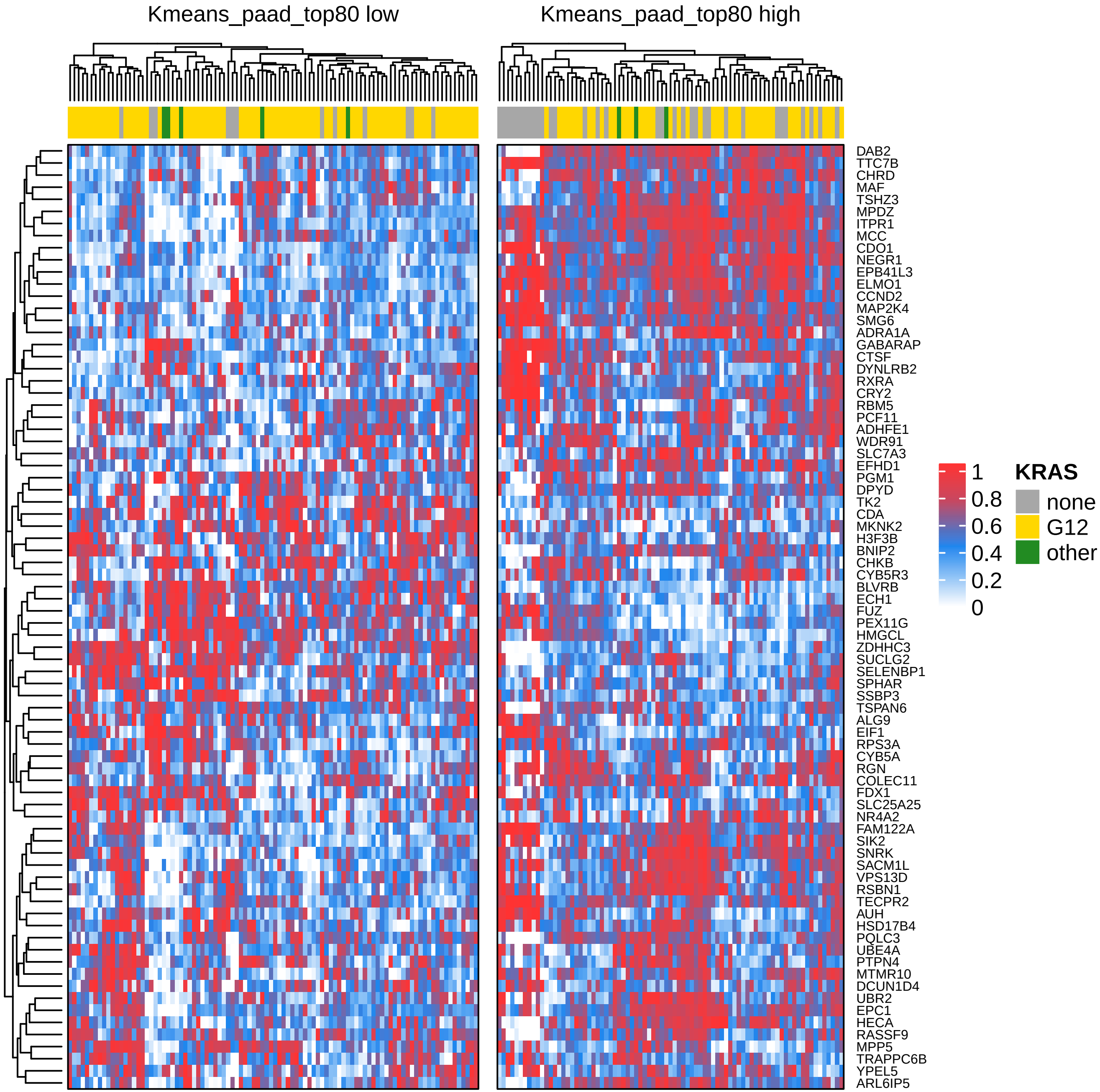
Kmeans_paad_top80 low
Kmeans_paad_top80 high
DAB2
TTC7B
CHRD
MAF
TSHZ3
MPDZ
ITPR1
MCC
CDO1
NEGR1
EPB41L3
ELMO1
CCND2
MAP2K4
SMG6
ADRA1A
GABARAP
CTSF
DYNLRB2
RXRA
CRY2
RBM5
PCF11
ADHFE1
WDR91
SLC7A3
EFHD1
PGM1
DPYD
TK2
CDA
MKNK2
H3F3B
BNIP2
CHKB
CYB5R3
BLVRB
ECH1
FUZ
PEX11G
HMGCL
ZDHHC3
SUCLG2
SELENBP1
SPHAR
SSBP3
TSPAN6
ALG9
EIF1
RPS3A
CYB5A
RGN
COLEC11
FDX1
SLC25A25
NR4A2
FAM122A
SIK2
SNRK
SACM1L
VPS13D
RSBN1
TECPR2
AUH
HSD17B4
PQLC3
UBE4A
PTPN4
MTMR10
DCUN1D4
UBR2
EPC1
HECA
RASSF9
MPP5
TRAPPC6B
YPEL5
ARL6IP5
1
0.8
0.6
0.4
0.2
0
KRAS
none
G12
other

(b)
All RAS
Lung

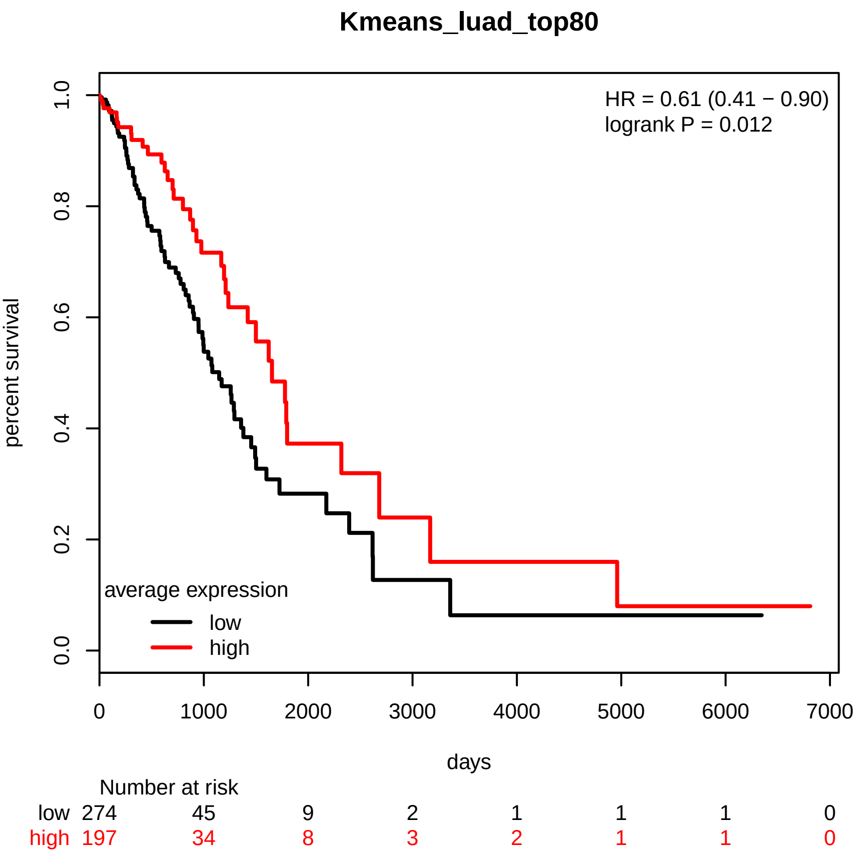

	12/13/61	none/other
low	68	206
high	67	130
pval_less	0.01934	
pval_greater	0.9885	

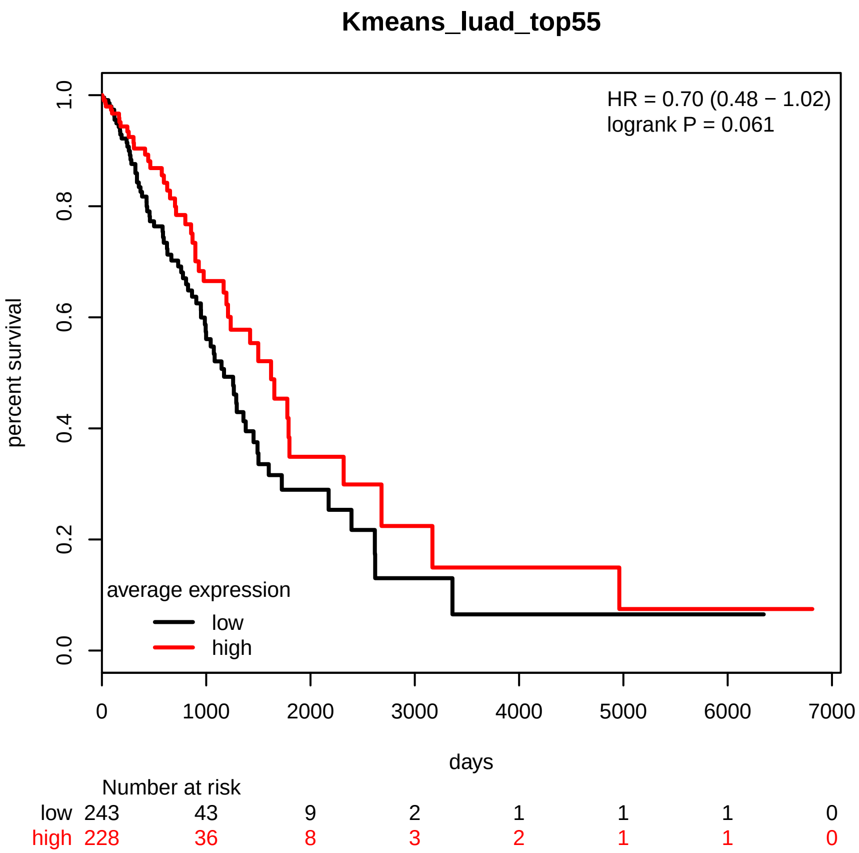

	12/13/61	none/other
low	60	183
high	75	153
pval_less	0.03102	
pval_greater	0.9808	

Kmeans_luad_top25

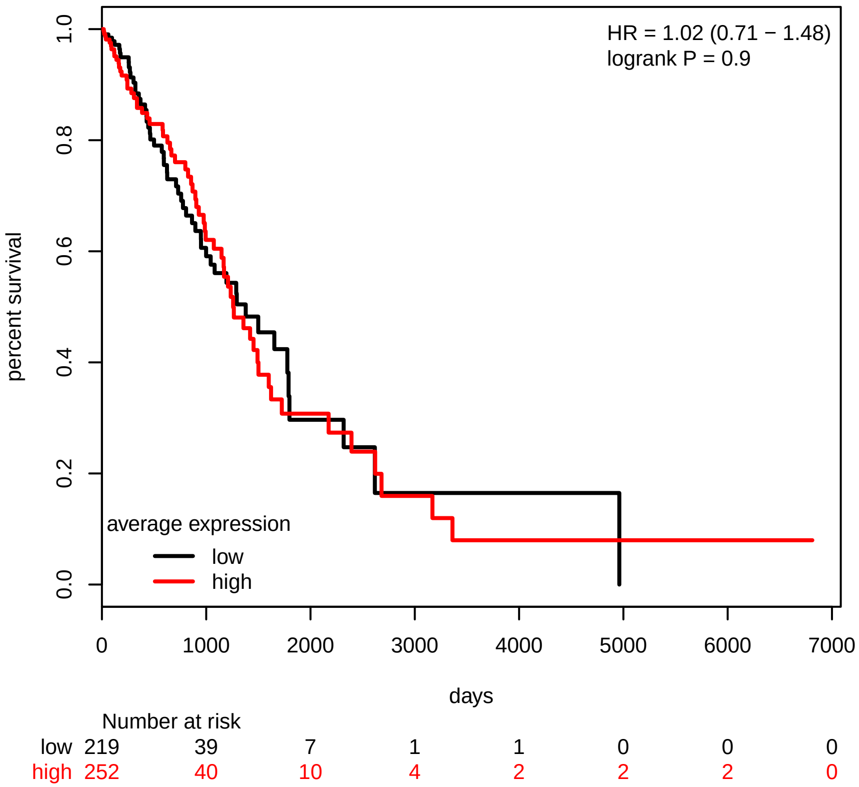

	12/13/61	none/other
low	68	151
high	67	185
pval_less	0.8791	
pval_greater	0.167	

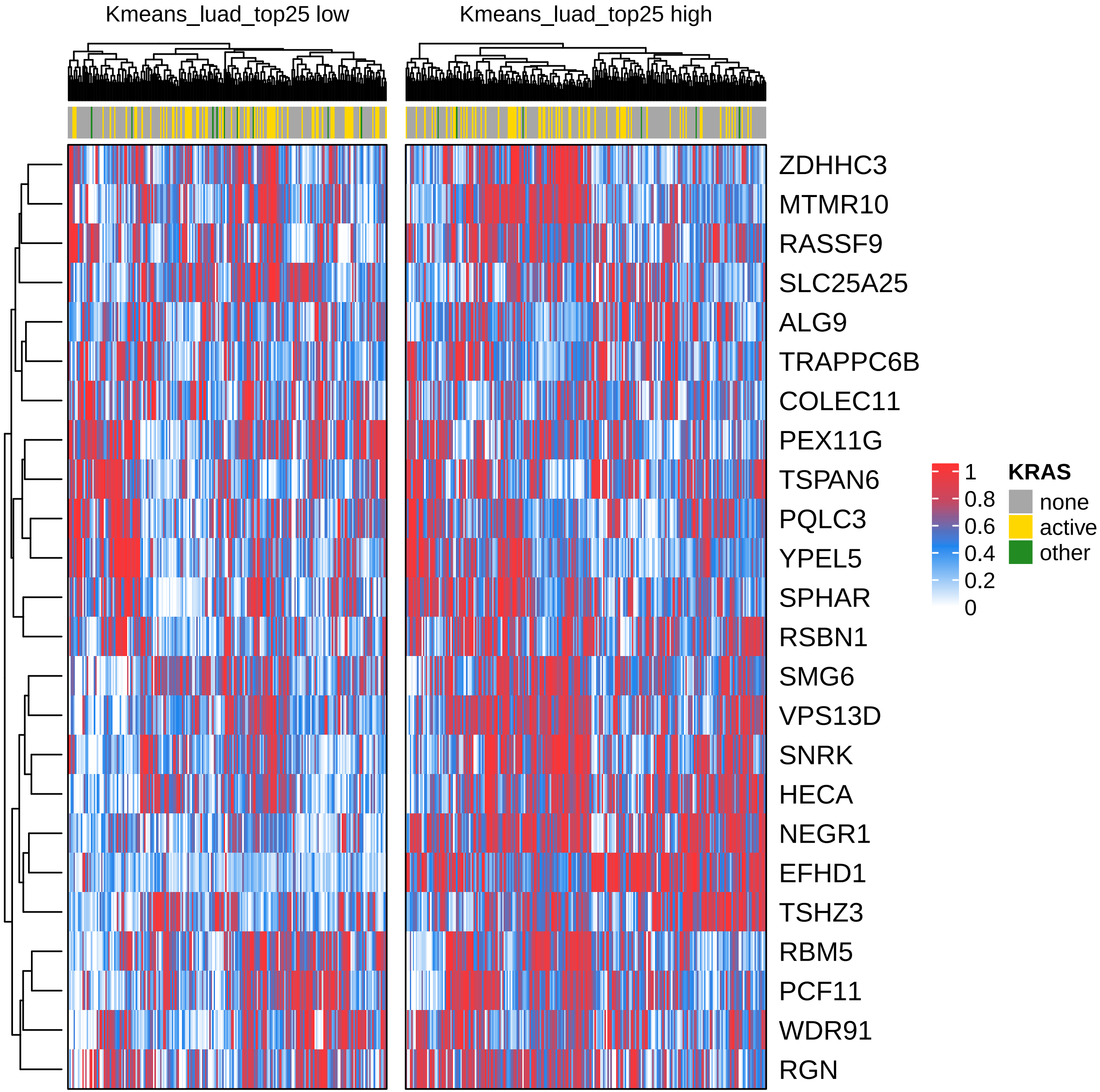
Kmeans_luad_top25 low
Kmeans_luad_top25 high
ZDHHC3
MTMR10
RASSF9
SLC25A25
ALG9
TRAPPC6B
COLEC11
PEX11G
TSPAN6
PQLC3
YPEL5
SPHAR
RSBN1
SMG6
VPS13D
SNRK
HECA
NEGR1
EFHD1
TSHZ3
RBM5
PCF11
WDR91
RGN
1
0.8
0.6
0.4
0.2
0
KRAS
none
active
other

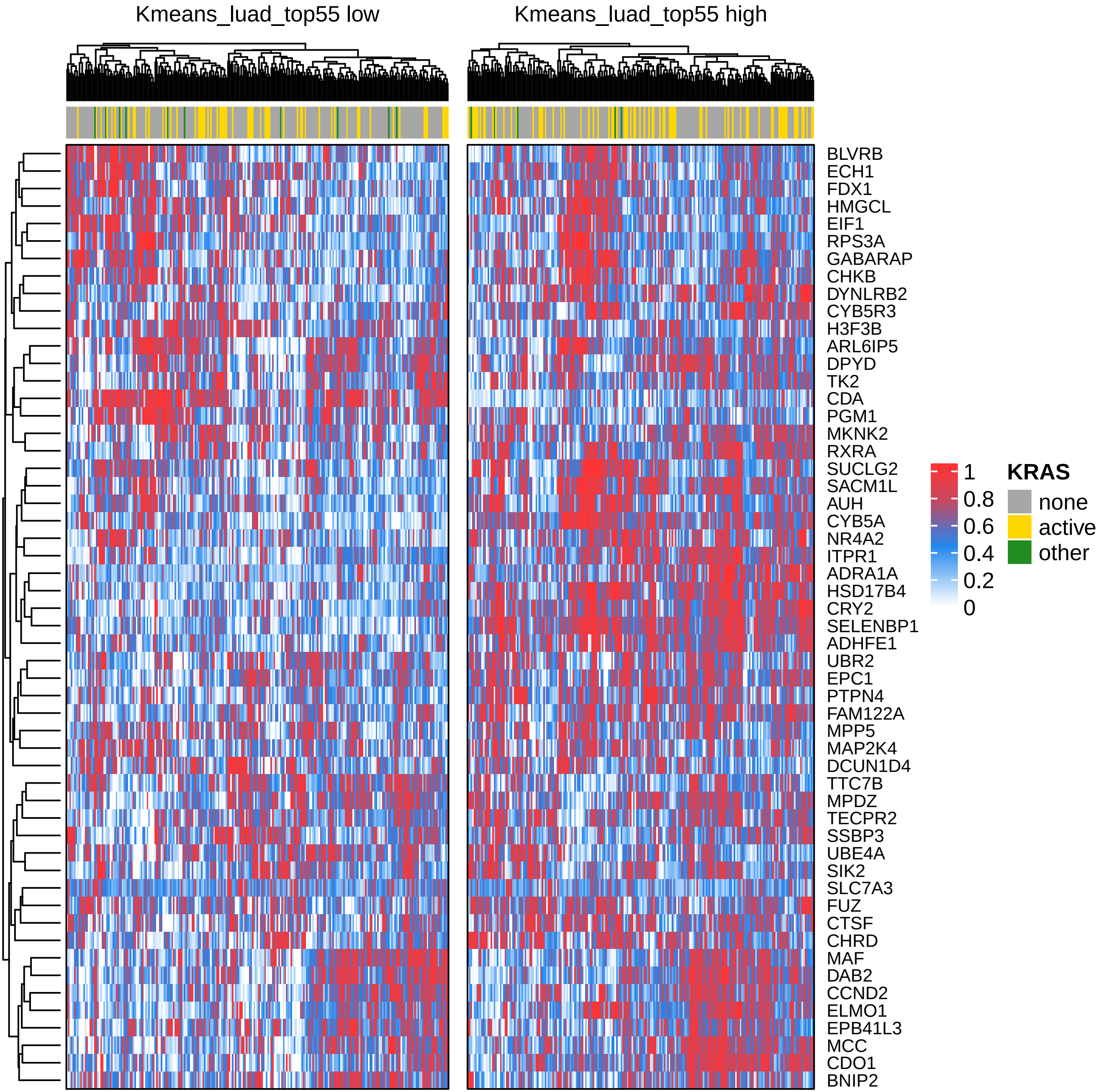
Kmeans_luad_top55 low
Kmeans_luad_top55 high
BLVRB
ECH1
FDX1
HMGCL
EIF1
RPS3A
GABARAP
CHKB
DYNLRB2
CYB5R3
H3F3B
ARL6IP5
DPYD
TK2
CDA
PGM1
MKNK2
RXRA
SUCLG2
SACM1L
AUH
CYB5A
NR4A2
ITPR1
ADRA1A
HSD17B4
CRY2
SELENBP1
ADHFE1
UBR2
EPC1
PTPN4
FAM122A
MPP5
MAP2K4
DCUN1D4
TTC7B
MPDZ
TECPR2
SSBP3
UBE4A
SIK2
SLC7A3
FUZ
CTSF
CHRD
MAF
DAB2
CCND2
ELMO1
EPB41L3
MCC
CDO1
BNIP2
1
0.8
0.6
0.4
0.2
0
KRAS
none
active
other

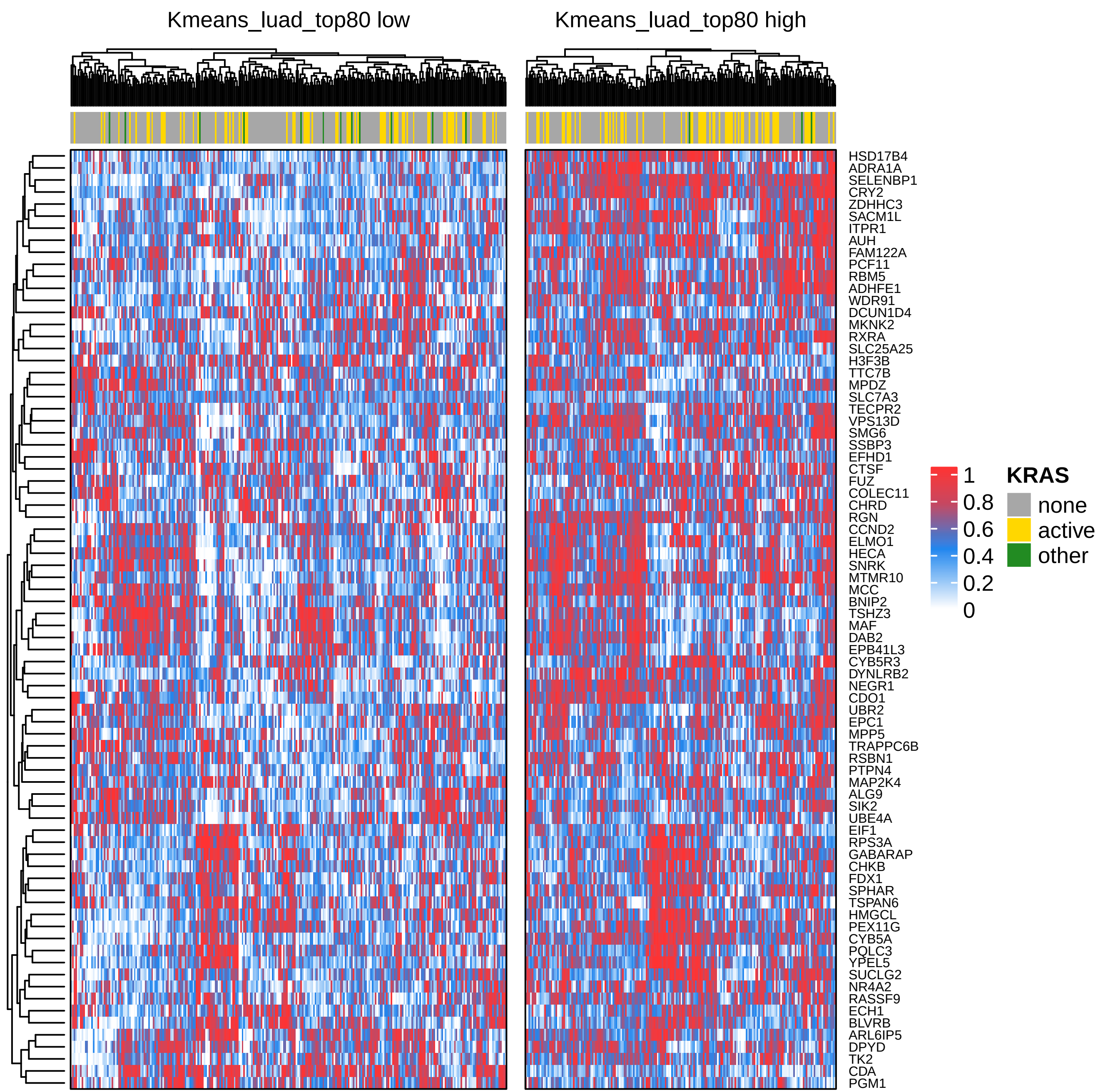
Kmeans_luad_top80 low
Kmeans_luad_top80 high
HSD17B4
ADRA1A
SELENBP1
CRY2
ZDHHC3
SACM1L
ITPR1
AUH
FAM122A
PCF11
RBM5
ADHFE1
WDR91
DCUN1D4
MKNK2
RXRA
SLC25A25
H3F3B
TTC7B
MPDZ
SLC7A3
TECPR2
VPS13D
SMG6
SSBP3
EFHD1
CTSF
FUZ
COLEC11
CHRD
RGN
CCND2
ELMO1
HECA
SNRK
MTMR10
MCC
BNIP2
TSHZ3
MAF
DAB2
EPB41L3
CYB5R3
DYNLRB2
NEGR1
CDO1
UBR2
EPC1
MPP5
TRAPPC6B
RSBN1
PTPN4
MAP2K4
ALG9
SIK2
UBE4A
EIF1
RPS3A
GABARAP
CHKB
FDX1
SPHAR
TSPAN6
HMGCL
PEX11G
CYB5A
PQLC3
YPEL5
SUCLG2
NR4A2
RASSF9
ECH1
BLVRB
ARL6IP5
DPYD
TK2
CDA
PGM1
1
0.8
0.6
0.4
0.2
0
KRAS
none
active
other

All RAS
Colon

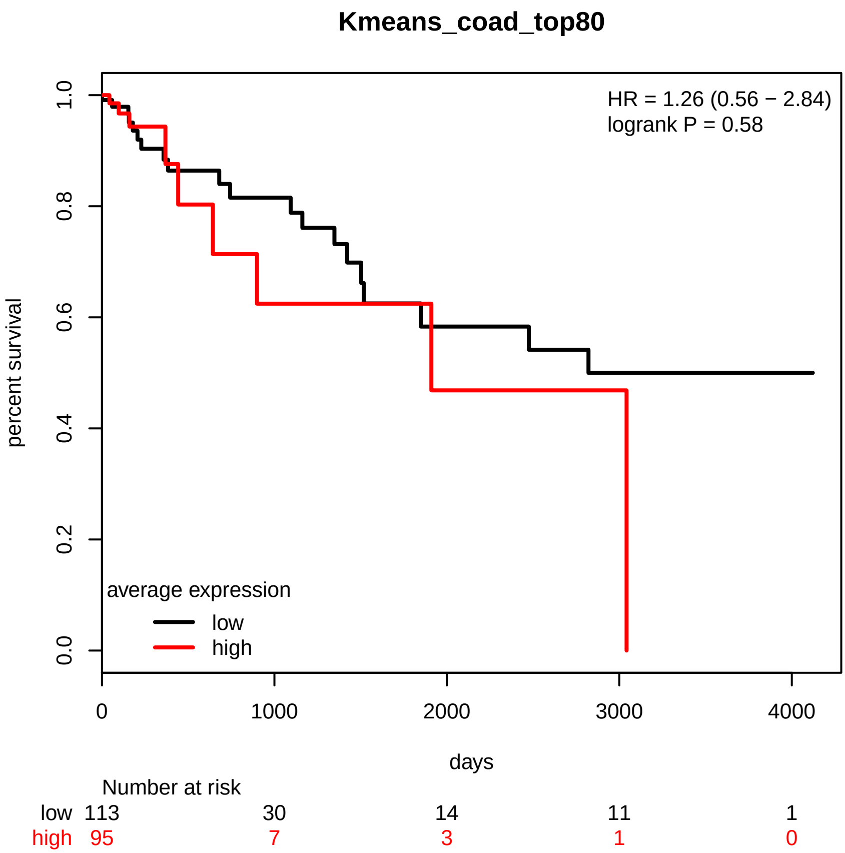

	12/13/61	none/other
low	35	78
high	27	68
pval_less	0.7094	
pval_greater	0.4025	

Kmeans_coad_top55

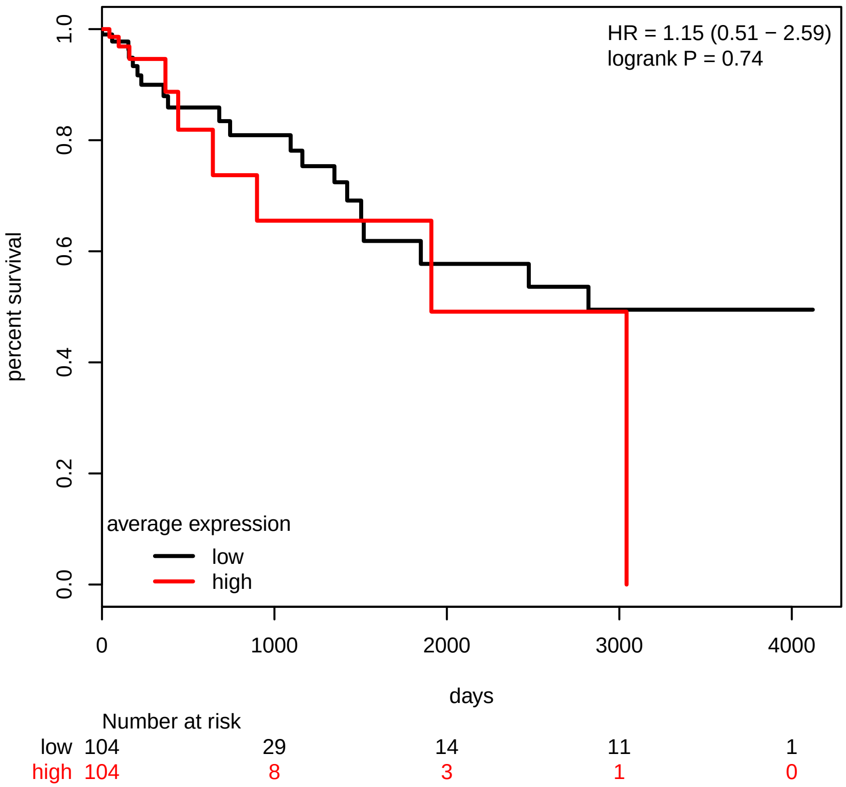

	12/13/61	none/other
low	33	71
high	29	75
pval_less	0.7757	
pval_greater	0.3247	

Kmeans_coad_top25

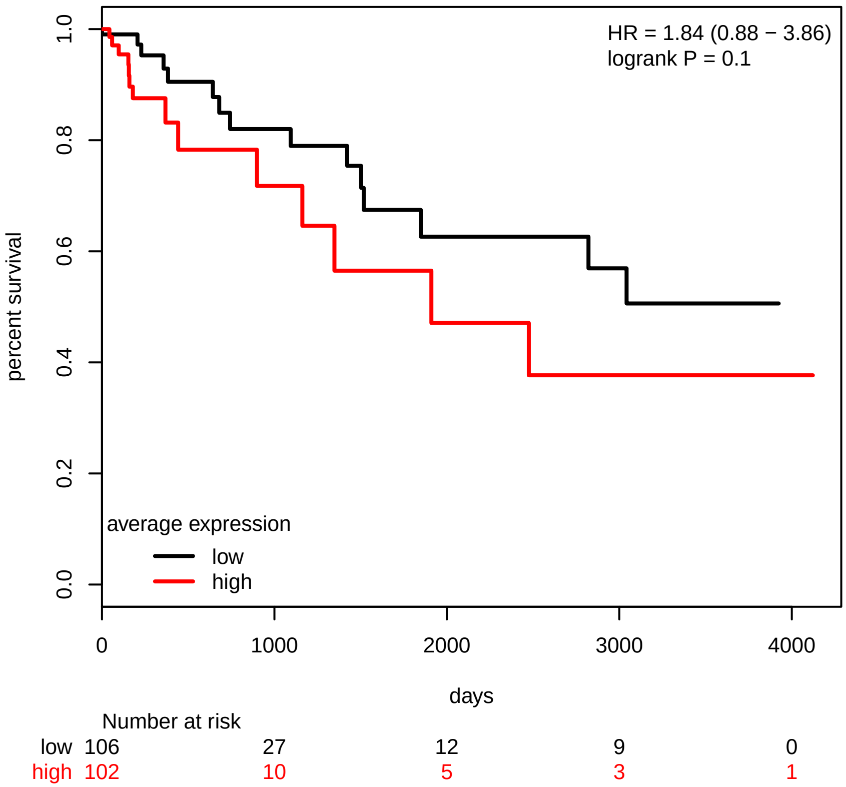

	12/13/61	none/other
low	35	71
high	27	75
pval_less	0.8818	
pval_greater	0.1894	

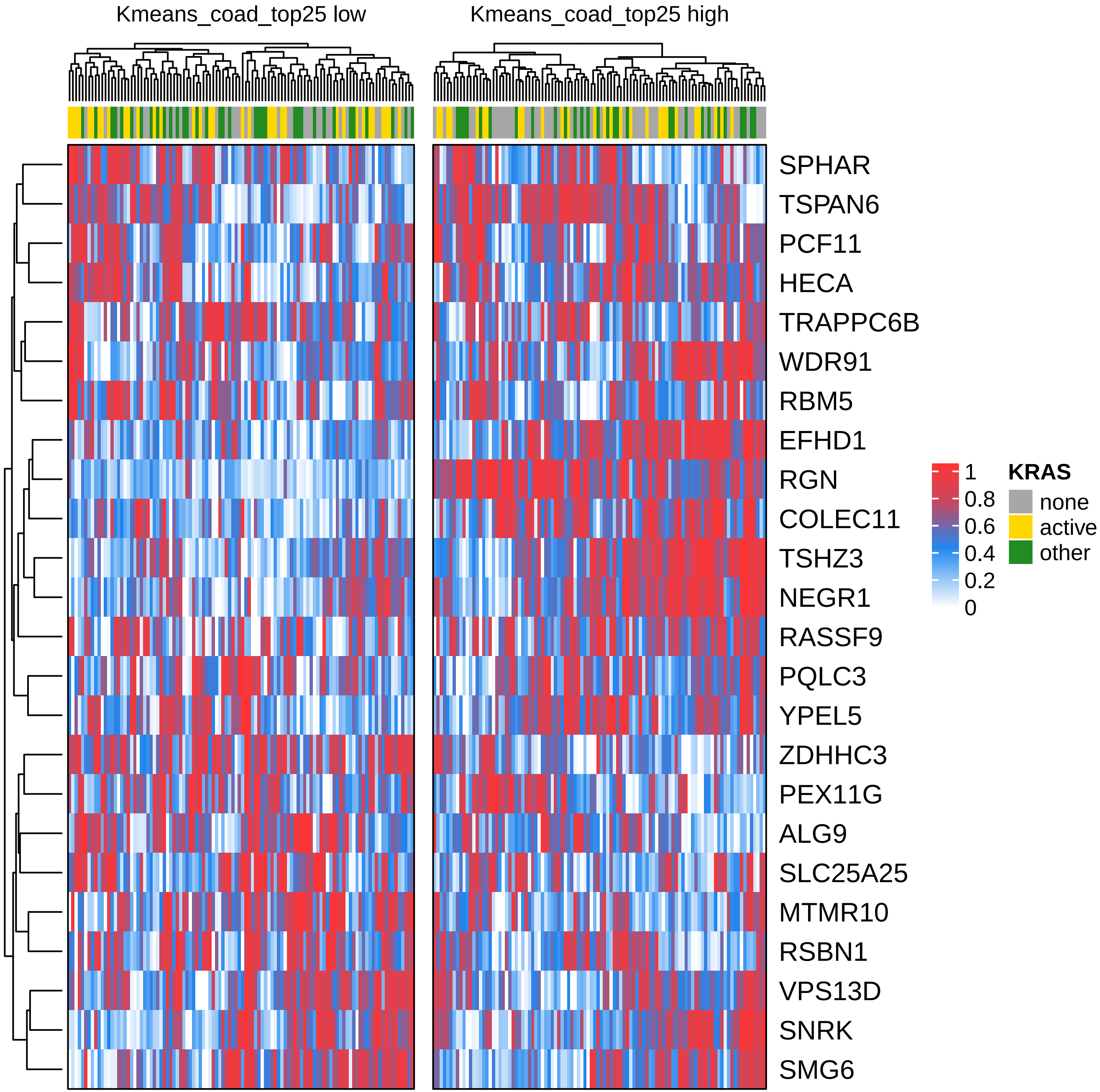
Kmeans_coad_top25 low
Kmeans_coad_top25 high
SPHAR
TSPAN6
PCF11
HECA
TRAPPC6B
WDR91
RBM5
EFHD1
RGN
COLEC11
TSHZ3
NEGR1
RASSF9
PQLC3
YPEL5
ZDHHC3
PEX11G
ALG9
SLC25A25
MTMR10
RSBN1
VPS13D
SNRK
SMG6
1
0.8
0.6
0.4
0.2
0
KRAS
none
active
other

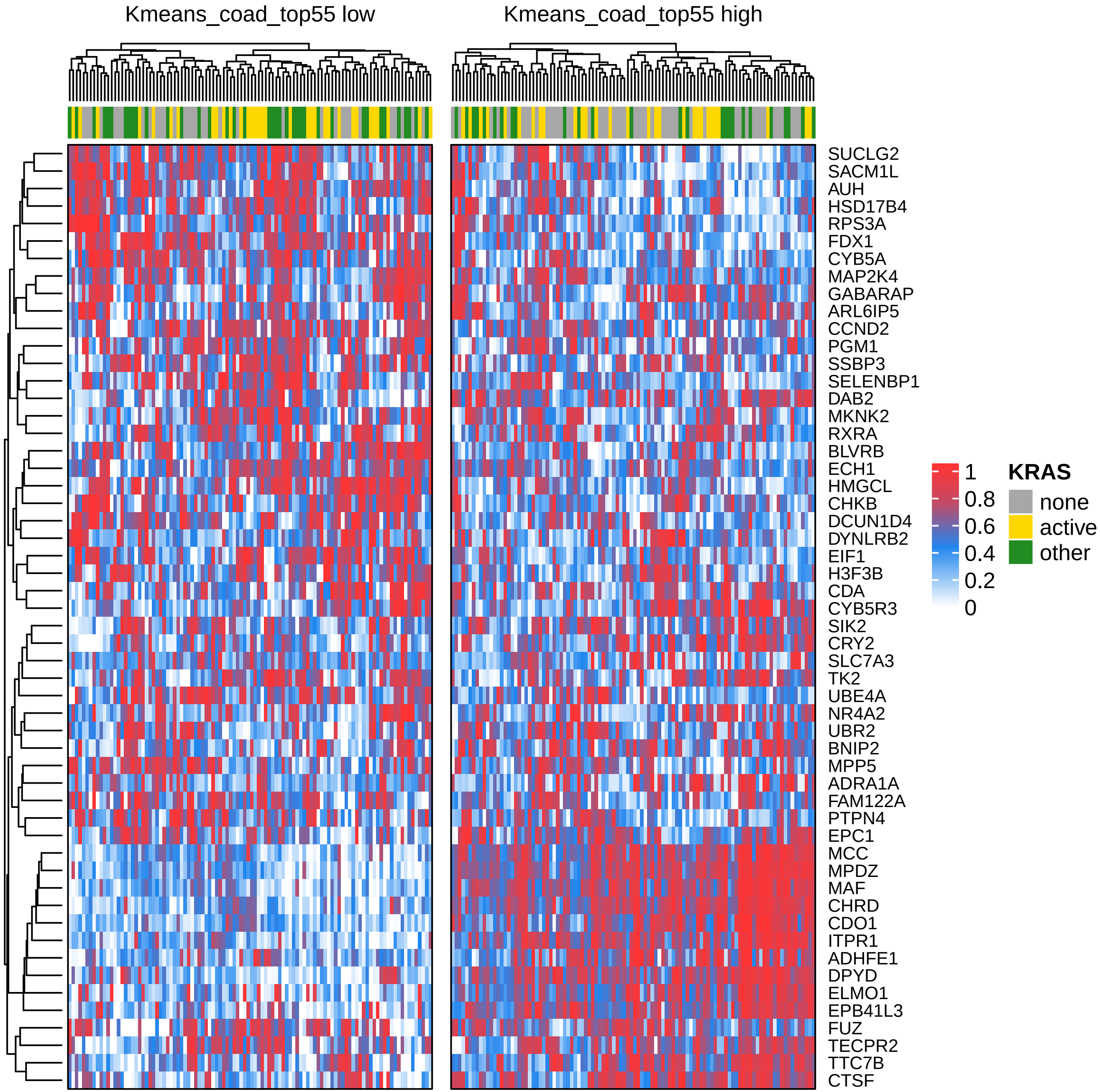
Kmeans_coad_top55 low
Kmeans_coad_top55 high
SUCLG2
SACM1L
AUH
HSD17B4
RPS3A
FDX1
CYB5A
MAP2K4
GABARAP
ARL6IP5
CCND2
PGM1
SSBP3
SELENBP1
DAB2
MKNK2
RXRA
BLVRB
ECH1
HMGCL
CHKB
DCUN1D4
DYNLRB2
EIF1
H3F3B
CDA
CYB5R3
SIK2
CRY2
SLC7A3
TK2
UBE4A
NR4A2
UBR2
BNIP2
MPP5
ADRA1A
FAM122A
PTPN4
EPC1
MCC
MPDZ
MAF
CHRD
CDO1
ITPR1
ADHFE1
DPYD
ELMO1
EPB41L3
FUZ
TECPR2
TTC7B
CTSF
1
0.8
0.6
0.4
0.2
0
KRAS
none
active
other

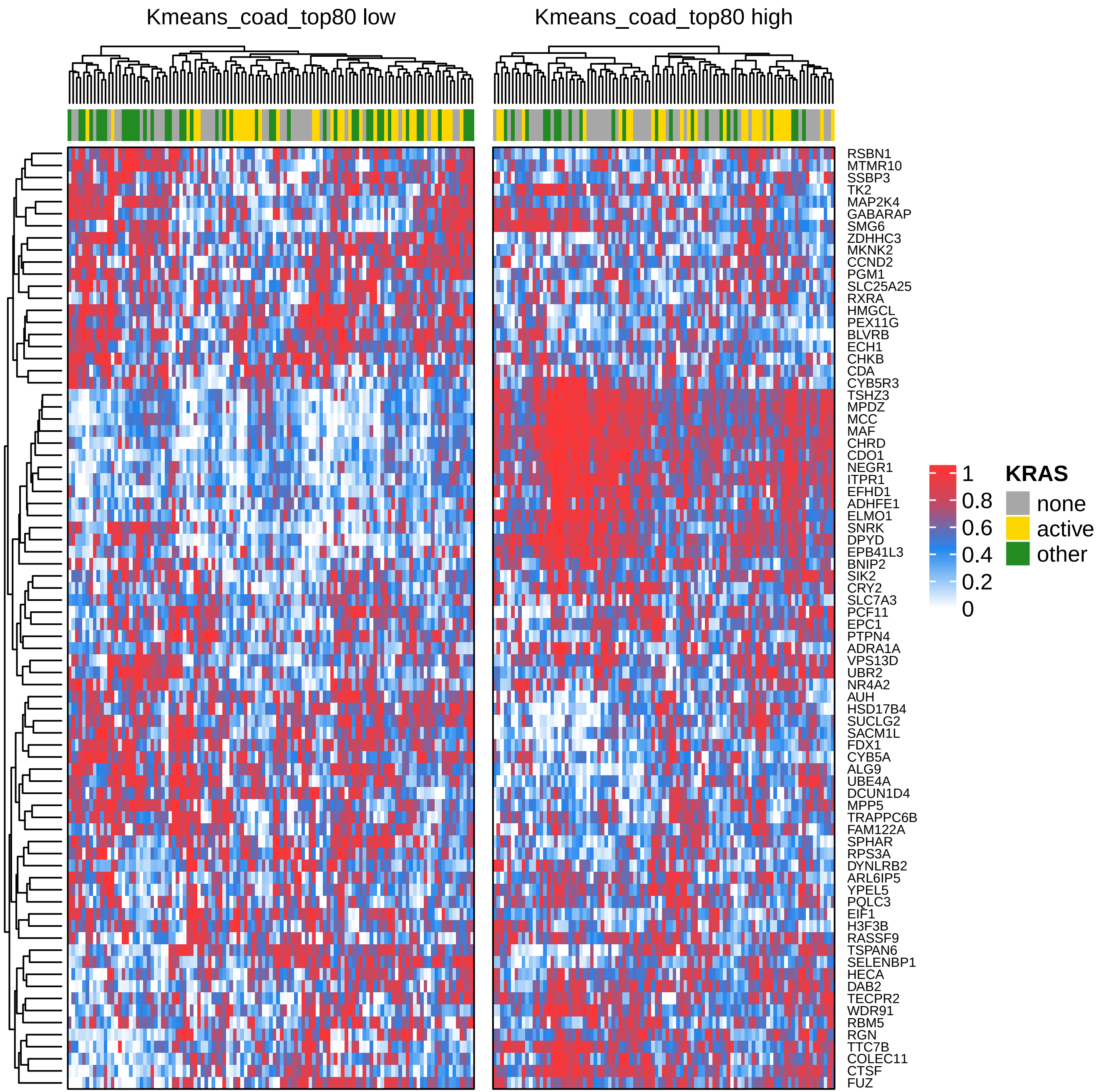
Kmeans_coad_top80 low
Kmeans_coad_top80 high
RSBN1
MTMR10
SSBP3
TK2
MAP2K4
GABARAP
SMG6
ZDHHC3
MKNK2
CCND2
PGM1
SLC25A25
RXRA
HMGCL
PEX11G
BLVRB
ECH1
CHKB
CDA
CYB5R3
TSHZ3
MPDZ
MCC
MAF
CHRD
CDO1
NEGR1
ITPR1
EFHD1
ADHFE1
ELMO1
SNRK
DPYD
EPB41L3
BNIP2
SIK2
CRY2
SLC7A3
PCF11
EPC1
PTPN4
ADRA1A
VPS13D
UBR2
NR4A2
AUH
HSD17B4
SUCLG2
SACM1L
FDX1
CYB5A
ALG9
UBE4A
DCUN1D4
MPP5
TRAPPC6B
FAM122A
SPHAR
RPS3A
DYNLRB2
ARL6IP5
YPEL5
PQLC3
EIF1
H3F3B
RASSF9
TSPAN6
SELENBP1
HECA
DAB2
TECPR2
WDR91
RBM5
RGN
TTC7B
COLEC11
CTSF
FUZ
1
0.8
0.6
0.4
0.2
0
KRAS
none
active
other

All RAS
Pancreatic

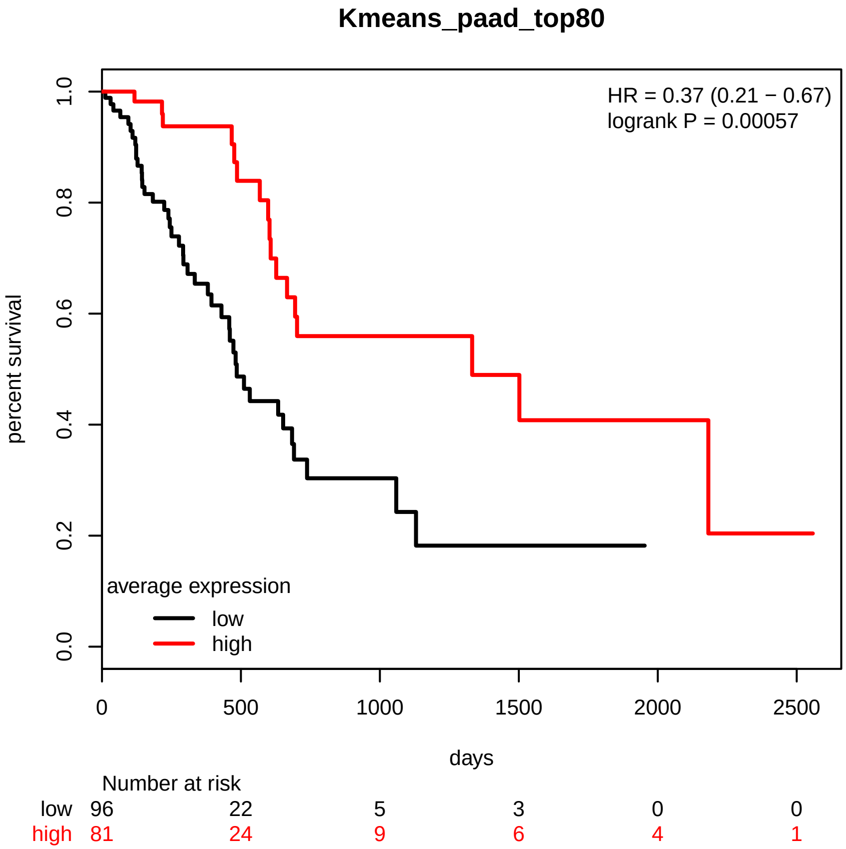

	12/13/61	none/other
low	79	17
high	45	36
pval_less	1	
pval_greater	9.94e−05	

Kmeans_paad_top55

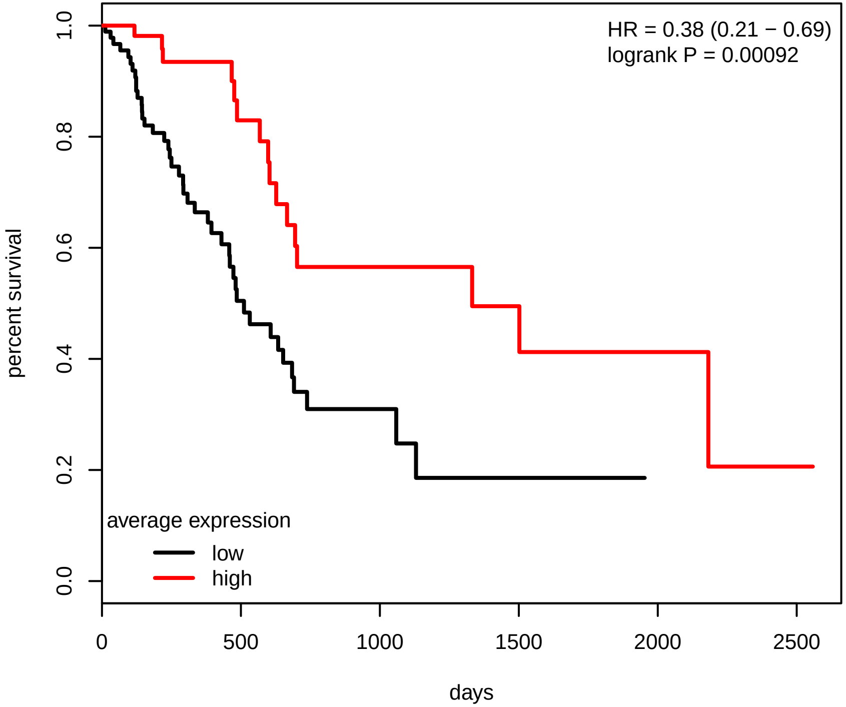

Number at risk

low	99	24	5	3	0	0
high	78	22	9	6	4	1

	12/13/61	none/other
low	81	18
high	43	35
pval_less	1	
pval_greater	0.0001122	

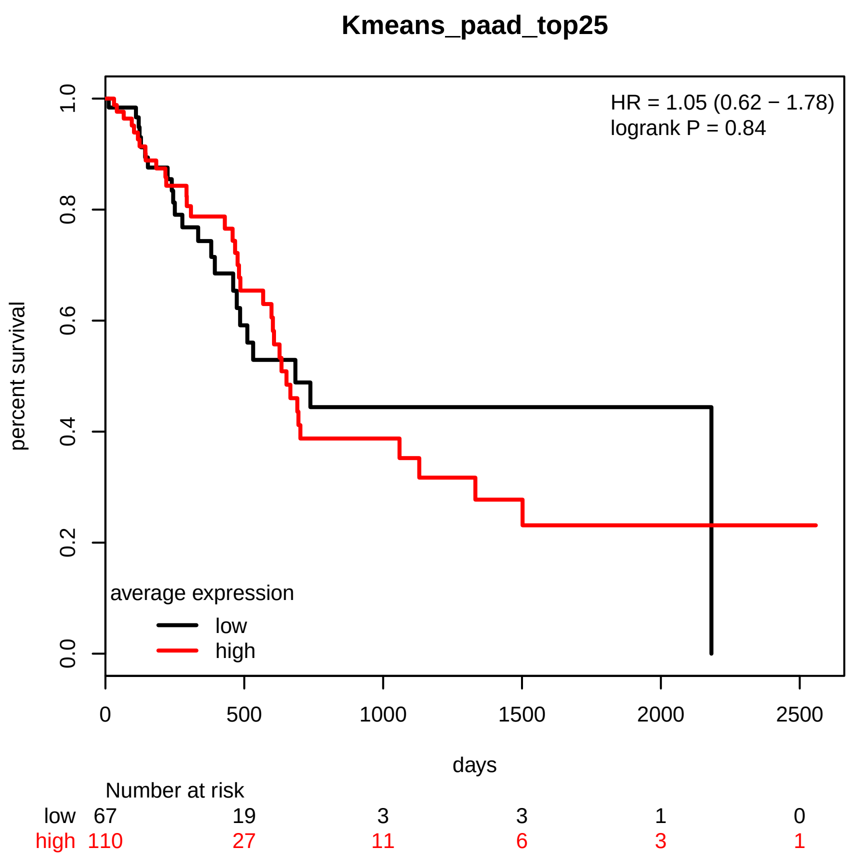

	12/13/61	none/other
low	53	14
high	71	39
pval_less	0.9878	
pval_greater	0.02862	

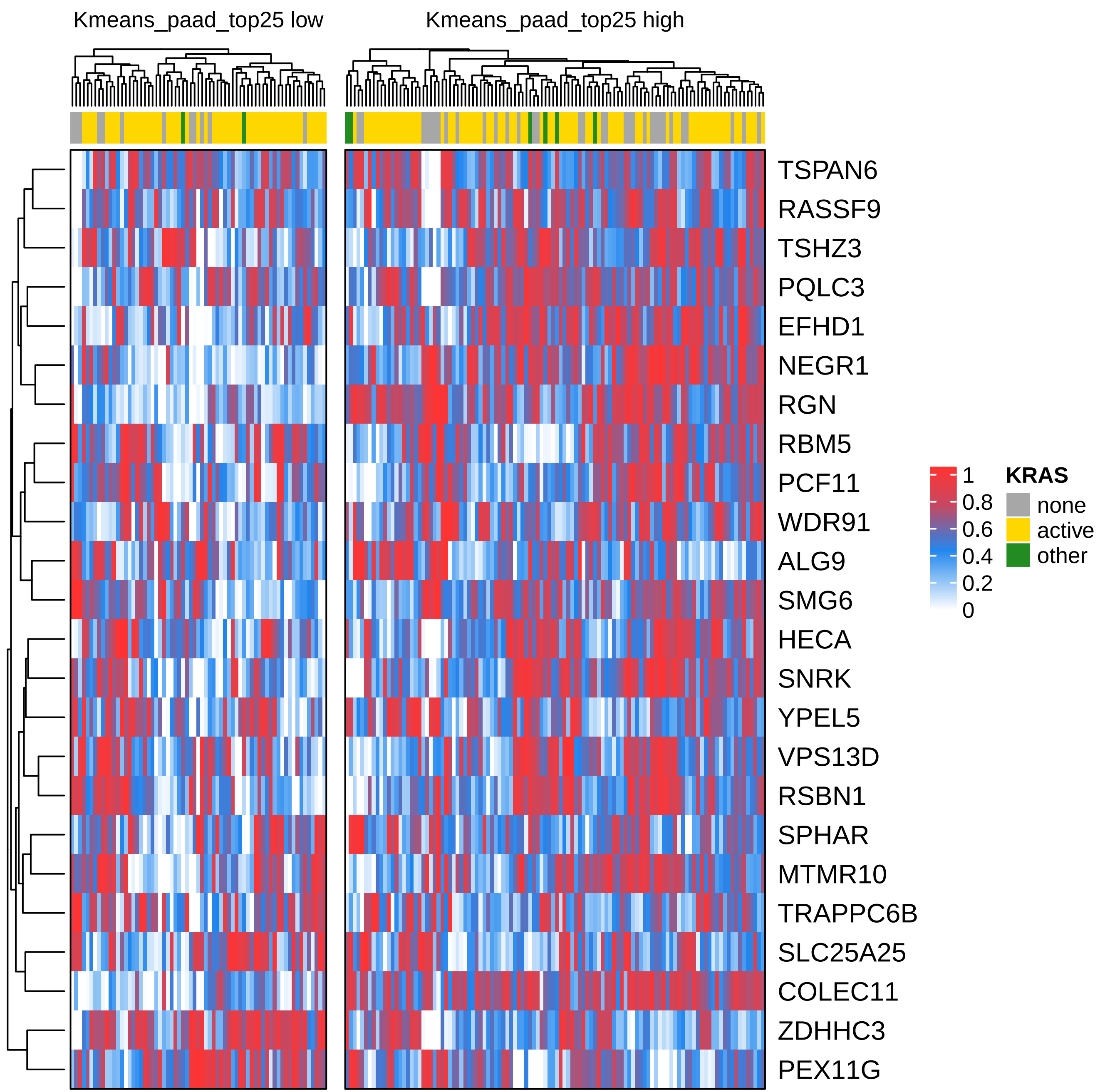

Kmeans_paad_top25 low
Kmeans_paad_top25 high
TSPAN6
RASSF9
TSHZ3
PQLC3
EFHD1
NEGR1
RGN
RBM5
PCF11
WDR91
ALG9
SMG6
HECA
SNRK
YPEL5
VPS13D
RSBN1
SPHAR
MTMR10
TRAPPC6B
SLC25A25
COLEC11
ZDHHC3
PEX11G
1
0.8
0.6
0.4
0.2
0
KRAS
none
active
other

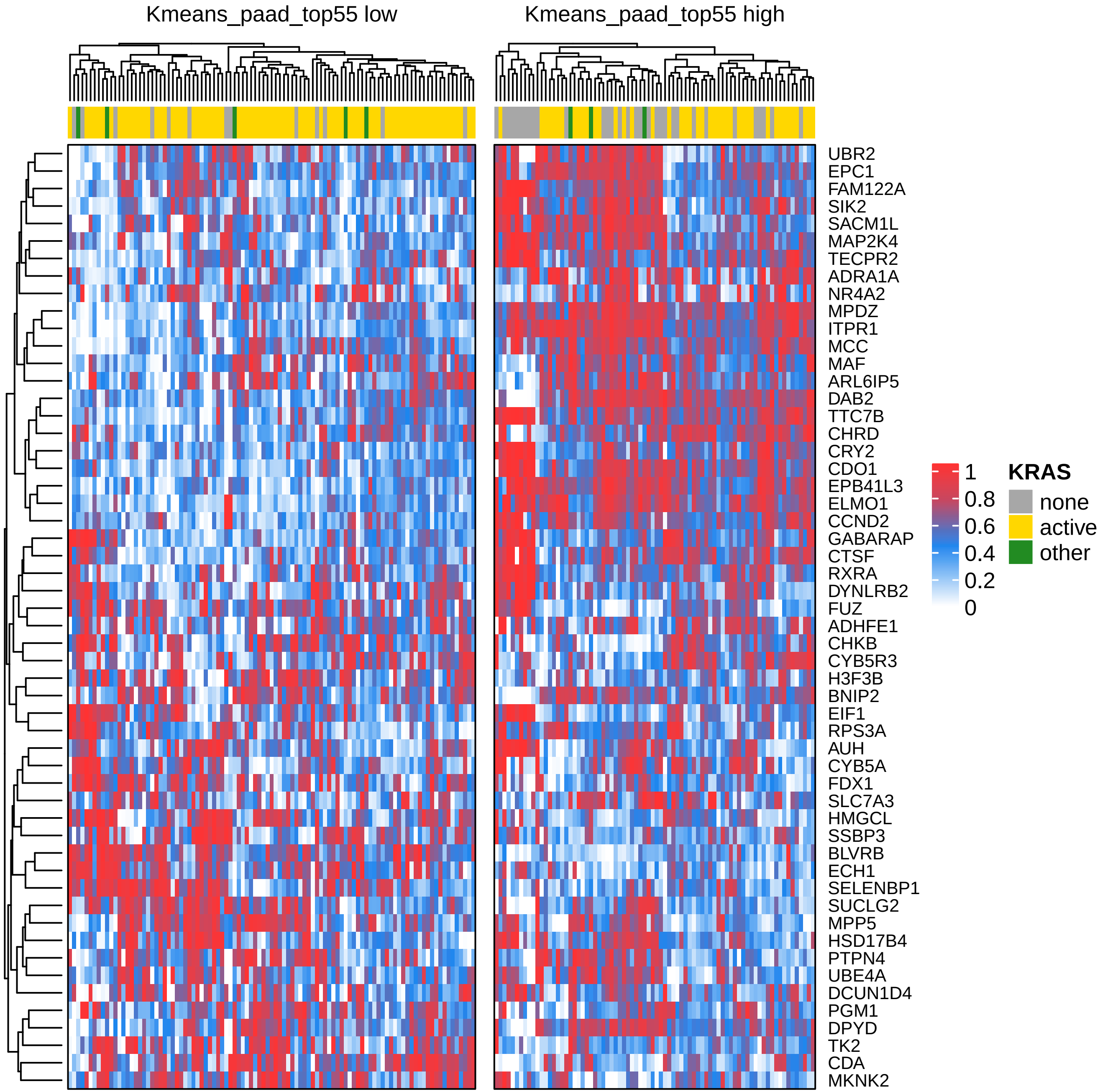

Kmeans_paad_top55 low
Kmeans_paad_top55 high
UBR2
EPC1
FAM122A
SIK2
SACM1L
MAP2K4
TECPR2
ADRA1A
NR4A2
MPDZ
ITPR1
MCC
MAF
ARL6IP5
DAB2
TTC7B
CHRD
CRY2
CDO1
EPB41L3
ELMO1
CCND2
GABARAP
CTSF
RXRA
DYNLRB2
FUZ
ADHFE1
CHKB
CYB5R3
H3F3B
BNIP2
EIF1
RPS3A
AUH
CYB5A
FDX1
SLC7A3
HMGCL
SSBP3
BLVRB
ECH1
SELENBP1
SUCLG2
MPP5
HSD17B4
PTPN4
UBE4A
DCUN1D4
PGM1
DPYD
TK2
CDA
MKNK2
1
0.8
0.6
0.4
0.2
0
KRAS
none
active
other

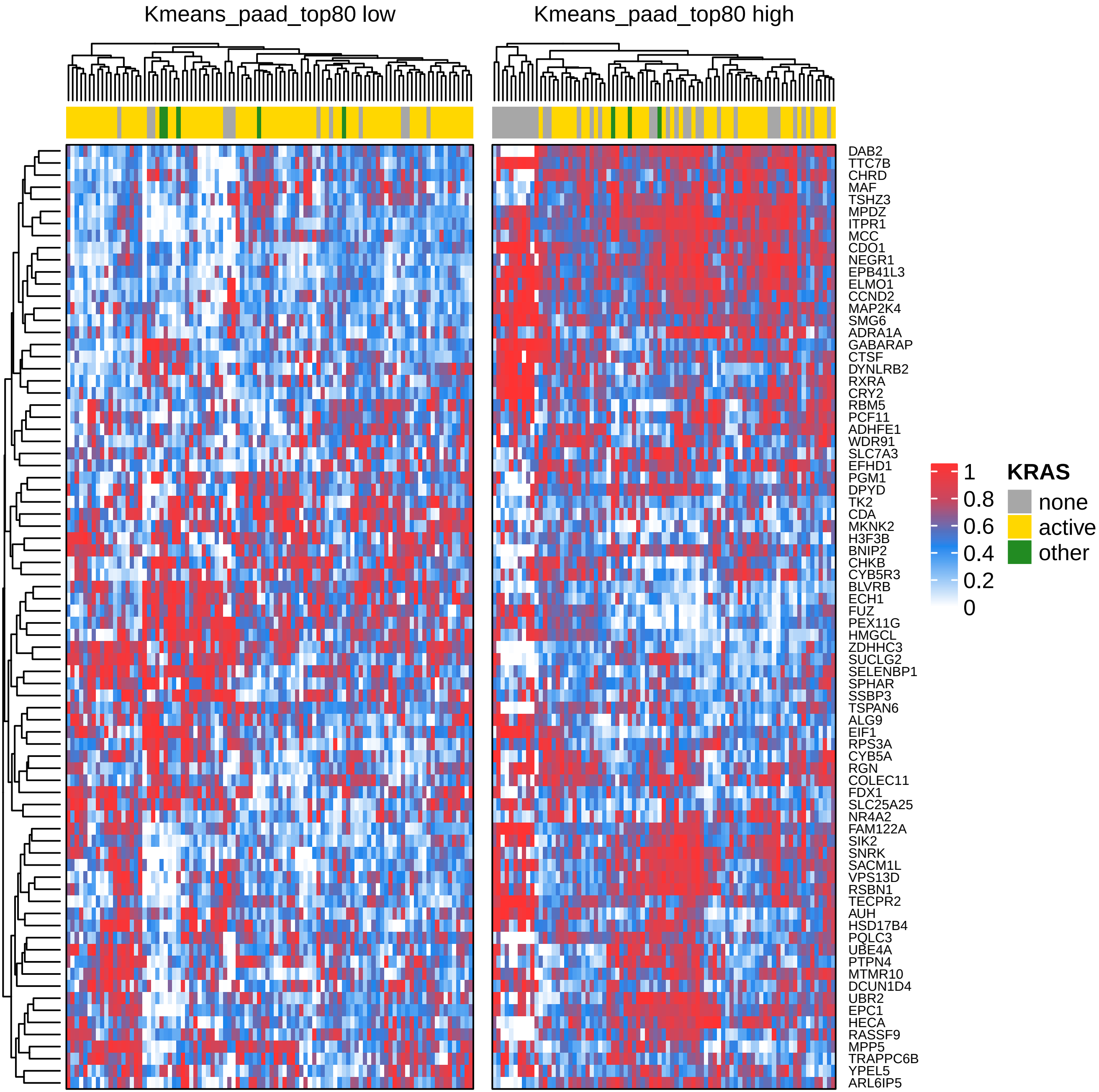
Kmeans_paad_top80 low
Kmeans_paad_top80 high
DAB2
TTC7B
CHRD
MAF
TSHZ3
MPDZ
ITPR1
MCC
CDO1
NEGR1
EPB41L3
ELMO1
CCND2
MAP2K4
SMG6
ADRA1A
GABARAP
CTSF
DYNLRB2
RXRA
CRY2
RBM5
PCF11
ADHFE1
WDR91
SLC7A3
EFHD1
PGM1
DPYD
TK2
CDA
MKNK2
H3F3B
BNIP2
CHKB
CYB5R3
BLVRB
ECH1
FUZ
PEX11G
HMGCL
ZDHHC3
SUCLG2
SELENBP1
SPHAR
SSBP3
TSPAN6
ALG9
EIF1
RPS3A
CYB5A
RGN
COLEC11
FDX1
SLC25A25
NR4A2
FAM122A
SIK2
SNRK
SACM1L
VPS13D
RSBN1
TECPR2
AUH
HSD17B4
PQLC3
UBE4A
PTPN4
MTMR10
DCUN1D4
UBR2
EPC1
HECA
RASSF9
MPP5
TRAPPC6B
YPEL5
ARL6IP5
1
0.8
0.6
0.4
0.2
0
KRAS
none
active
other

(c) Summary of RAS mutations

PAAD	symbol	pval_less	pval_greater	low.mut	low.none_other	high.mut	high.none_other
top80_TCGA_PAAD	KRAS_p.G12	0.999975226	9.94004E-05	79	17	45	36
top80_TCGA_PAAD	KRAS_p.Q61	0.798446303	0.457819572	5	91	3	78
top55_TCGA_PAAD	KRAS_p.G12	0.999971632	0.000112207	81	18	43	35
top55_TCGA_PAAD	KRAS_p.Q61	0.769176733	0.498198994	5	94	3	75
top25_TCGA_PAAD	KRAS_p.G12	0.987848178	0.02862016	53	14	71	39
top25_TCGA_PAAD	KRAS_p.Q61	0.357605303	0.875099762	2	65	6	104

LUAD	symbol	pval_less	pval_greater	low.mut	low.none_other	high.mut	high.none_other
top80_TCGA_LUAD	KRAS_p.G12	0.009622203	0.994574883	65	209	67	130
top80_TCGA_LUAD	KRAS_p.G13	0.865857055	0.379649759	5	269	2	195
top80_TCGA_LUAD	KRAS_p.Q61	1	0.113478593	4	270	0	197
top80_TCGA_LUAD	NRAS_p.Q61	1	0.337904865	2	272	0	197
top80_TCGA_LUAD	HRAS_p.Q61	1	0.581740977	1	273	0	197
top55_TCGA_LUAD	KRAS_p.G12	0.037025742	0.976808675	62	192	70	147
top55_TCGA_LUAD	KRAS_p.G13	0.705642404	0.585935949	4	250	3	214
top55_TCGA_LUAD	KRAS_p.Q61	1	0.083654834	4	250	0	217
top55_TCGA_LUAD	NRAS_p.Q61	1	0.290292271	2	252	0	217
top55_TCGA_LUAD	HRAS_p.Q61	1	0.539278132	1	253	0	217
top25_TCGA_LUAD	KRAS_p.G12	0.885213998	0.159603727	67	153	65	186
top25_TCGA_LUAD	KRAS_p.G13	0.825630395	0.427865501	4	216	3	248
top25_TCGA_LUAD	KRAS_p.Q61	0.953092146	0.263937739	3	217	1	250
top25_TCGA_LUAD	HRAS_p.Q61	1	0.467091295	1	219	0	251
top25_TCGA_LUAD	NRAS_p.Q61	0.782355333	0.716537923	1	219	1	250

COAD	symbol	pval_less	pval_greater	low.mut	low.none_other	high.mut	high.none_other
top80_TCGA_COAD	KRAS_p.G12	0.694768164	0.423287857	31	82	24	71
top80_TCGA_COAD	KRAS_p.G13	0.756999401	0.404255412	13	100	9	86
top80_TCGA_COAD	KRAS_p.Q61	0.848334307	0.42656515	4	109	2	93
top80_TCGA_COAD	NRAS_p.G12	0.95458977	0.243298845	4	109	1	94
top80_TCGA_COAD	NRAS_p.G13	1	0.543269231	1	112	0	95
top80_TCGA_COAD	NRAS_p.Q61	0.434944962	0.906366015	1	112	2	93
top55_TCGA_COAD	KRAS_p.G12	0.735170667	0.376690763	29	75	26	78
top25_TCGA_COAD	KRAS_p.G13	0.750193795	0.411084247	12	92	10	94
top25_TCGA_COAD	NRAS_p.G12	0.970248616	0.184458584	4	100	1	103
top25_TCGA_COAD	KRAS_p.Q61	0.658547904	0.658547904	3	101	3	101
top25_TCGA_COAD	NRAS_p.G13	1	0.5	1	103	0	104
top25_TCGA_COAD	NRAS_p.Q61	0.5	0.876811594	1	103	2	102
top25_TCGA_COAD	KRAS_p.G12	0.920400959	0.137439378	32	74	23	79
top25_TCGA_COAD	KRAS_p.G13	0.848994778	0.281292315	13	93	9	93
top25_TCGA_COAD	KRAS_p.Q61	0.640167721	0.676569482	3	103	3	99
top25_TCGA_COAD	NRAS_p.G12	0.481799119	0.827324209	2	104	3	99
top25_TCGA_COAD	NRAS_p.Q61	0.869494864	0.514491401	2	104	1	101
top25_TCGA_COAD	NRAS_p.G13	1	0.509615385	1	105	0	102
